# Supplementary figures and images for: Upregulated expression of ubiquitin ligase TRIM21 promotes PKM2 nuclear translocation and astrocyte activation in experimental autoimmune encephalomyelitis
Source: eLife. 2024 Sep 12;13:RP98181. doi: 10.7554/eLife.98181 (PMC11392529; doi:10.7554/eLife.98181)

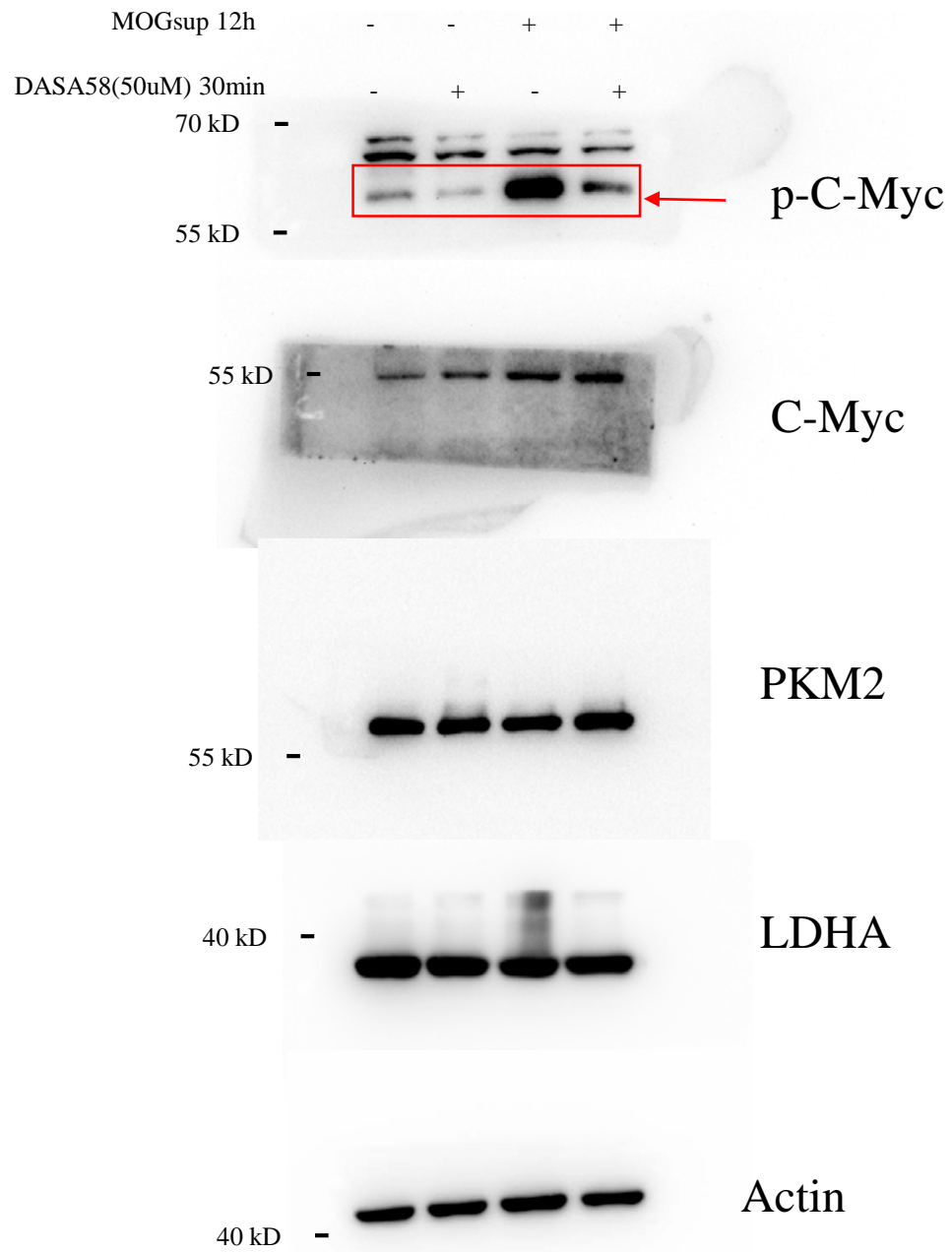

Supplement: Figure 2—source data 2. [file elife-98181-fig2-data2.pdf]

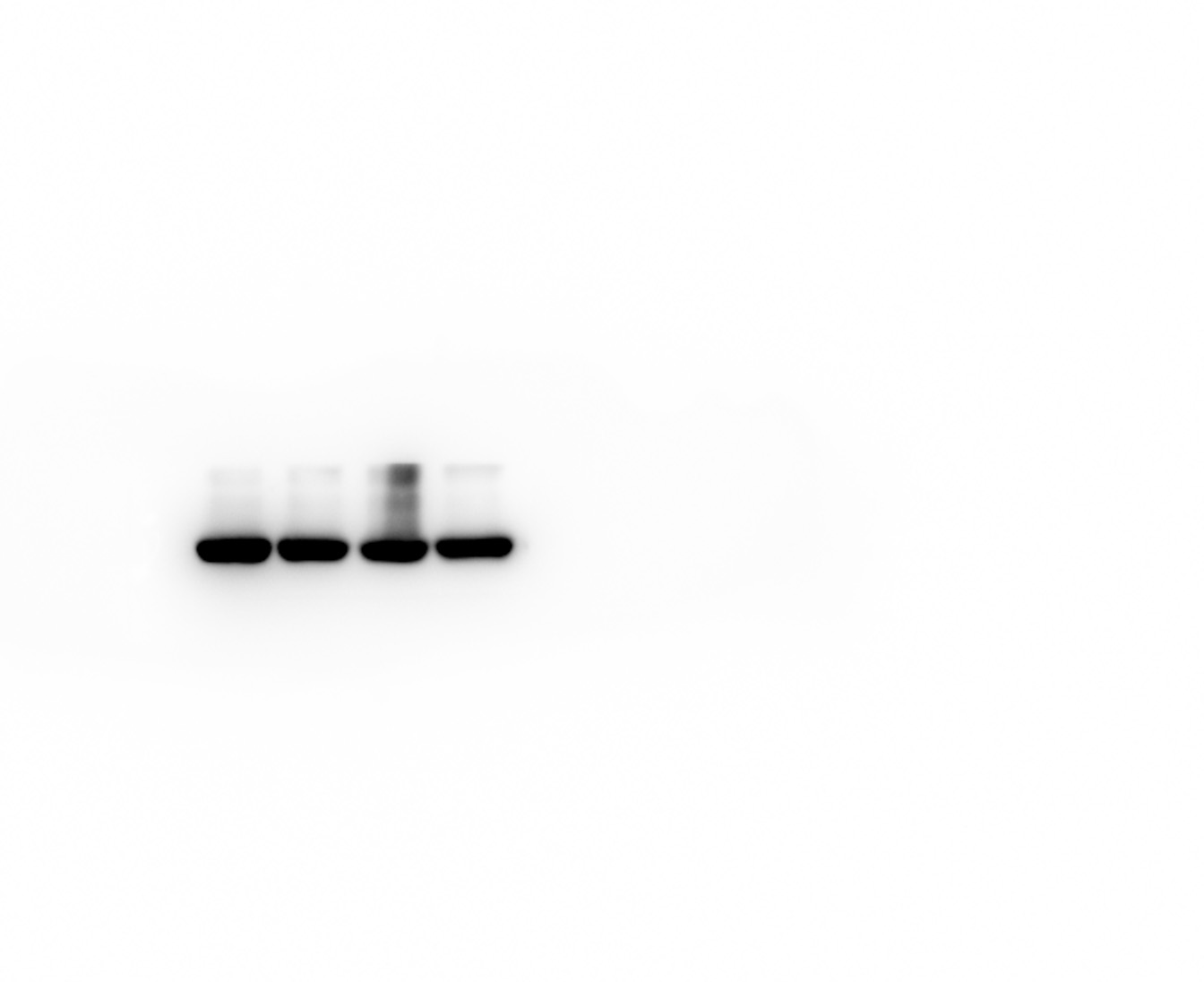

Supplement: Figure 2—source data 3. [file elife-98181-fig2-data3.zip › Figure 2-souce data 3 (LDHA).tif]

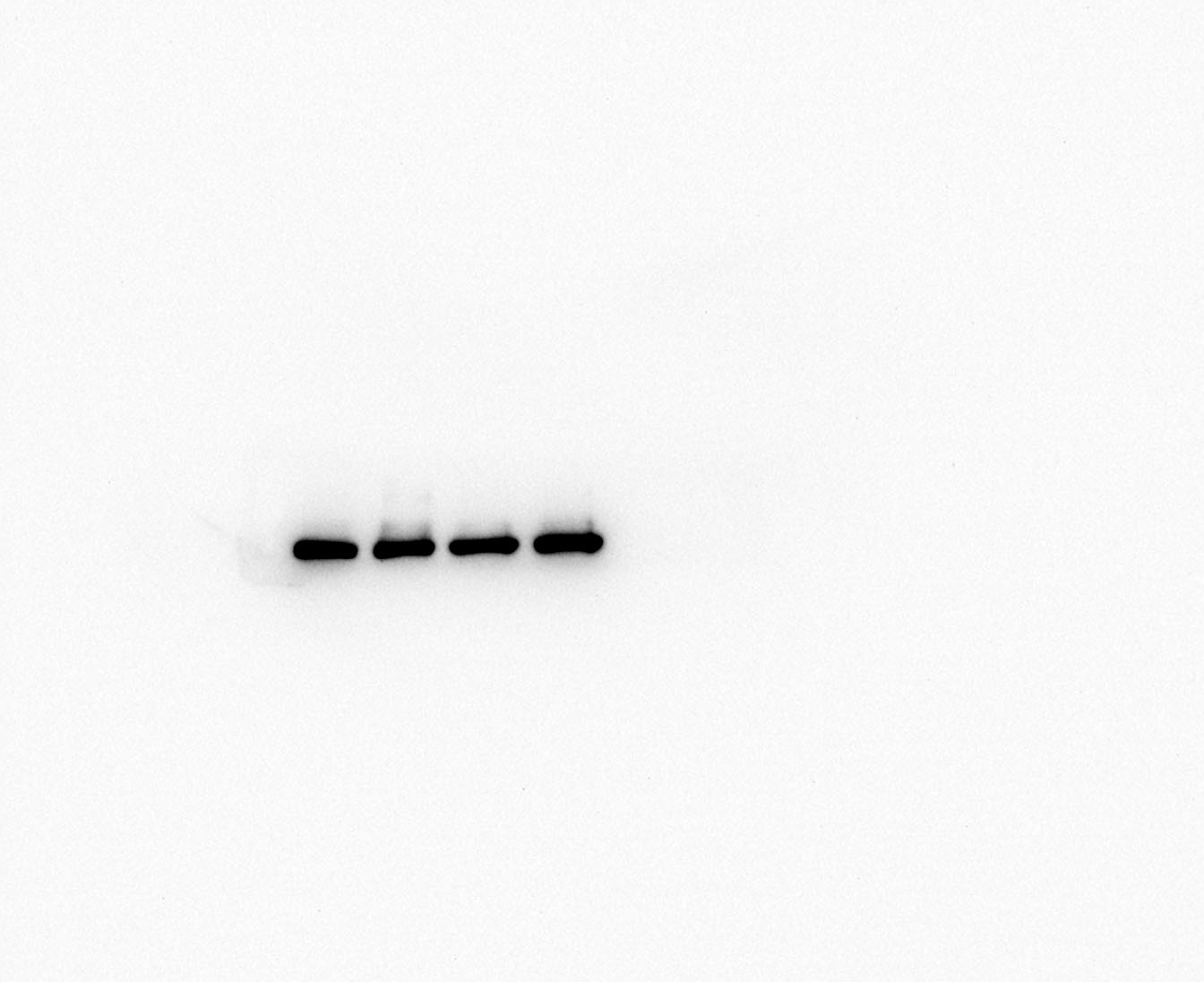

Supplement: Figure 2—source data 3. [file elife-98181-fig2-data3.zip › Figure 2-souce data 3 (PKM2).tif]

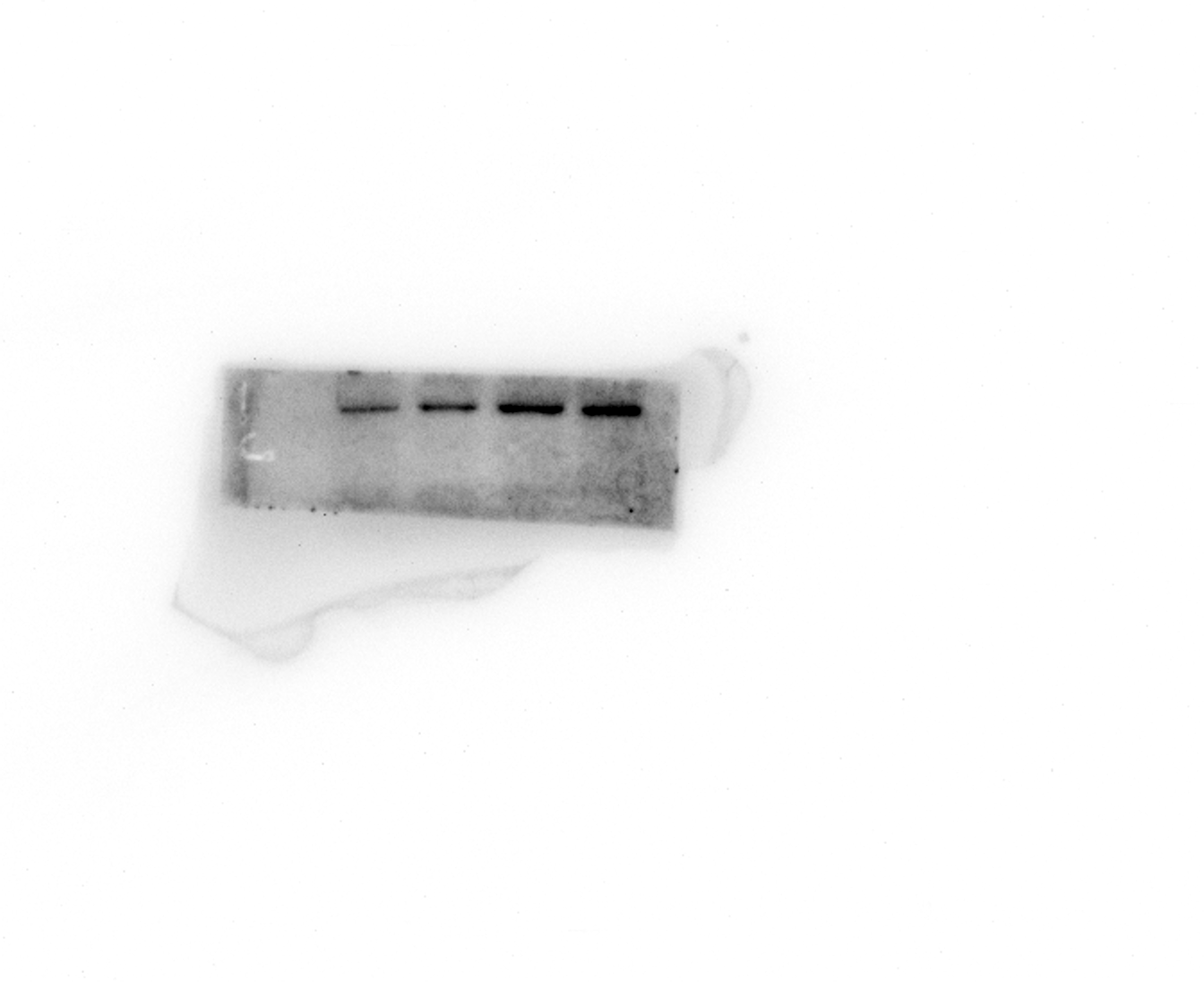

Supplement: Figure 2—source data 3. [file elife-98181-fig2-data3.zip › Figure 2-souce data 3 (c-myc).tif]

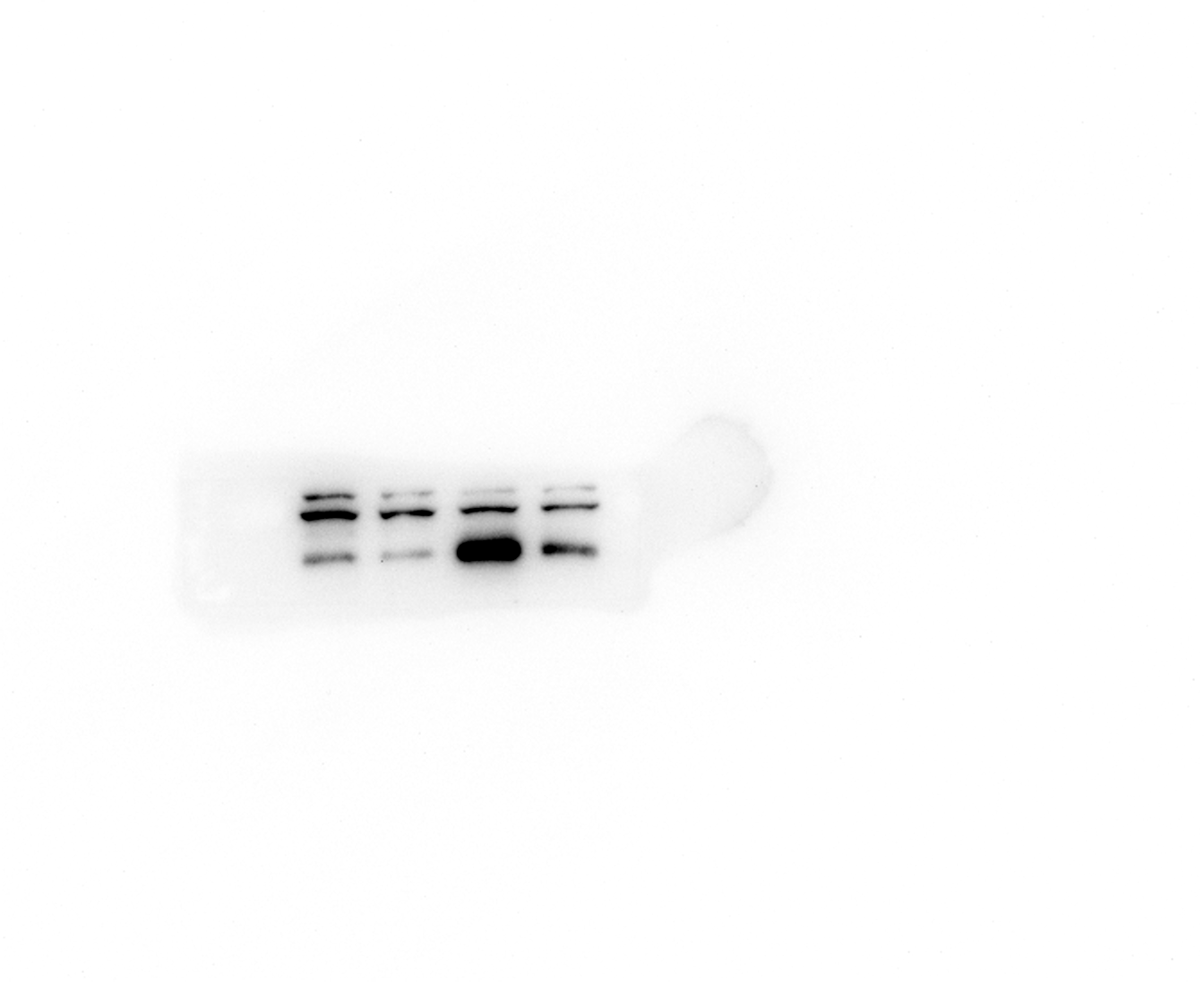

Supplement: Figure 2—source data 3. [file elife-98181-fig2-data3.zip › Figure 2-souce data 3 (p-c-myc).tif]

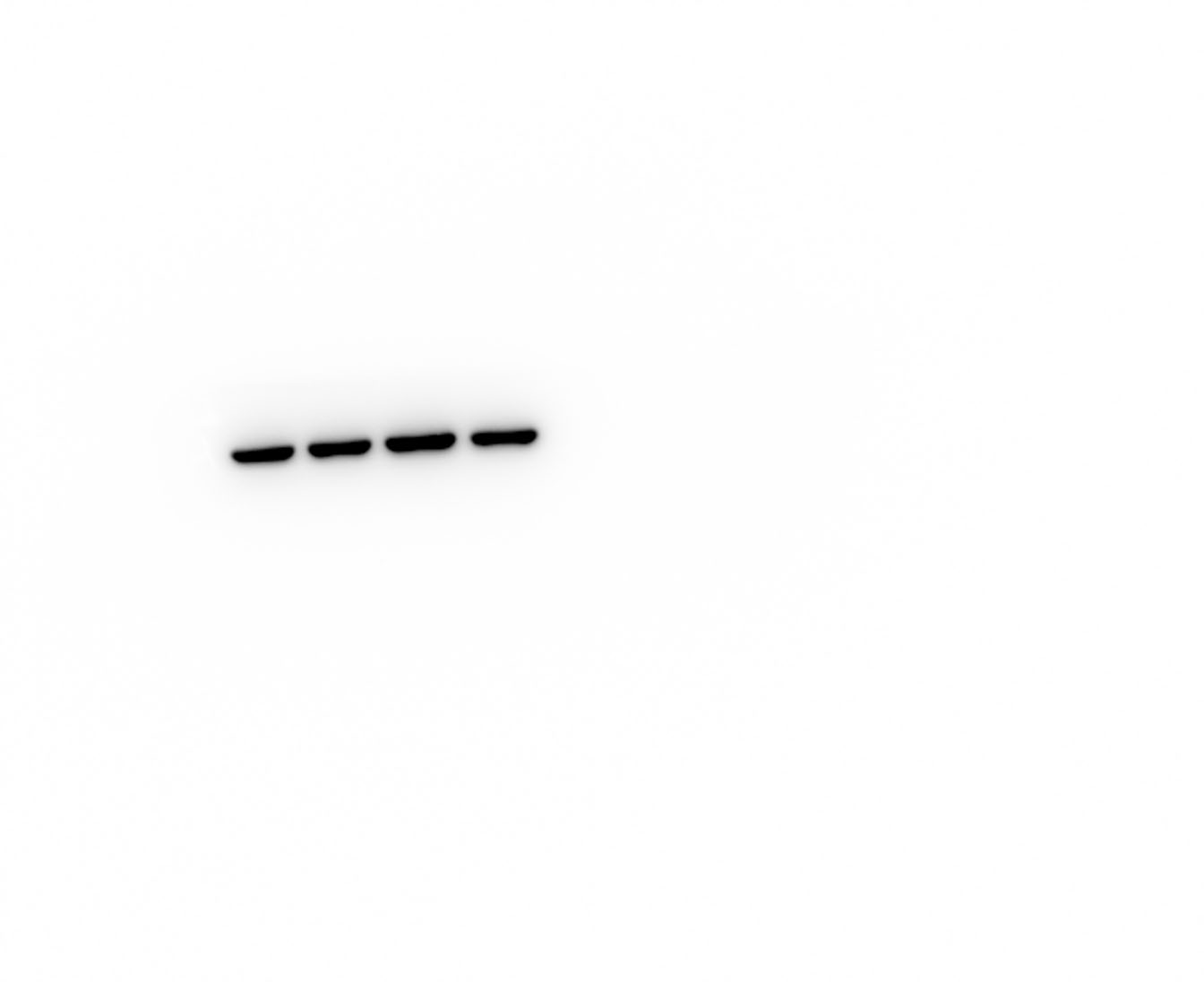

Supplement: Figure 2—source data 3. [file elife-98181-fig2-data3.zip › Figure 2-souce data 3 (Actin).jpg]

|                    |   |   |   |   |
|--------------------|---|---|---|---|
| MOGsup 12h         | - | - | + | + |
| DASA58(50uM) 30min | - | + | - | + |

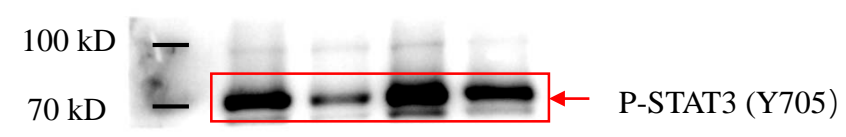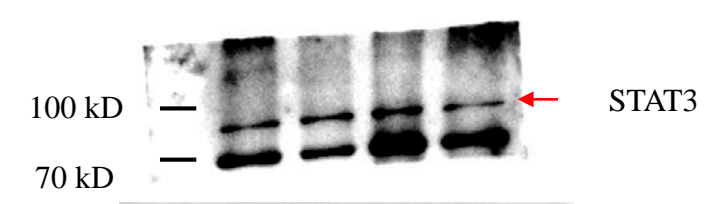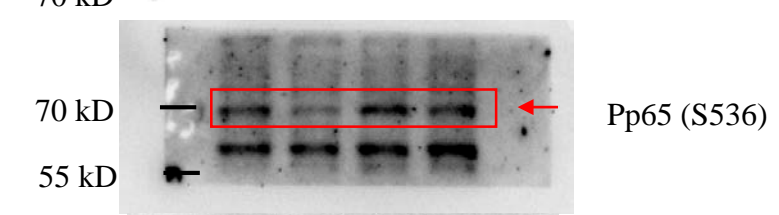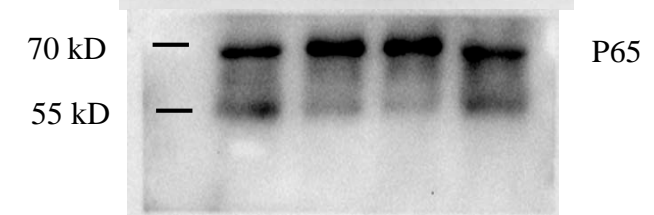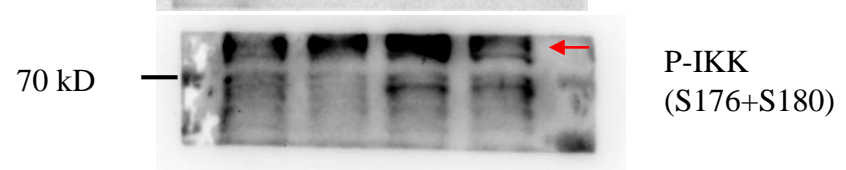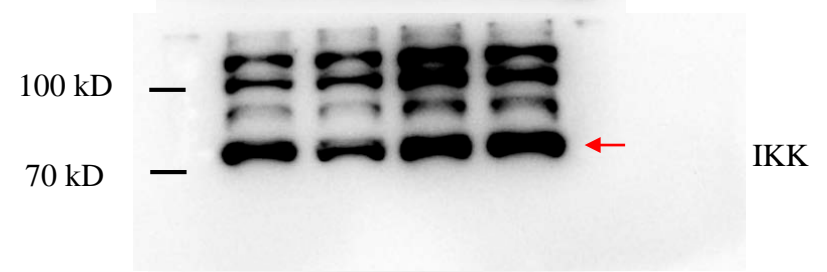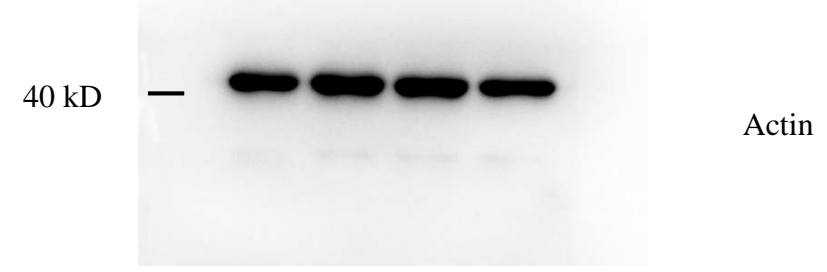

Supplement: Figure 3—source data 2. [file elife-98181-fig3-data2.pdf]

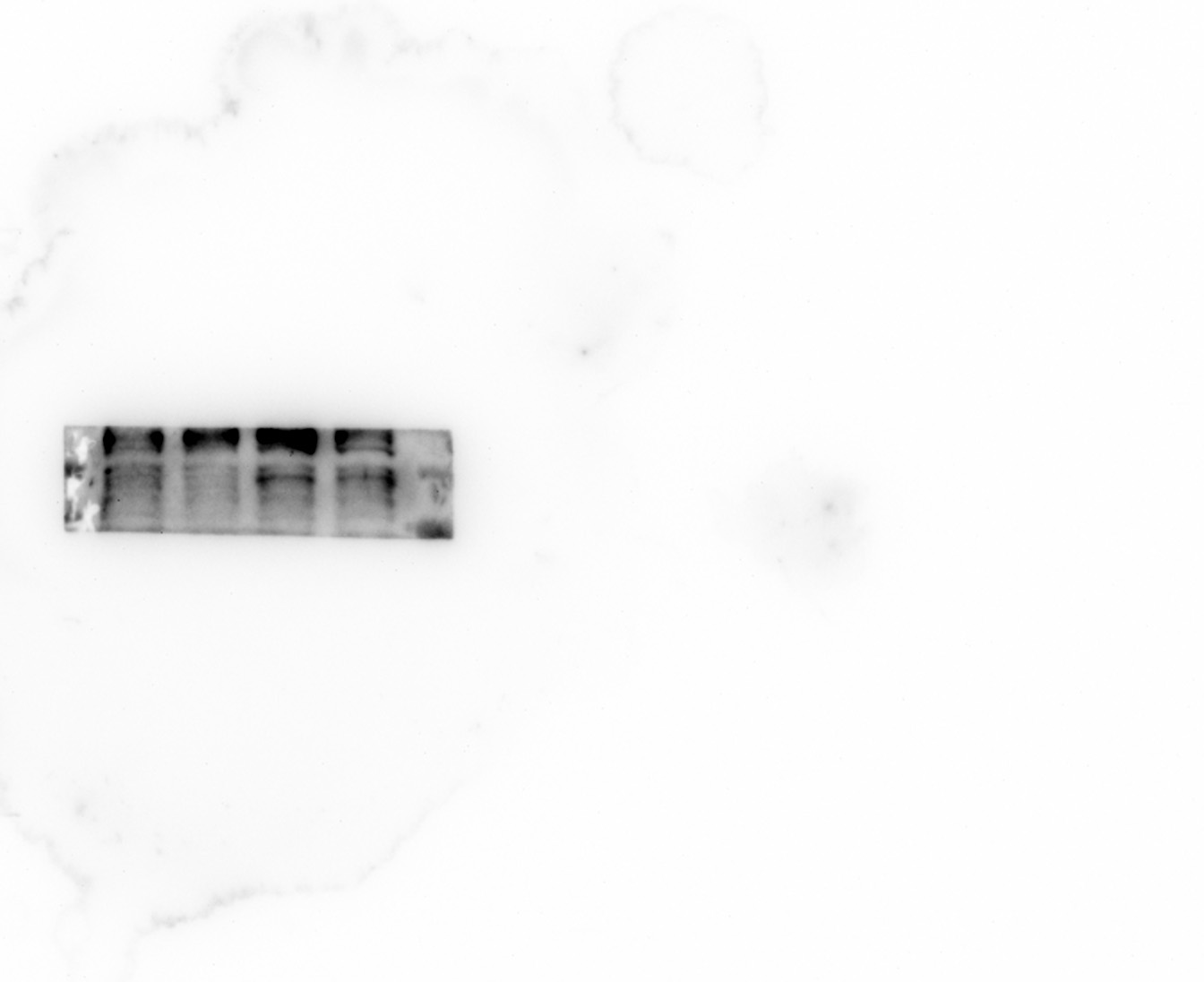

Supplement: Figure 3—source data 3. [file elife-98181-fig3-data3.zip › Figure 3-source data 3 ú¿P-IKKú⌐.jpg]

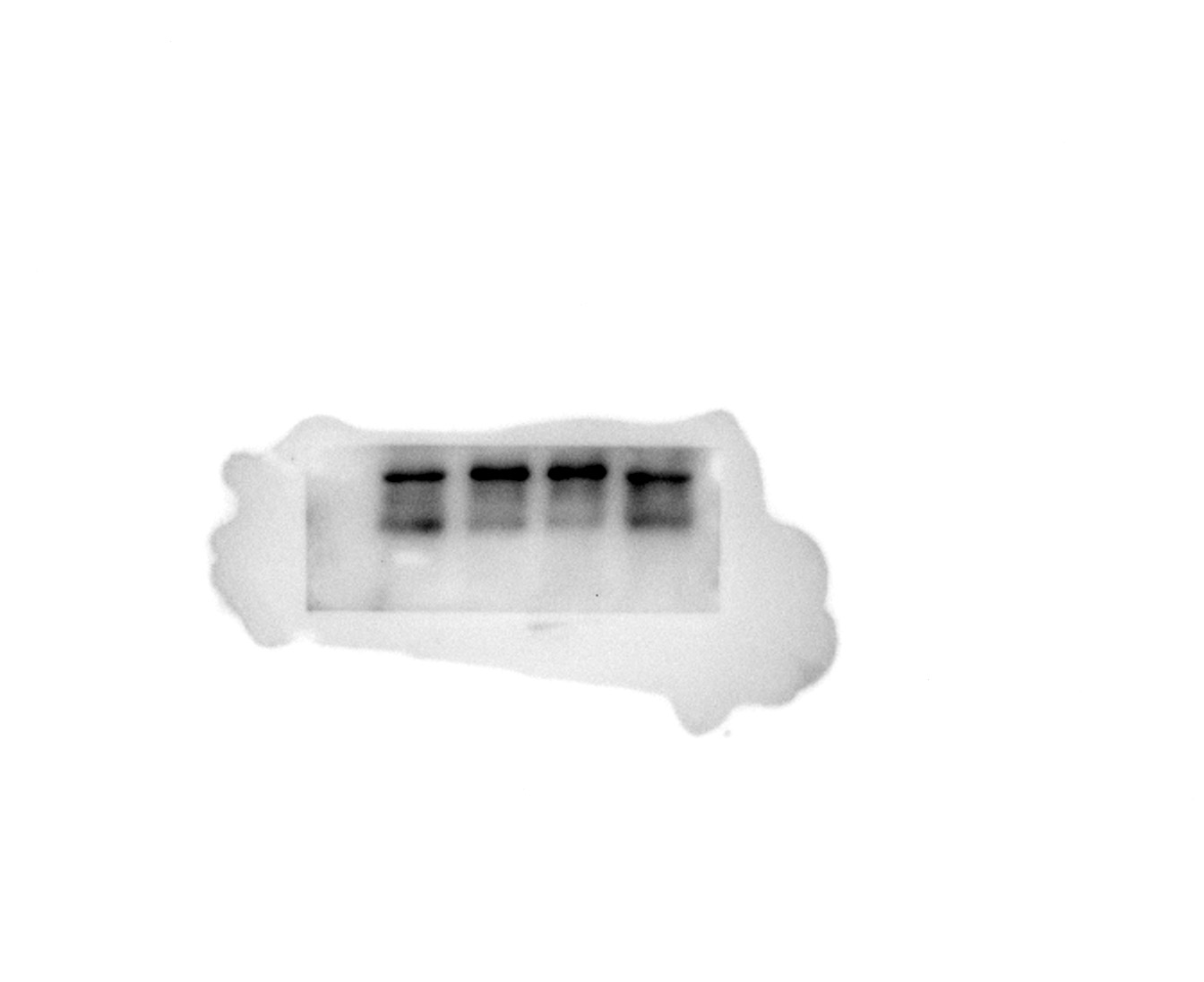

Supplement: Figure 3—source data 3. [file elife-98181-fig3-data3.zip › Figure 3-source data 3 ú¿P65ú⌐.jpg]

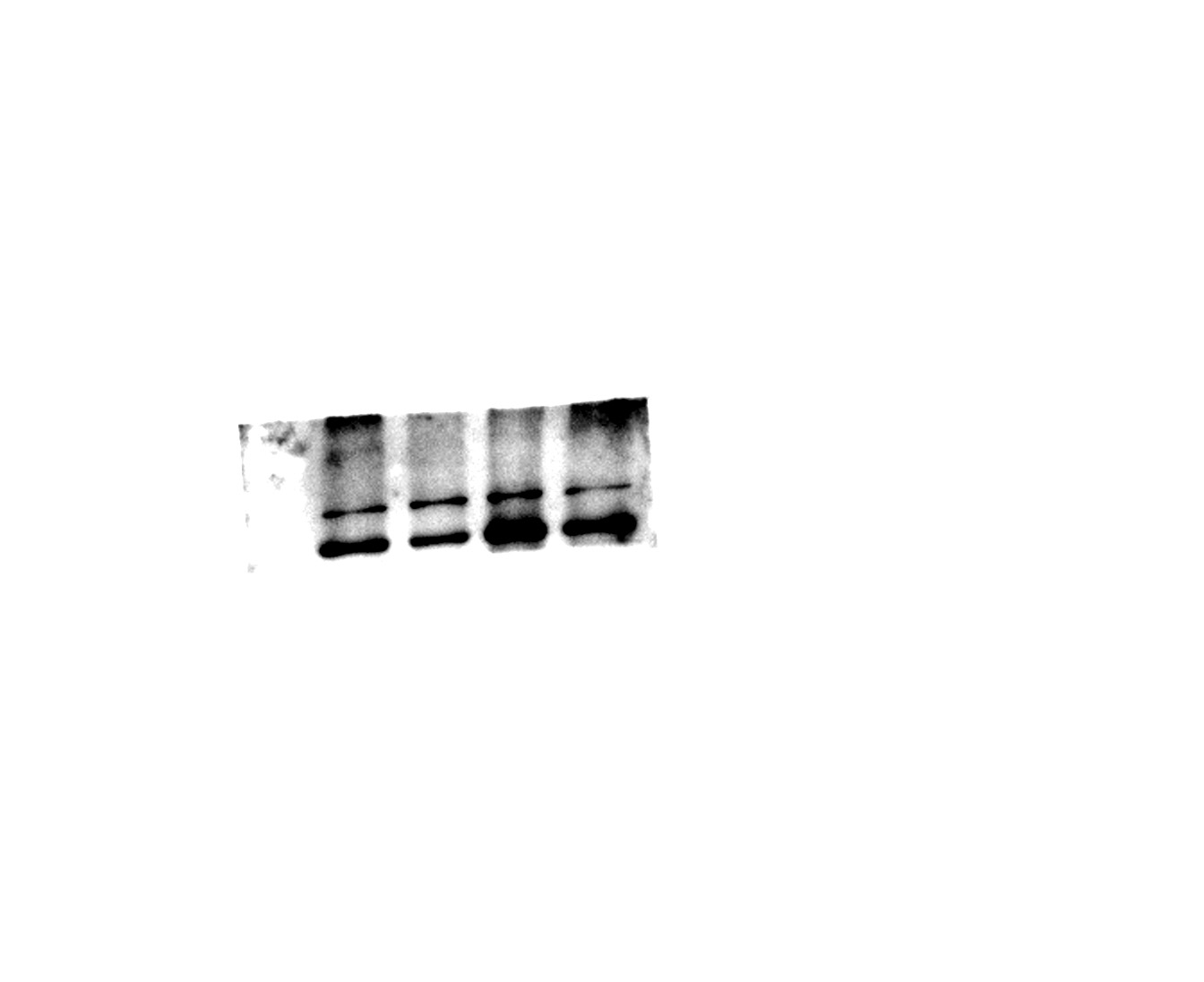

Supplement: Figure 3—source data 3. [file elife-98181-fig3-data3.zip › Figure 3-source data 3 ú¿STAT3ú⌐.jpg]

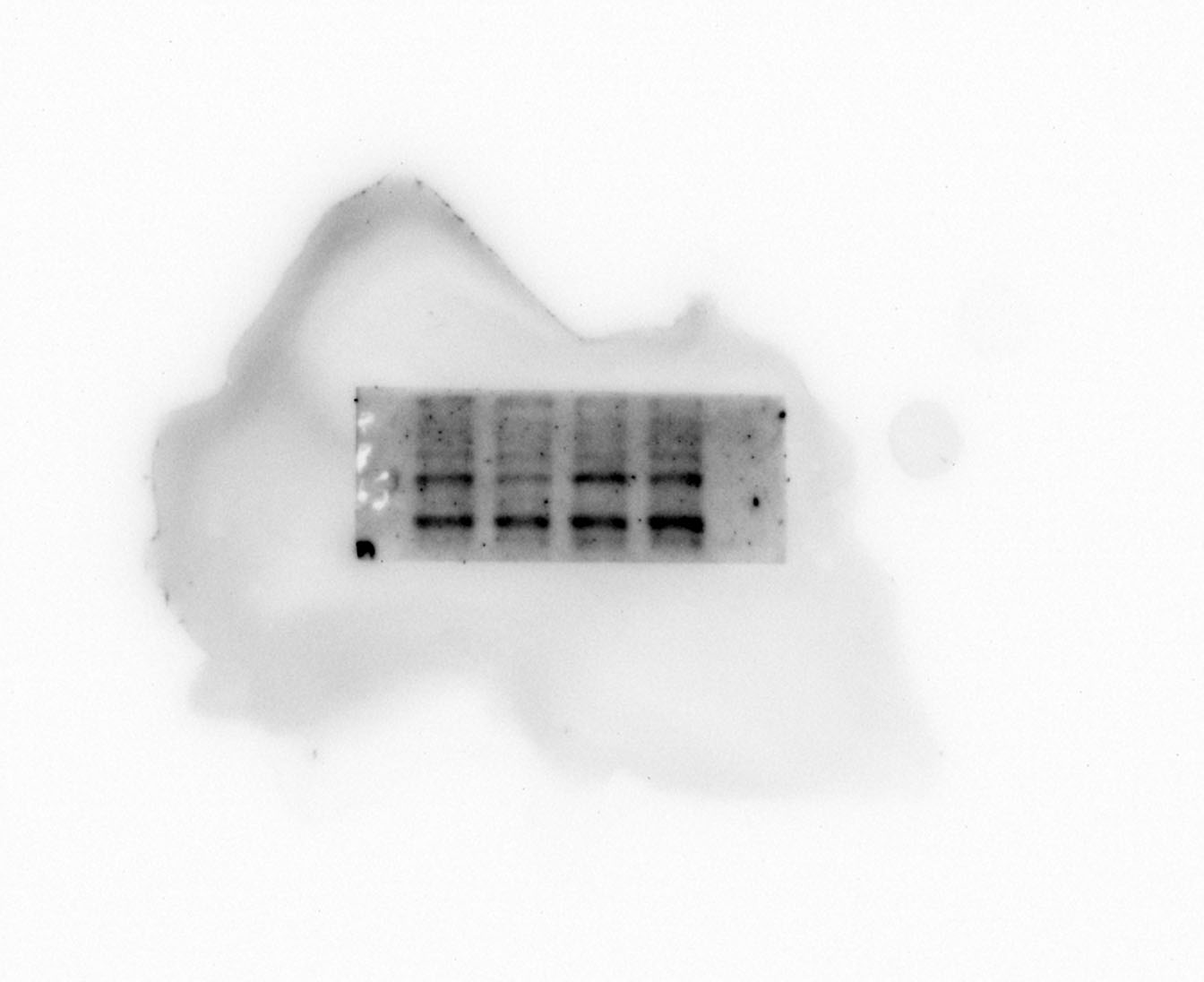

Supplement: Figure 3—source data 3. [file elife-98181-fig3-data3.zip › Figure 3-source data 3 ú¿pp65ú⌐.jpg]

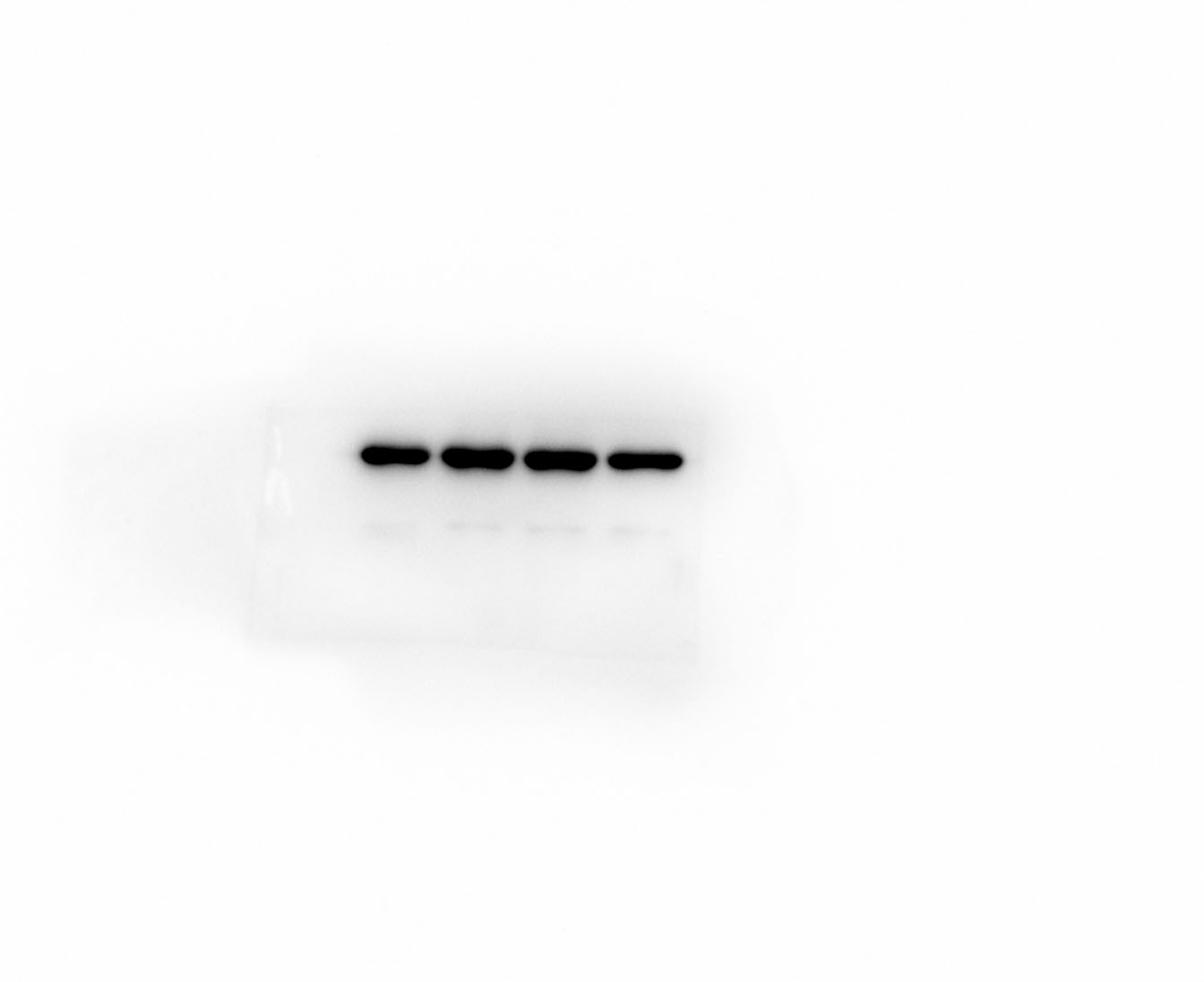

Supplement: Figure 3—source data 3. [file elife-98181-fig3-data3.zip › Figure 3-source data 3ú¿Actinú⌐.jpg]

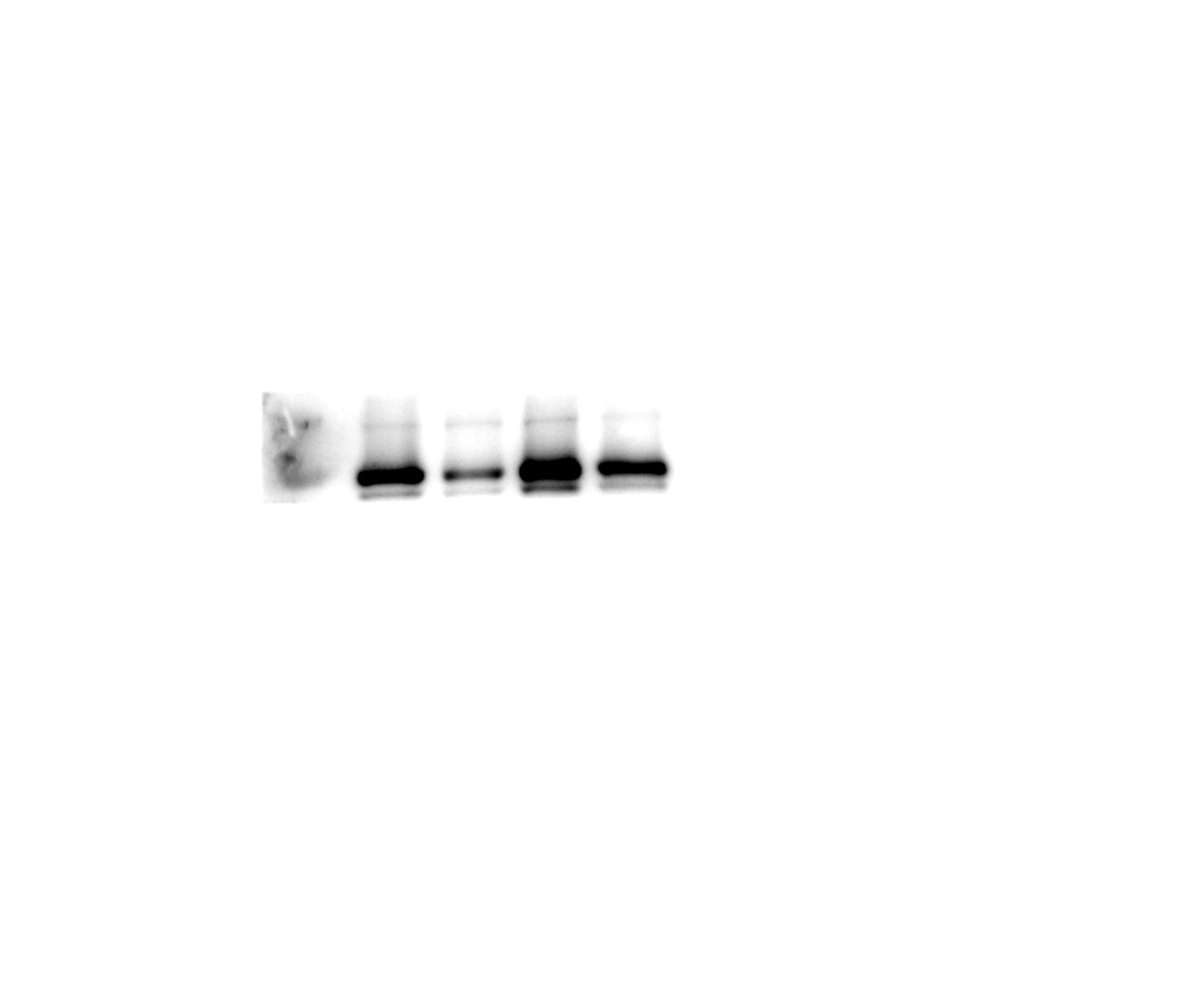

Supplement: Figure 3—source data 3. [file elife-98181-fig3-data3.zip › Figure 3-source data 3ú¿P-STAT3ú⌐.jpg]

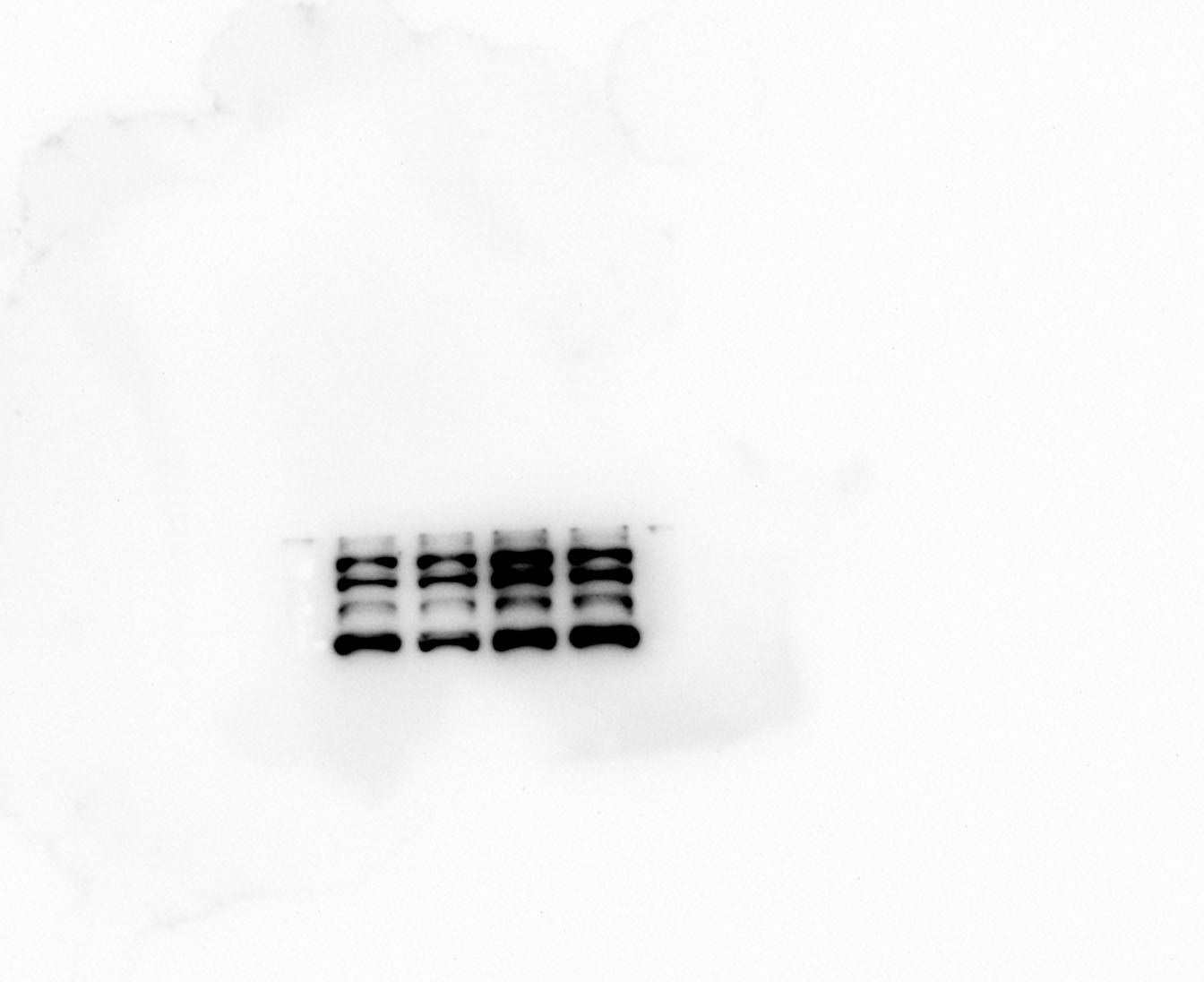

Supplement: Figure 3—source data 3. [file elife-98181-fig3-data3.zip › Figure 3-source data 3 ú¿IKKú⌐.jpg]

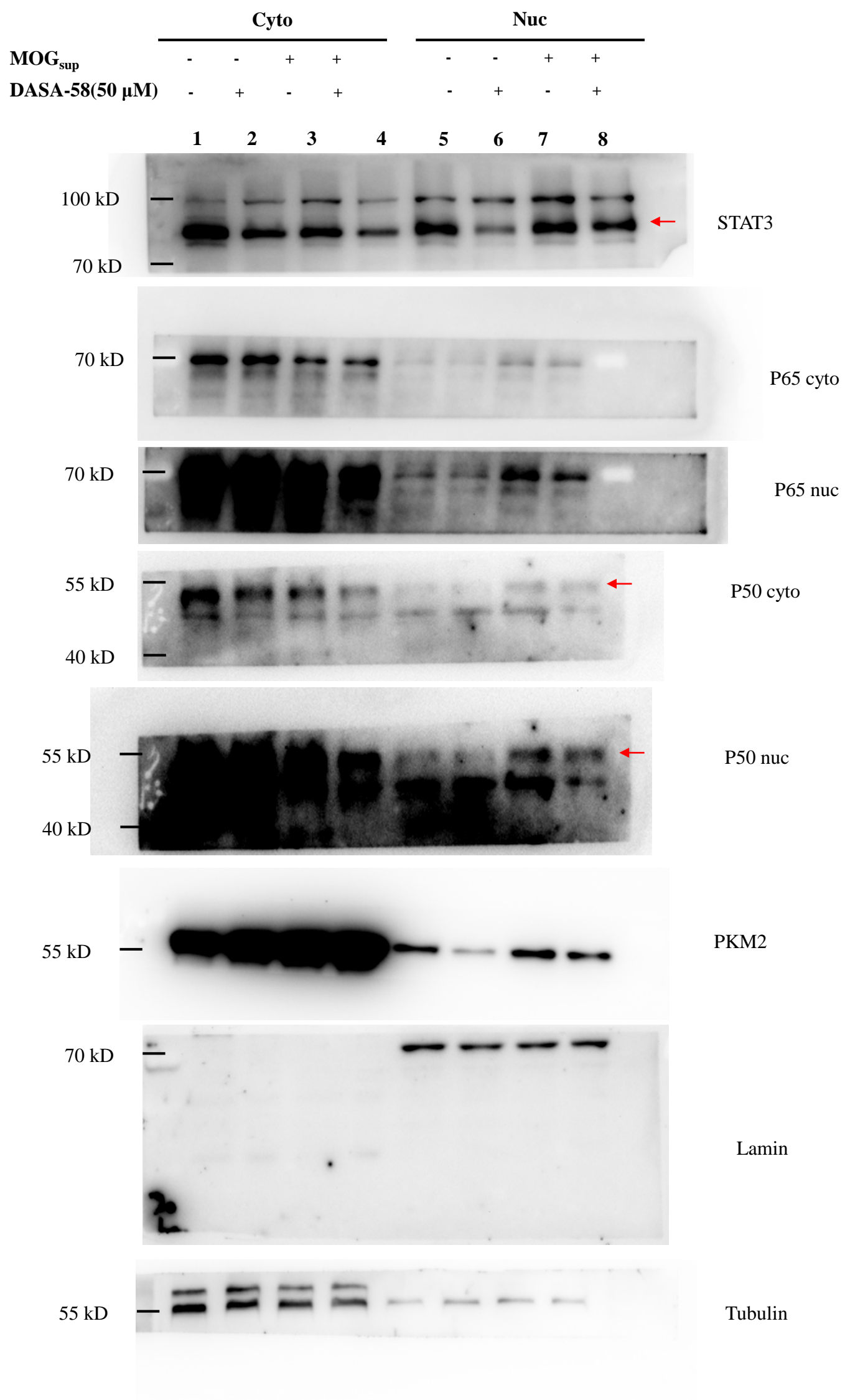

Supplement: Figure 3—source data 4. [file elife-98181-fig3-data4.pdf]

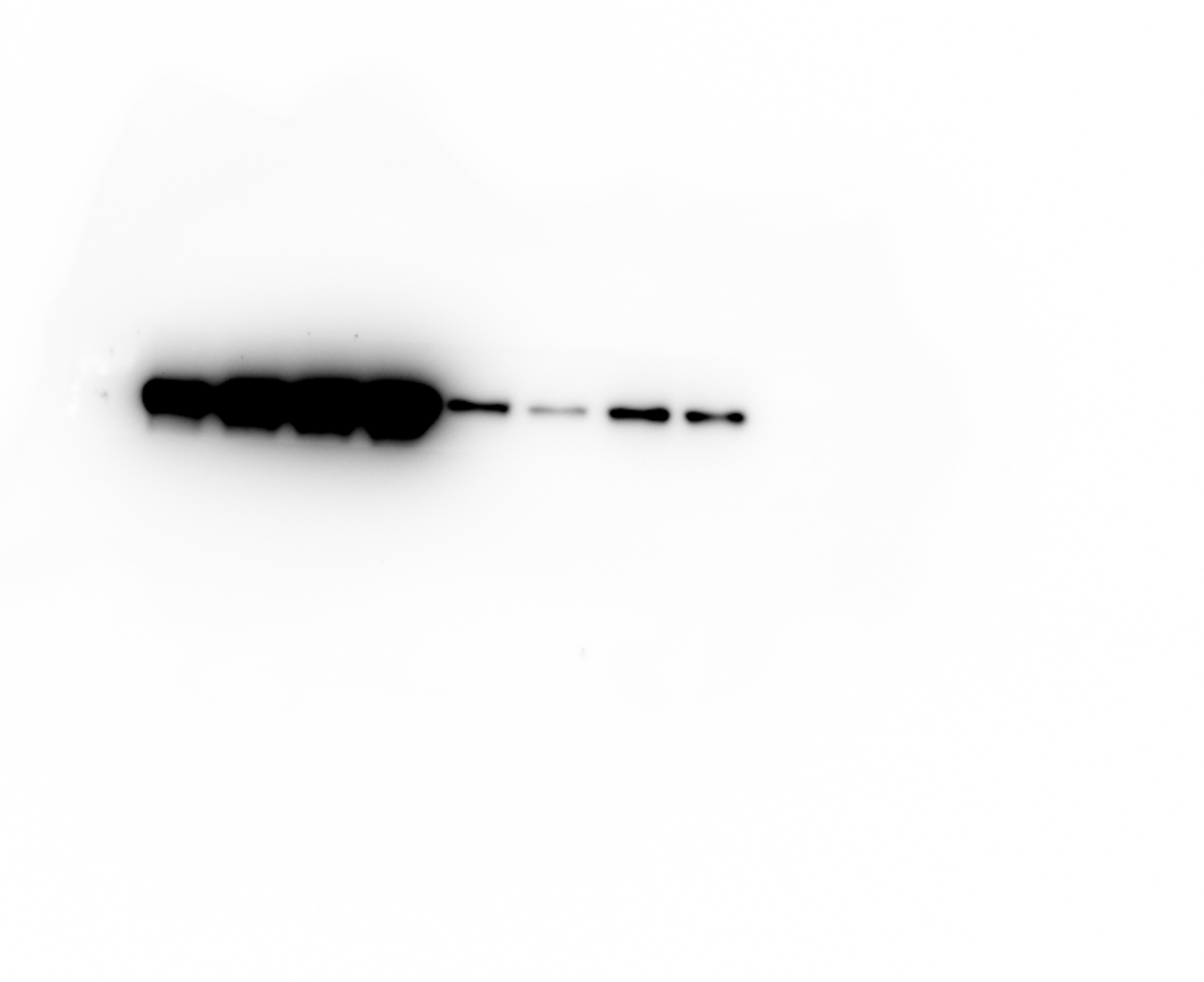

Supplement: Figure 3—source data 5. [file elife-98181-fig3-data5.zip › Figure 3-source data 5 (PKM2).tif]

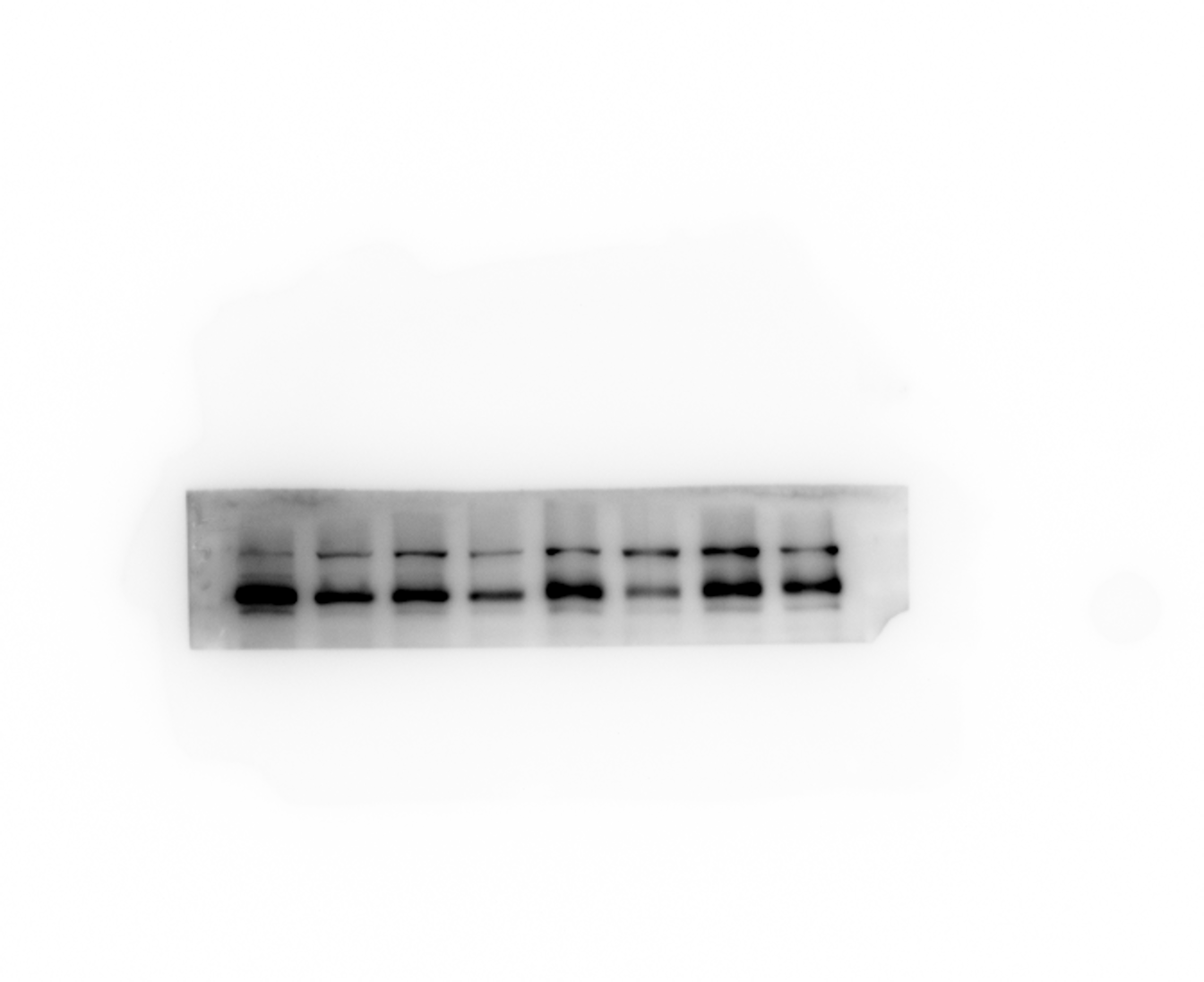

Supplement: Figure 3—source data 5. [file elife-98181-fig3-data5.zip › Figure 3-source data 5 (STAT3).tif]

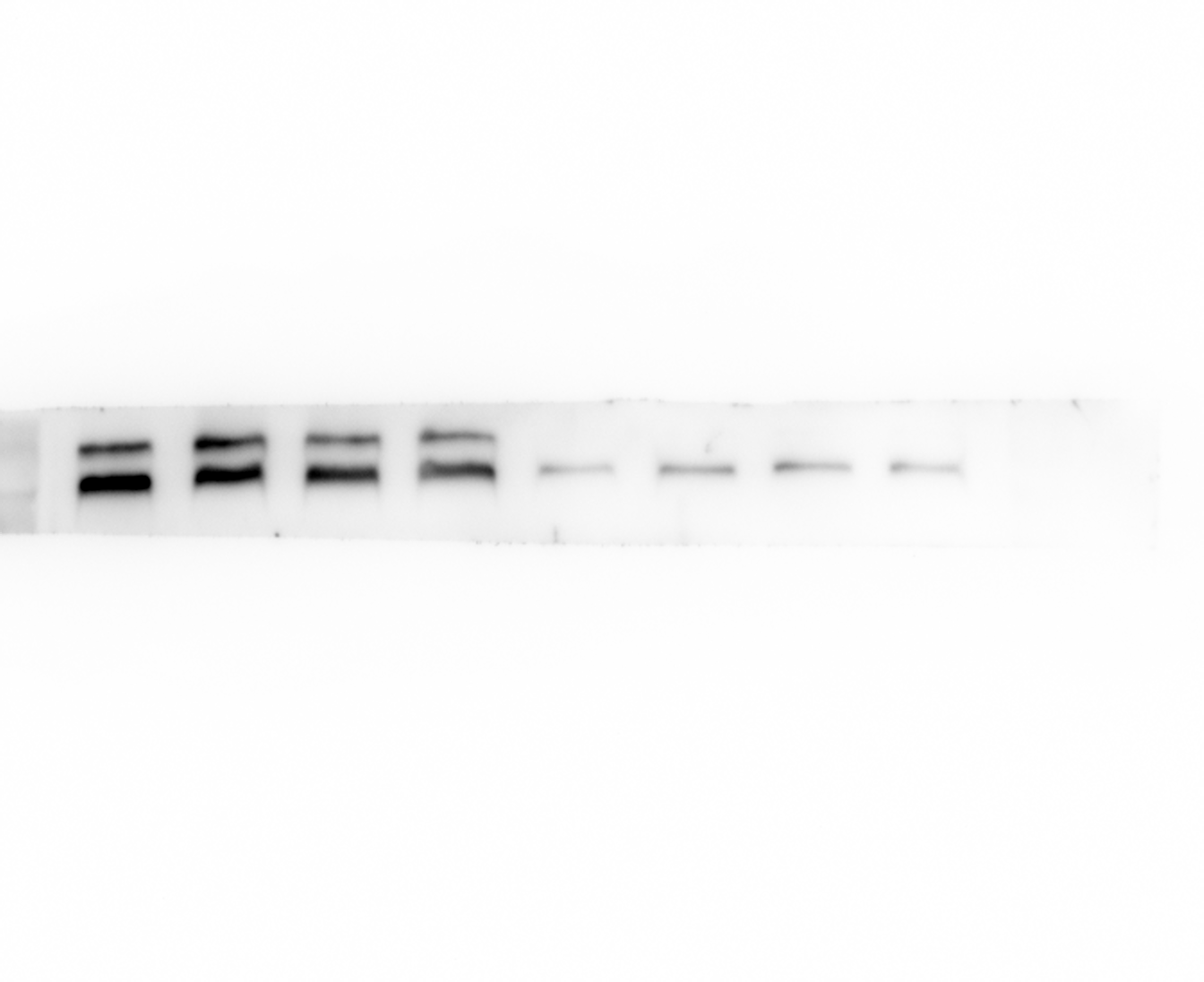

Supplement: Figure 3—source data 5. [file elife-98181-fig3-data5.zip › Figure 3-source data 5 (Tubulin).tif]

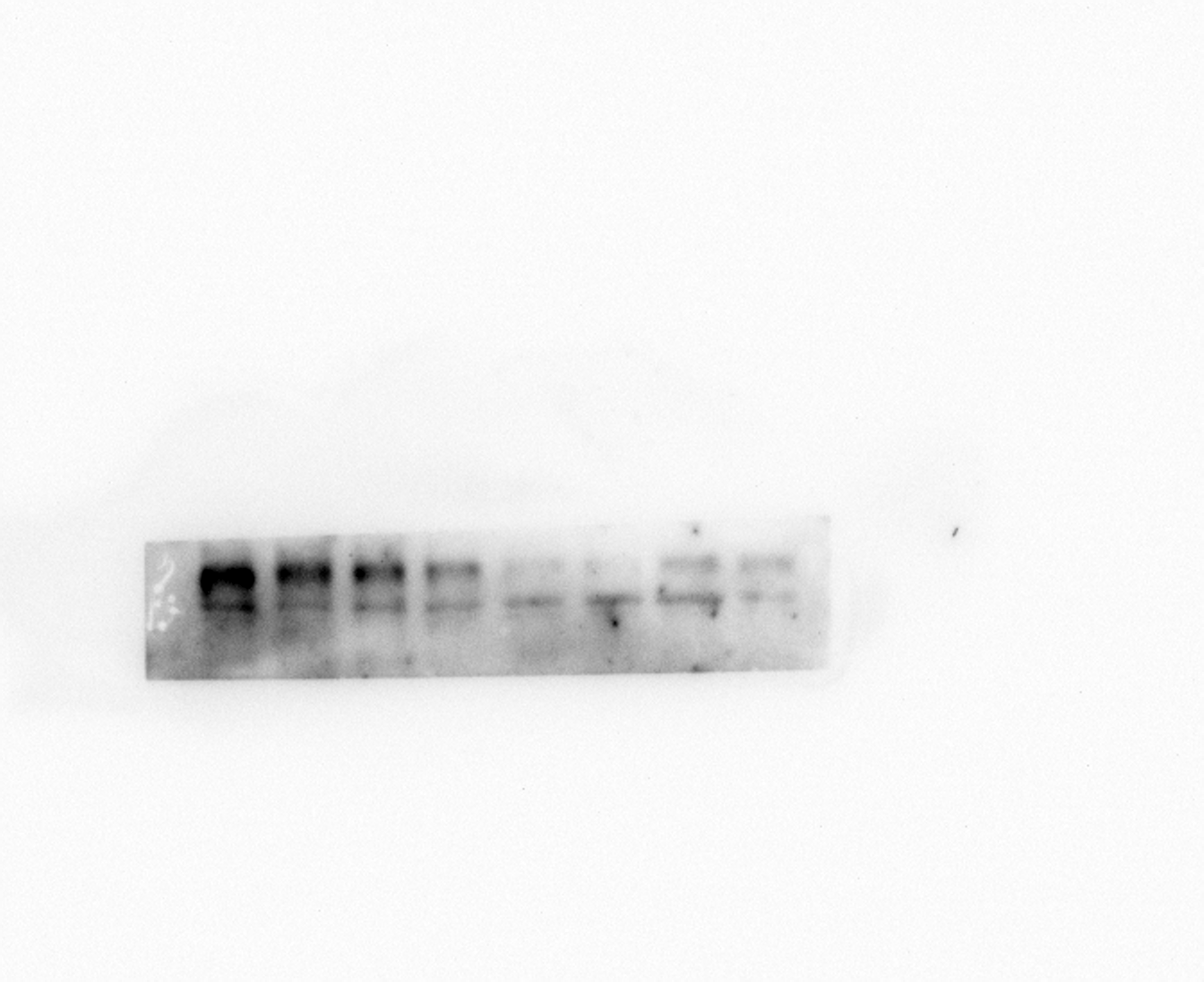

Supplement: Figure 3—source data 5. [file elife-98181-fig3-data5.zip › Figure 3-source data 5 (p50 cyto).tif]

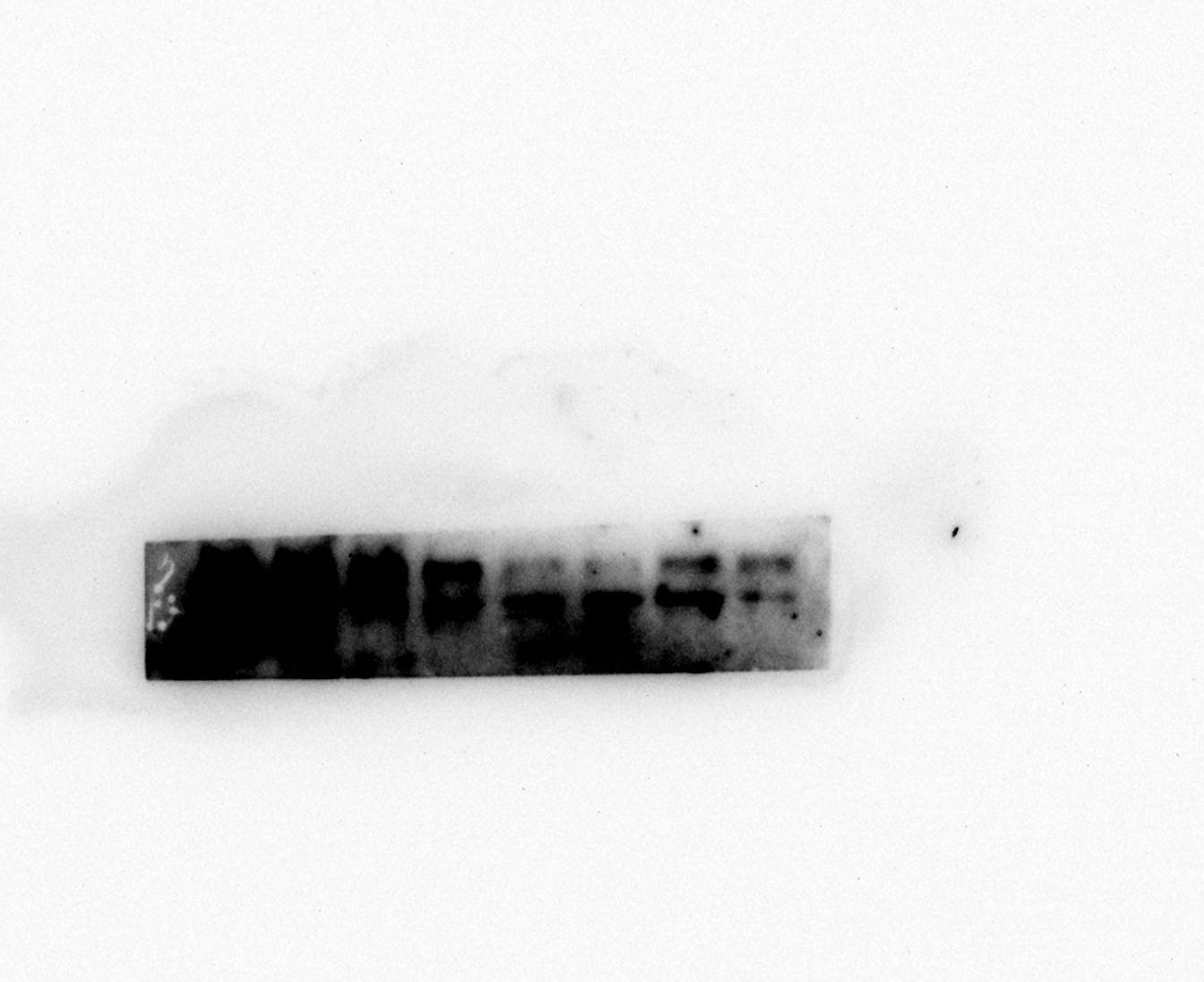

Supplement: Figure 3—source data 5. [file elife-98181-fig3-data5.zip › Figure 3-source data 5 (p50 nuc).tif]

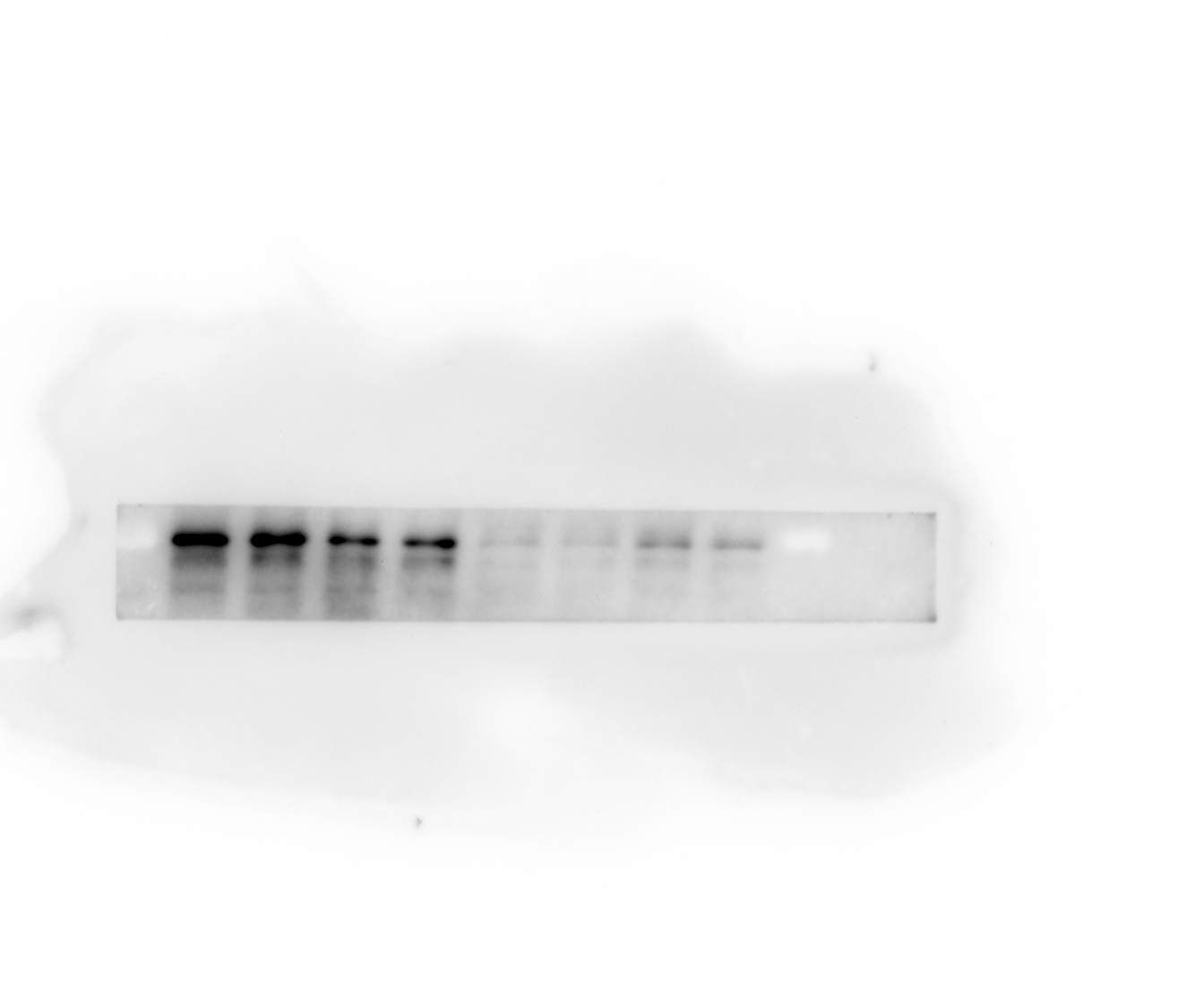

Supplement: Figure 3—source data 5. [file elife-98181-fig3-data5.zip › Figure 3-source data 5 (p65 cyto).tif]

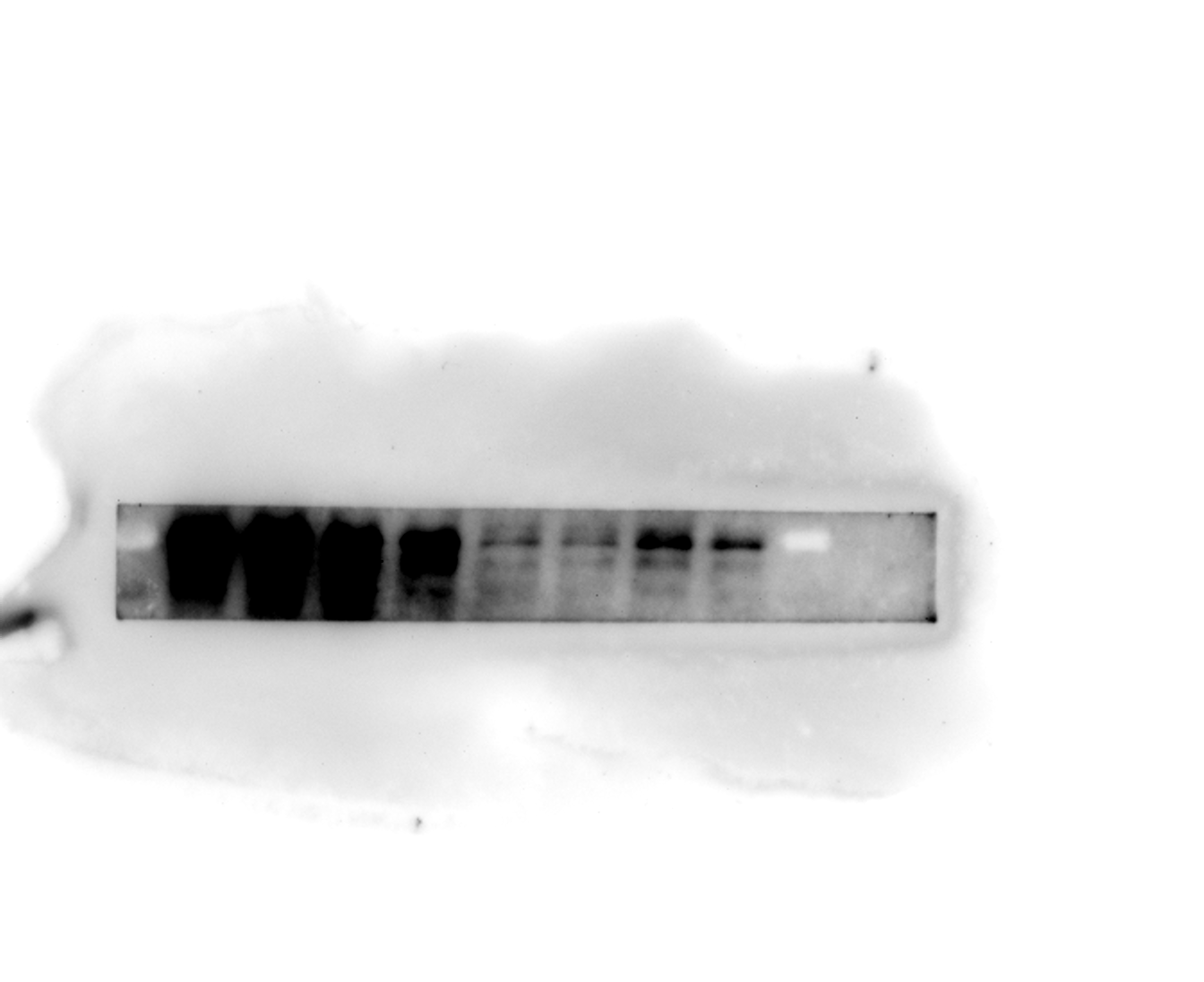

Supplement: Figure 3—source data 5. [file elife-98181-fig3-data5.zip › Figure 3-source data 5 (p65 nuc).tif]

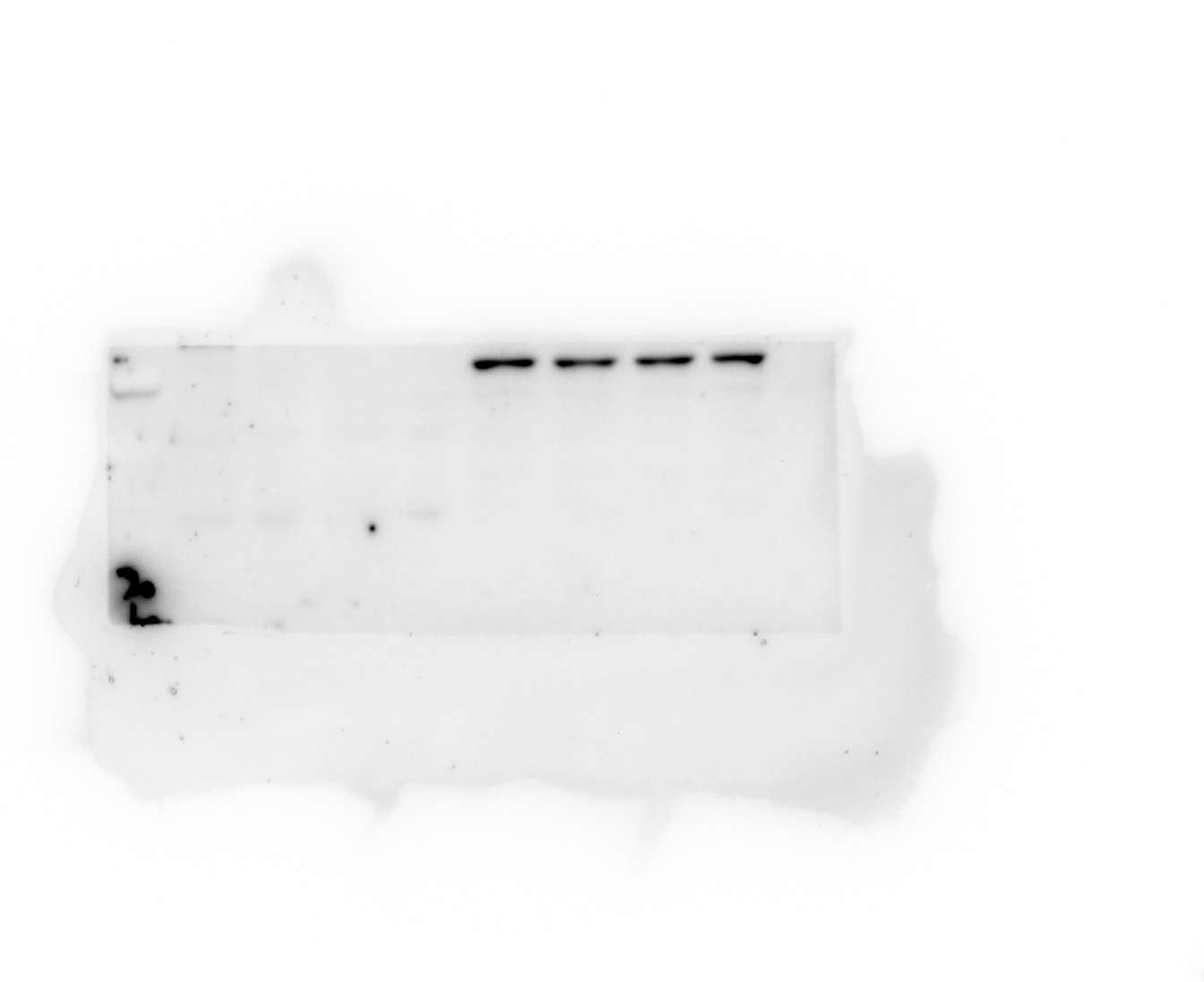

Supplement: Figure 3—source data 5. [file elife-98181-fig3-data5.zip › Figure 3-source data 5 (Lamin).tif]

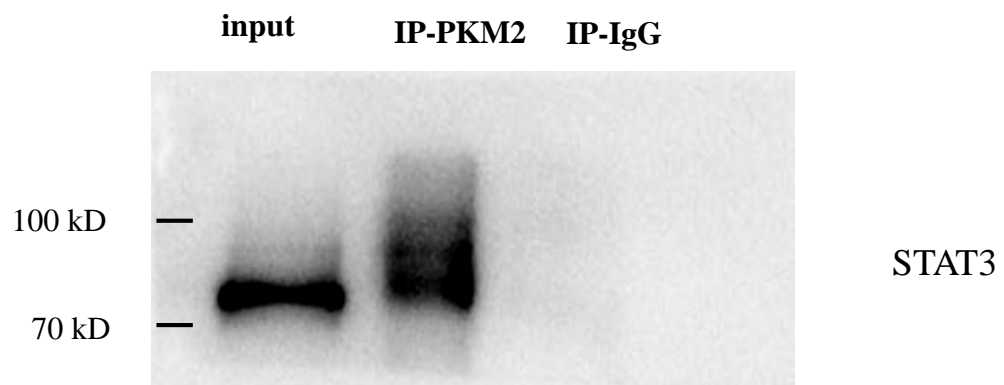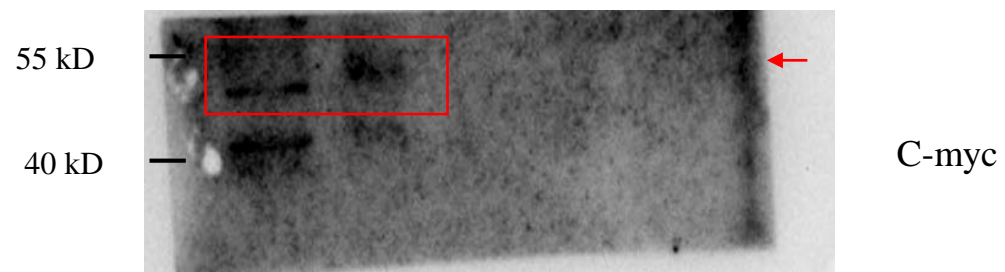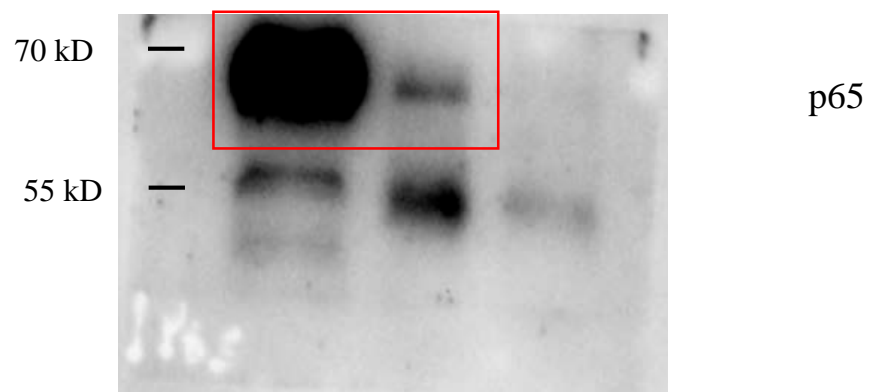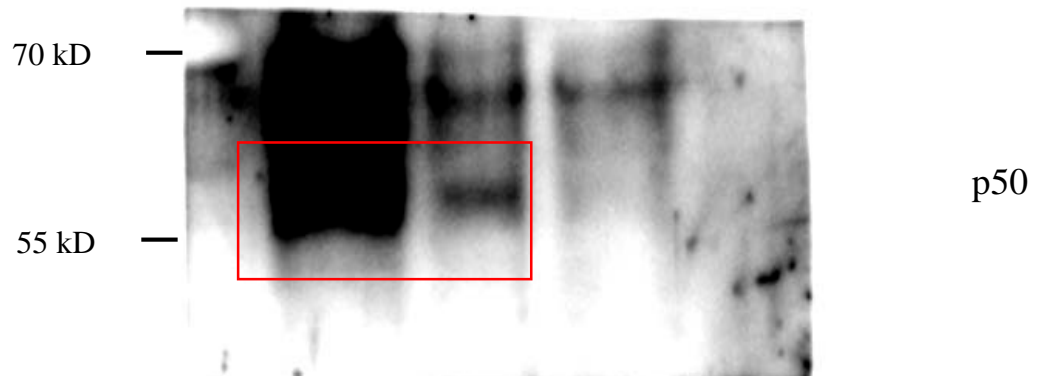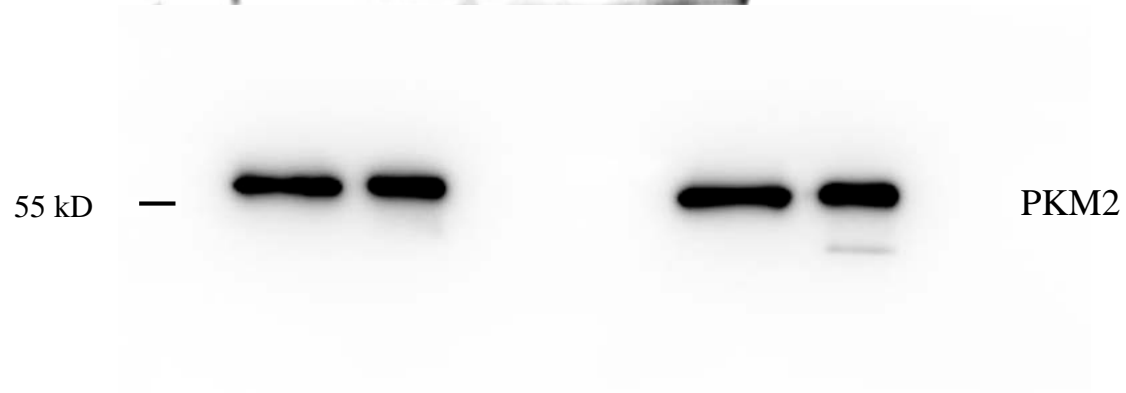

Supplement: Figure 3—source data 6. [file elife-98181-fig3-data6.pdf]

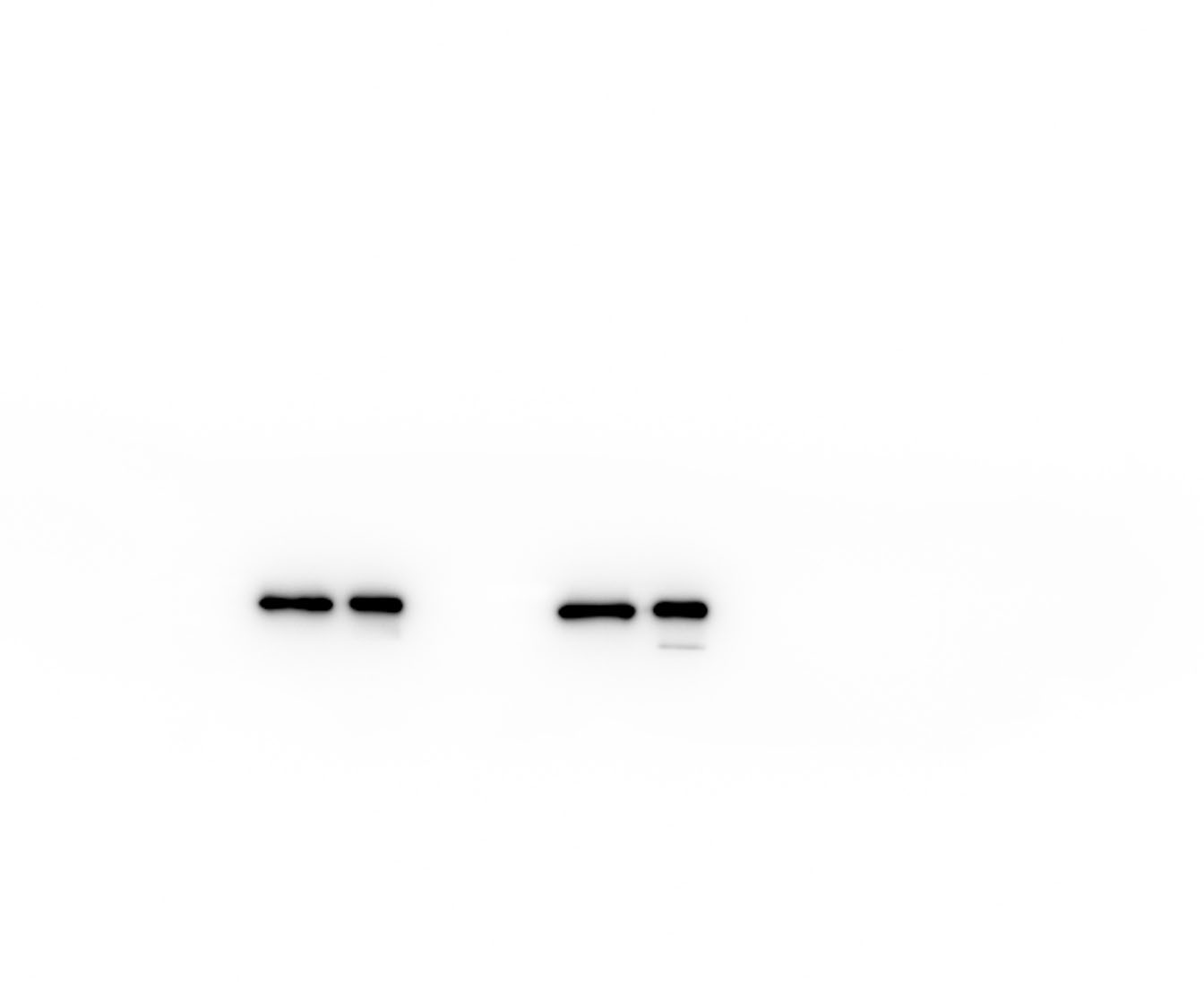

Supplement: Figure 3—source data 7. [file elife-98181-fig3-data7.zip › Figure 3-source data 7(PKM2).jpg]

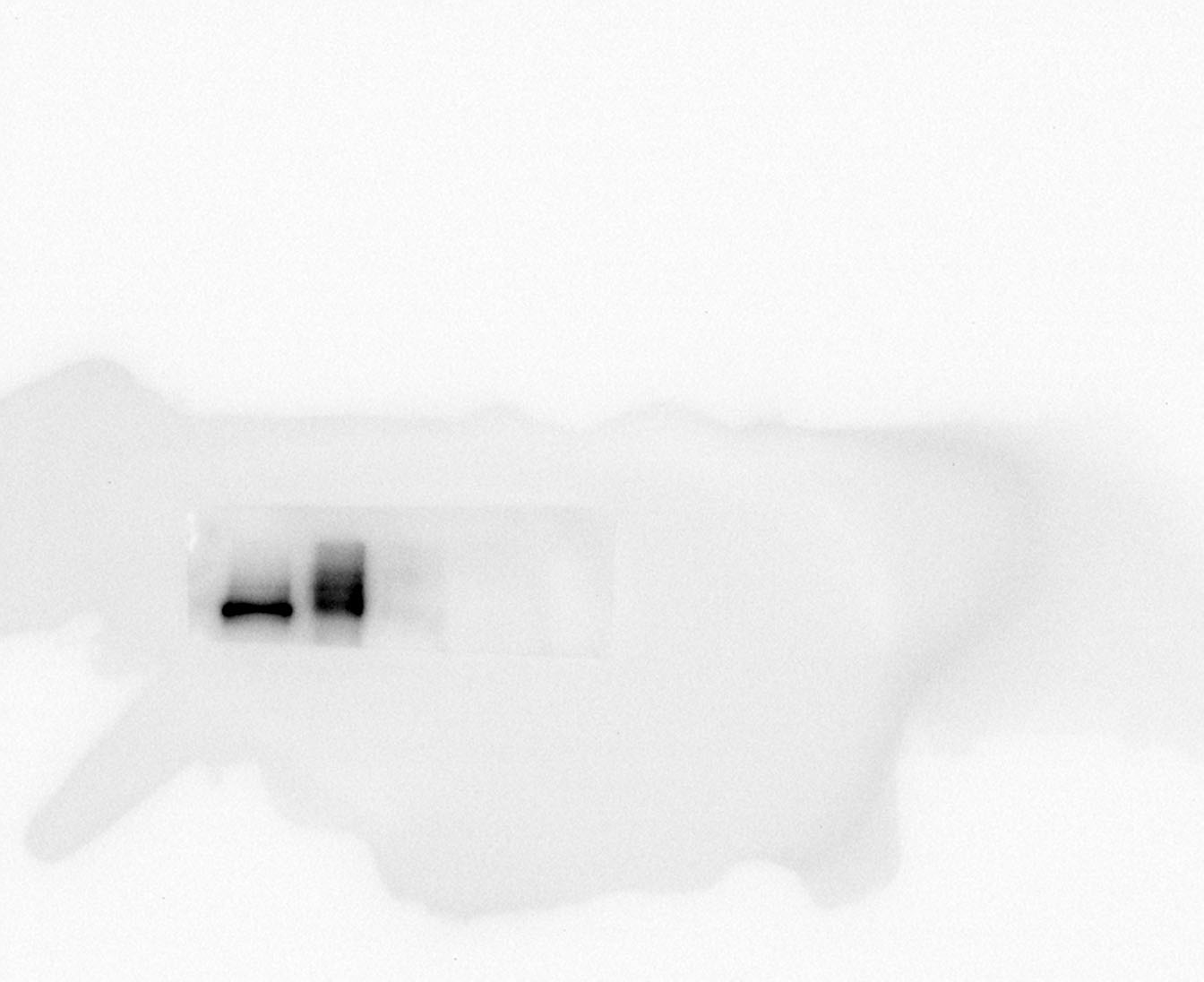

Supplement: Figure 3—source data 7. [file elife-98181-fig3-data7.zip › Figure 3-source data 7(STAT3).jpg]

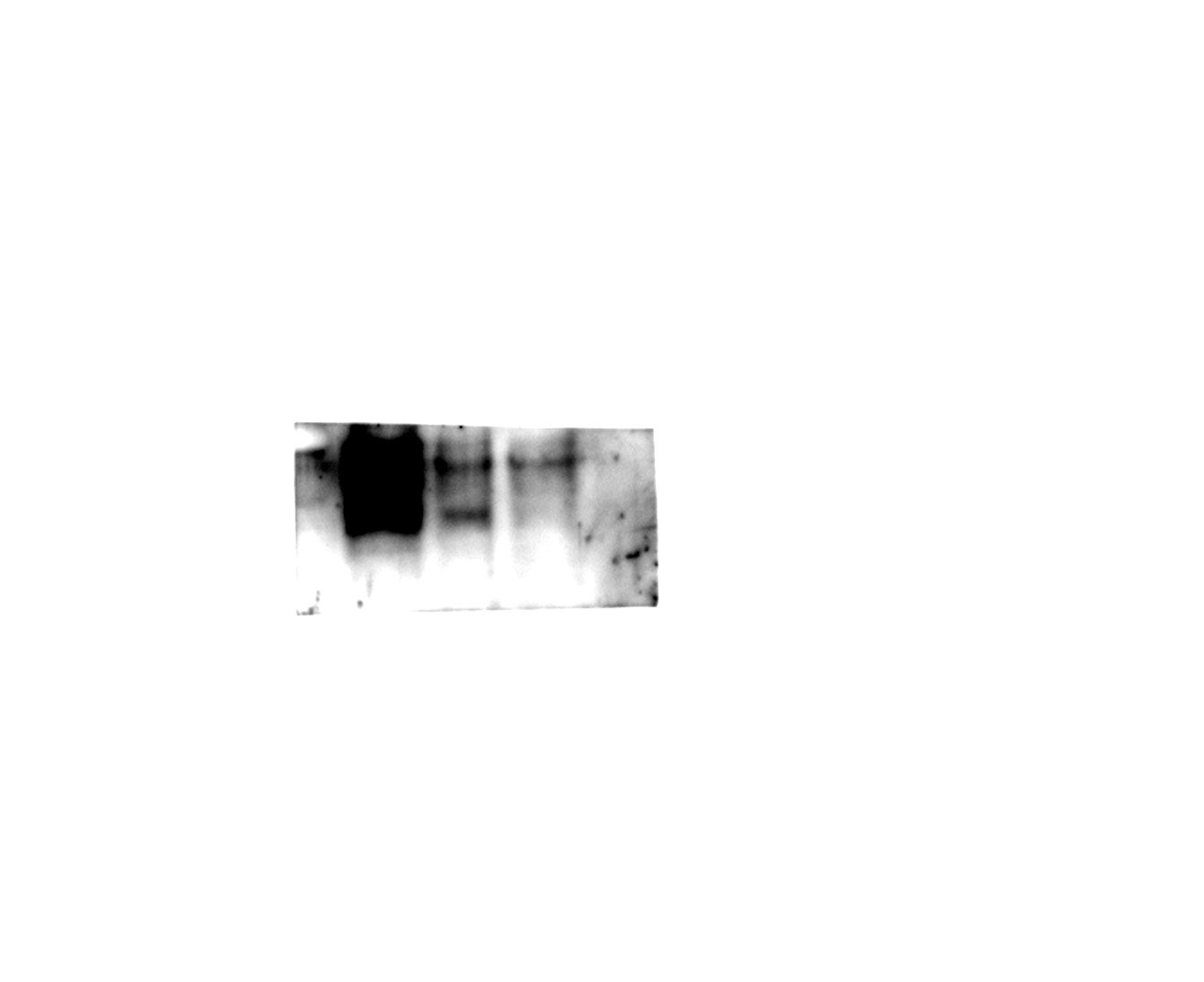

Supplement: Figure 3—source data 7. [file elife-98181-fig3-data7.zip › Figure 3-source data 7(p50).jpg]

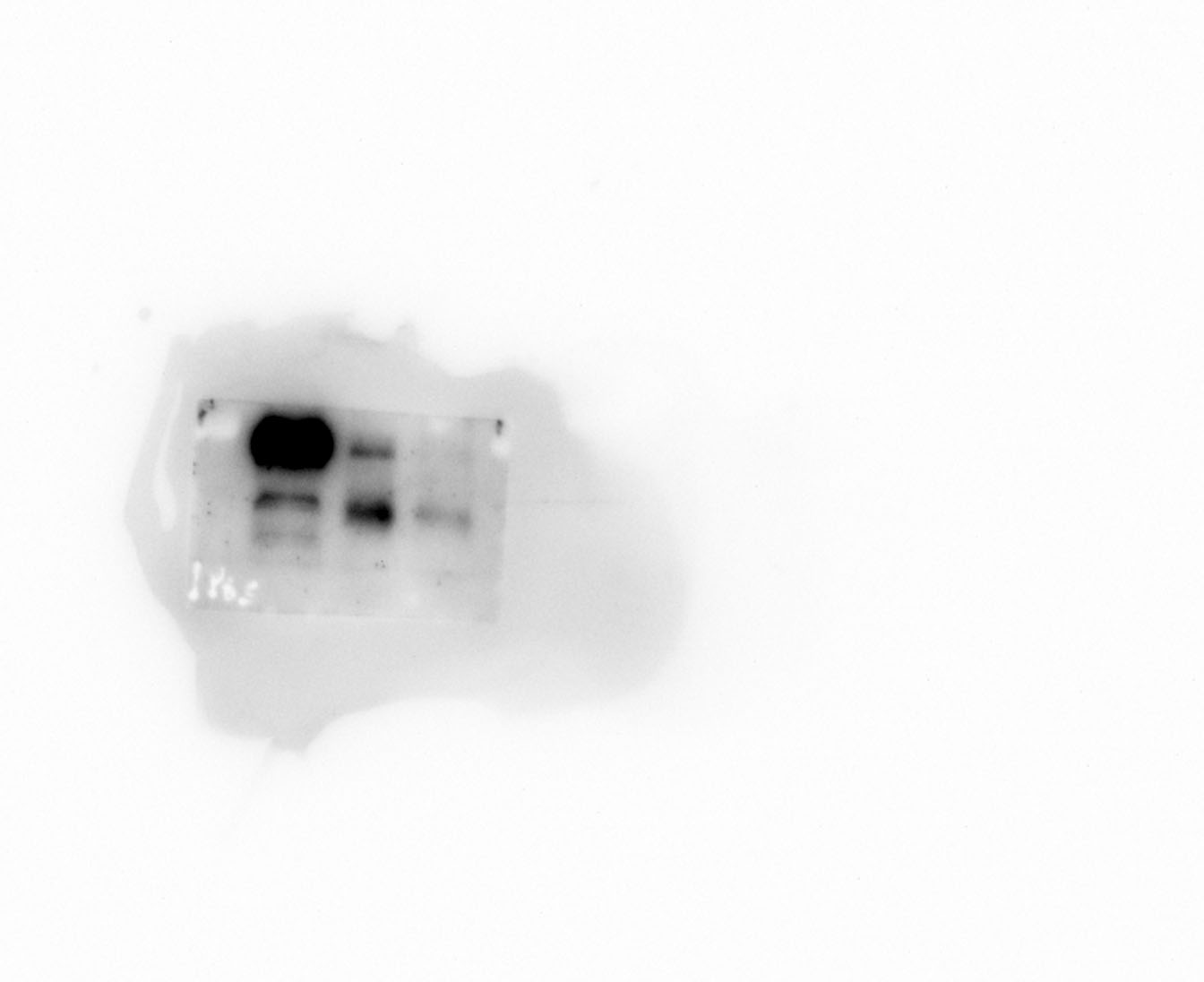

Supplement: Figure 3—source data 7. [file elife-98181-fig3-data7.zip › Figure 3-source data 7(p65).jpg]

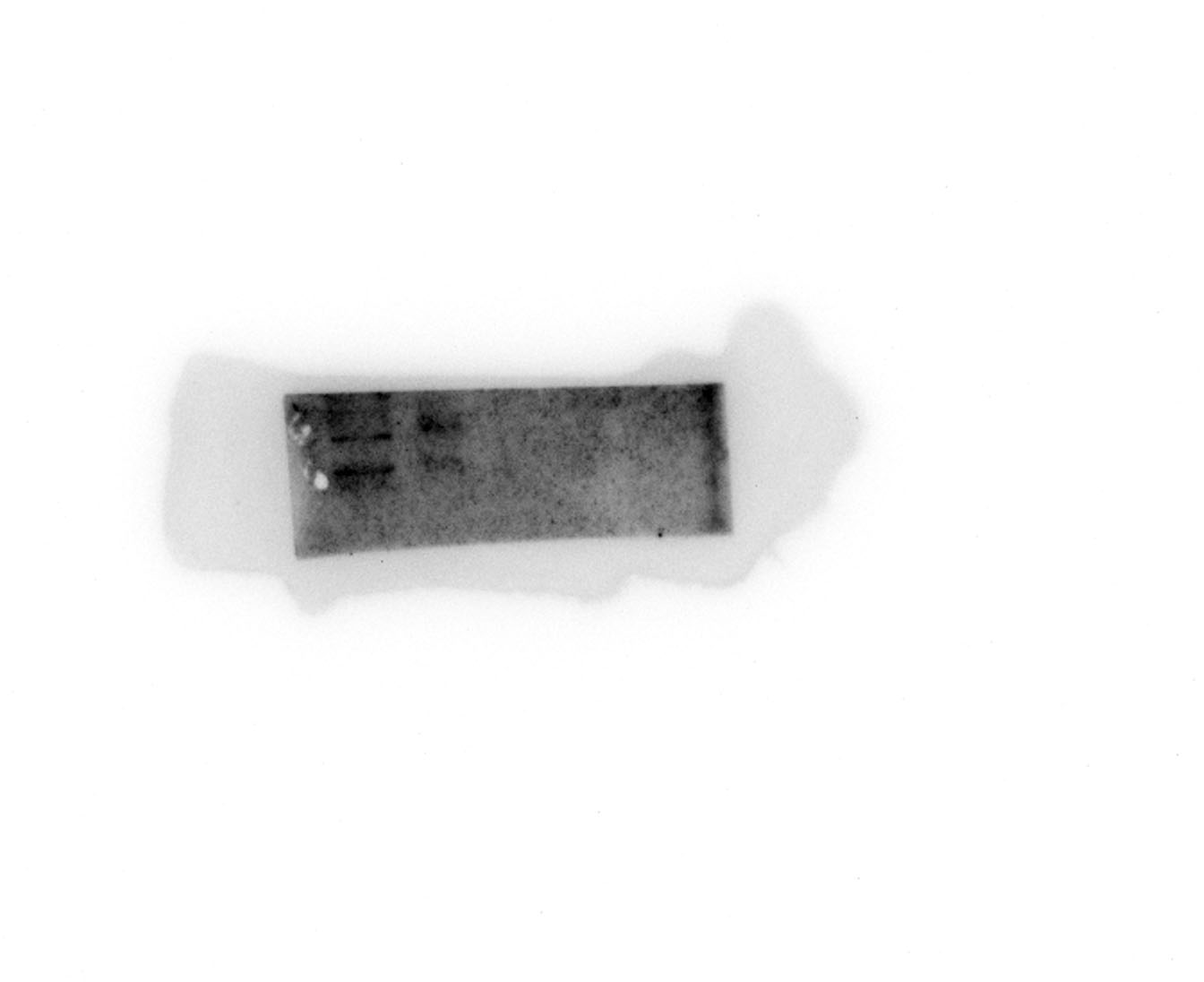

Supplement: Figure 3—source data 7. [file elife-98181-fig3-data7.zip › Figure 3-source data 7 (c-myc).jpg]

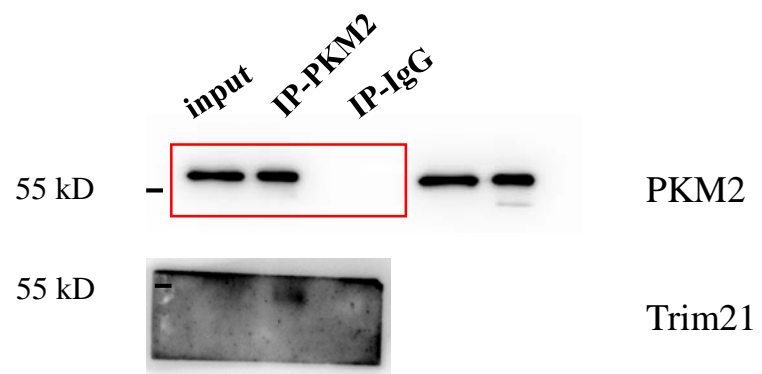

Supplement: Figure 4—source data 1. [file elife-98181-fig4-data1.pdf]

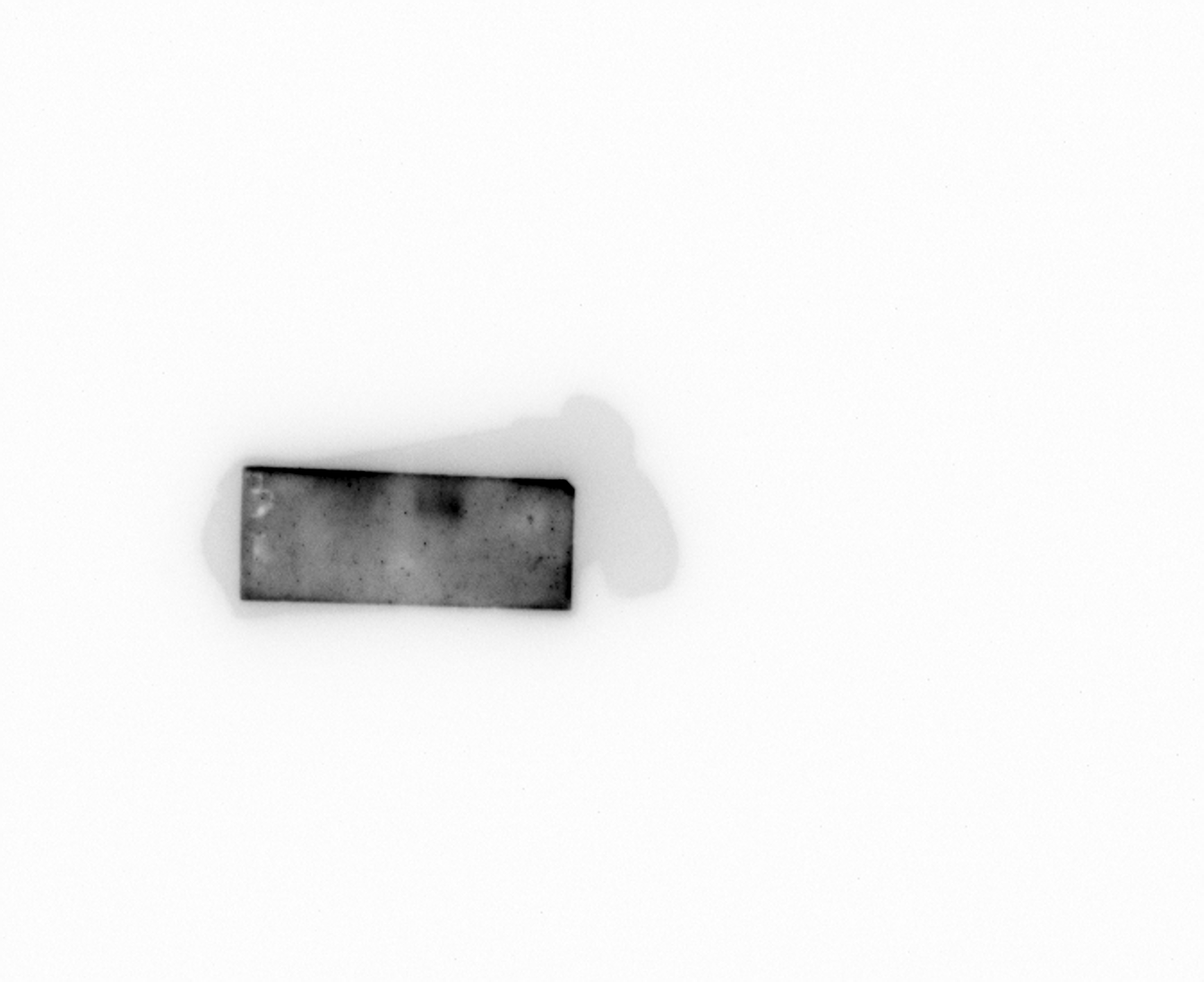

Supplement: Figure 4—source data 2. [file elife-98181-fig4-data2.zip › Figure 4-source data 2 (TRIM21).tif]

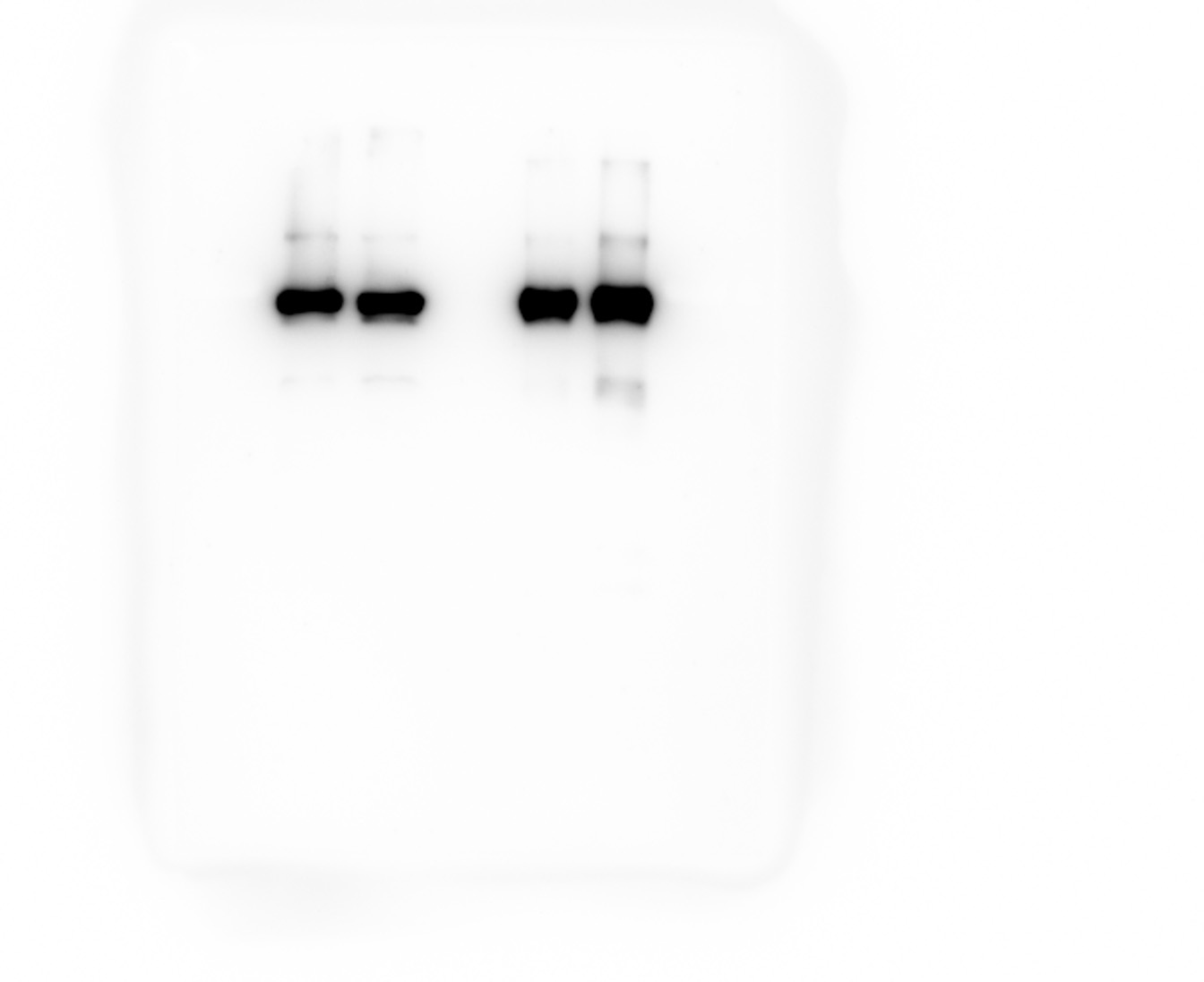

Supplement: Figure 4—source data 4. [file elife-98181-fig4-data4.zip › Figure 4-source data 4 (Flag).jpg]

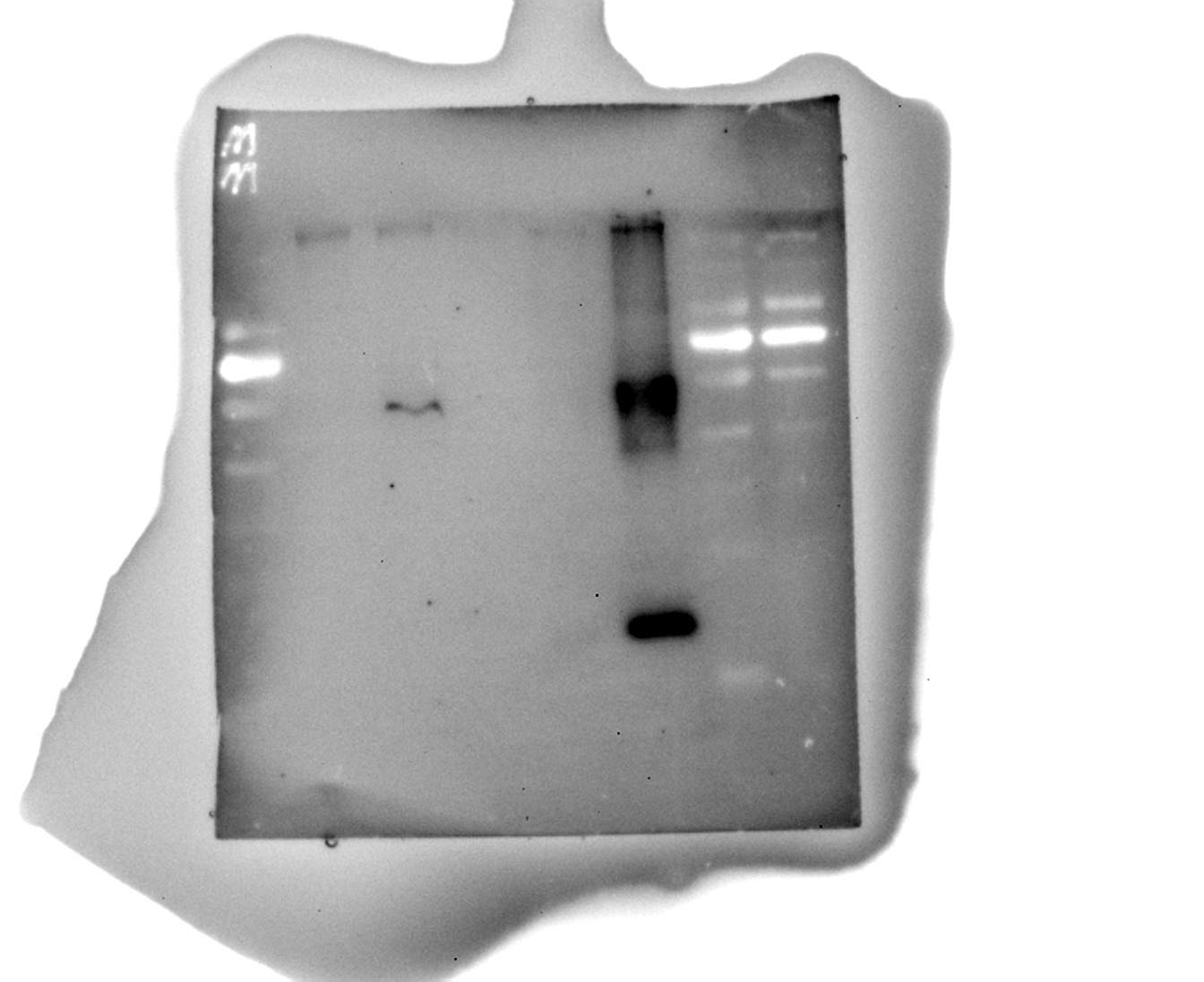

Supplement: Figure 4—source data 4. [file elife-98181-fig4-data4.zip › Figure 4-source data 4 (Myc).tif]

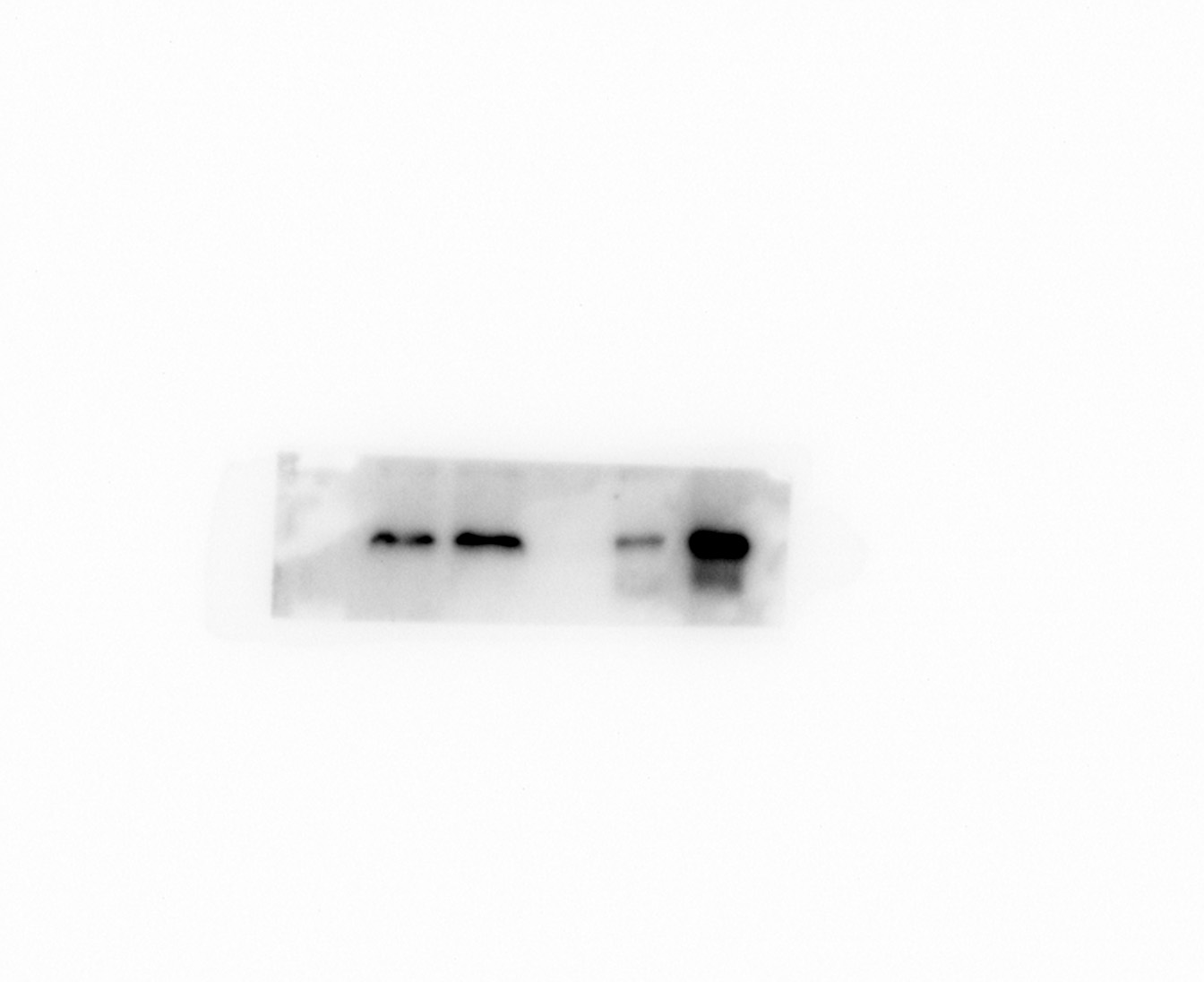

Supplement: Figure 4—source data 4. [file elife-98181-fig4-data4.zip › Figure 4-source data 4 (TRIM21).jpg]

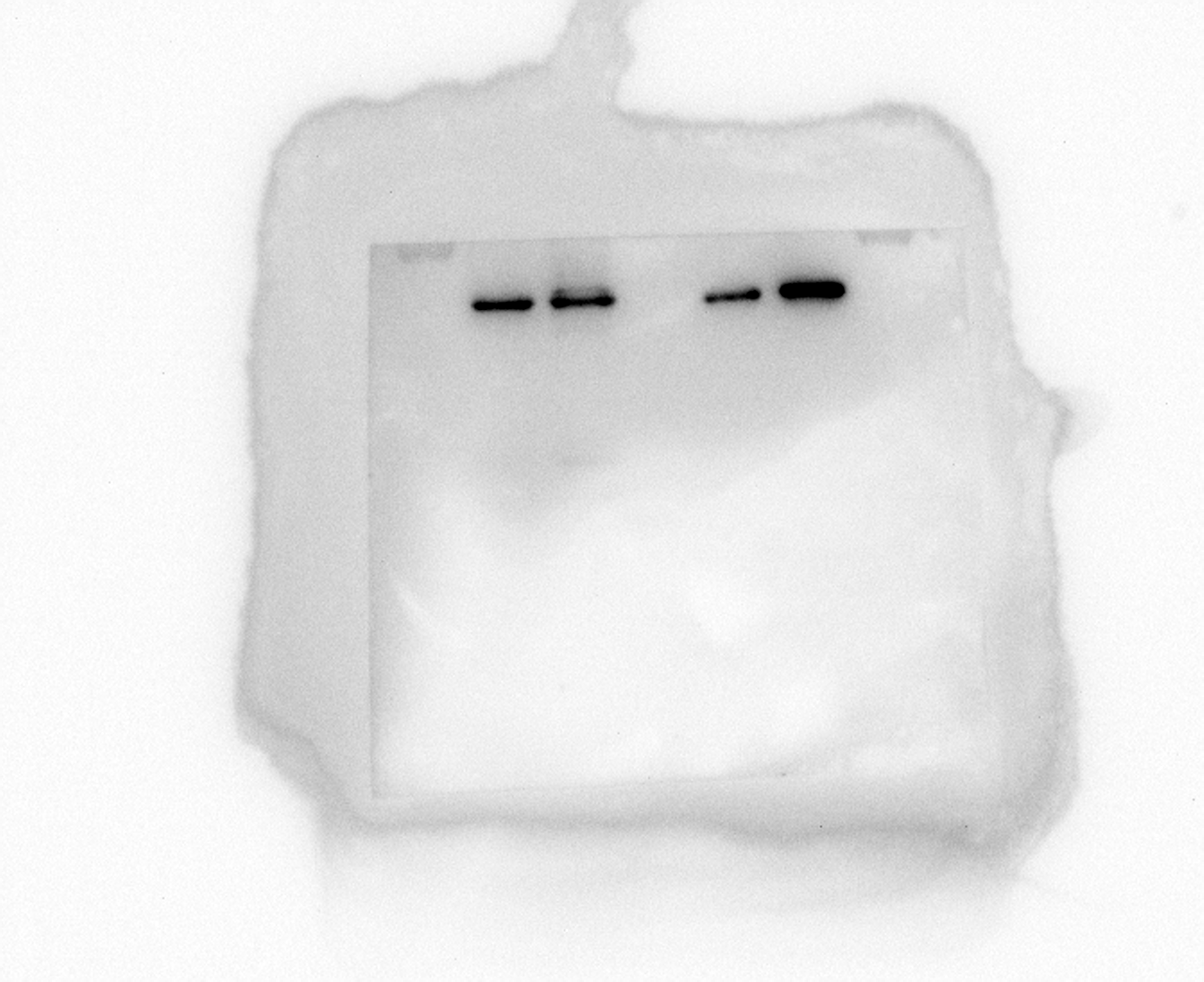

Supplement: Figure 4—source data 4. [file elife-98181-fig4-data4.zip › Figure 4-source data 4 (Actin).tif]

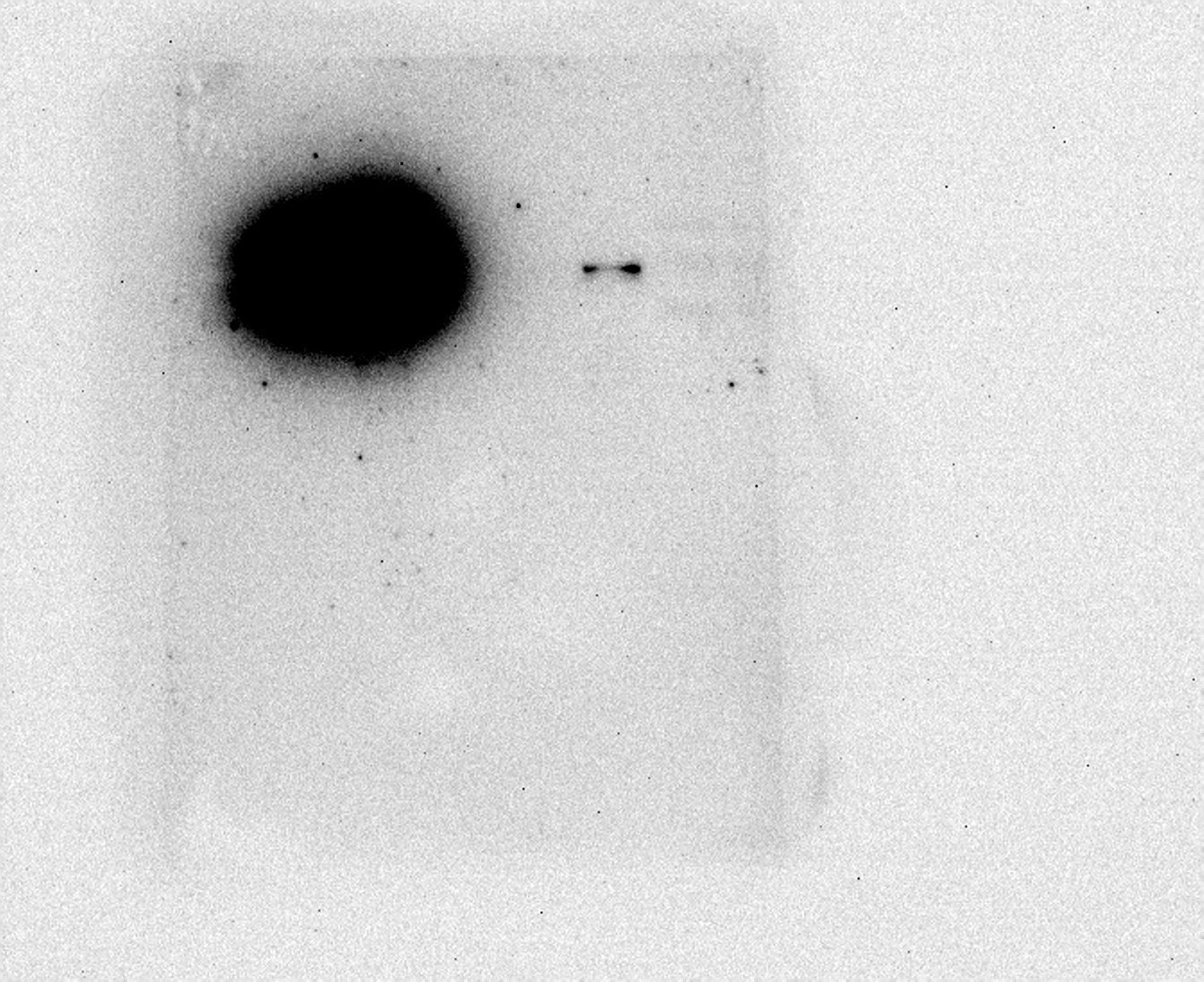

Supplement: Figure 4—source data 6. [file elife-98181-fig4-data6.zip › Figure 4-source data 6 (Flag IP).tif]

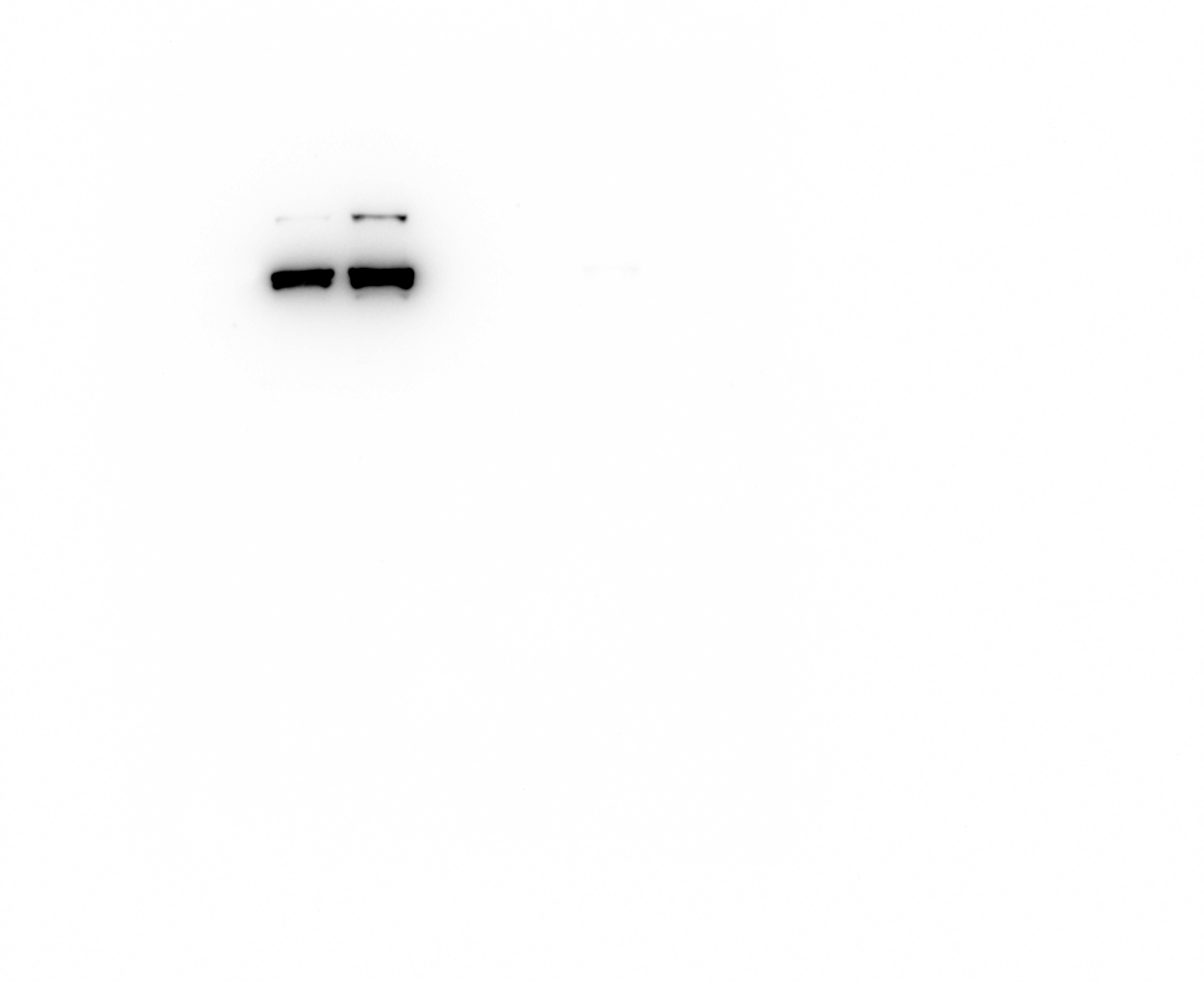

Supplement: Figure 4—source data 6. [file elife-98181-fig4-data6.zip › Figure 4-source data 6 (Flag input).tif]

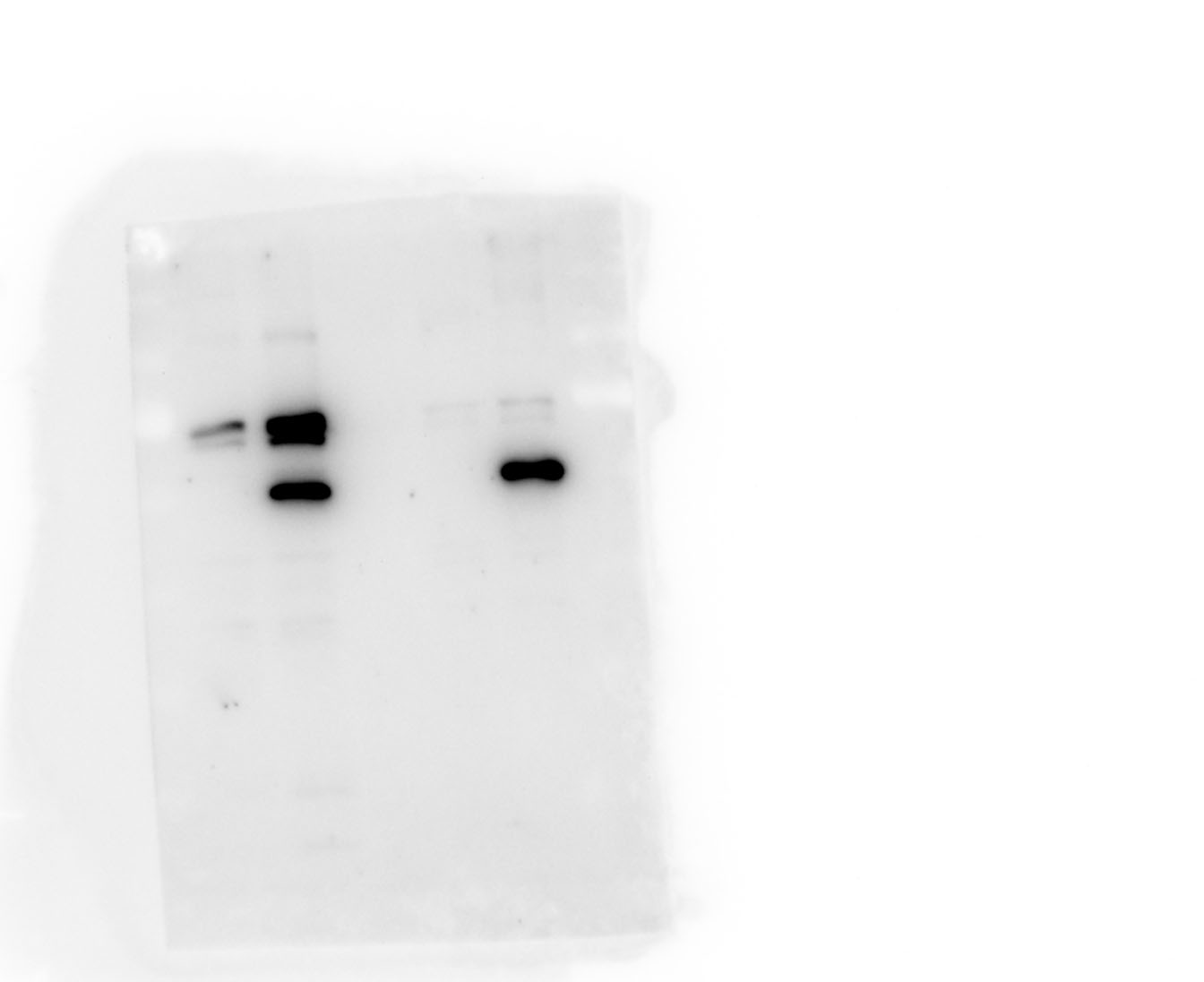

Supplement: Figure 4—source data 6. [file elife-98181-fig4-data6.zip › Figure 4-source data 6 (Myc).jpg]

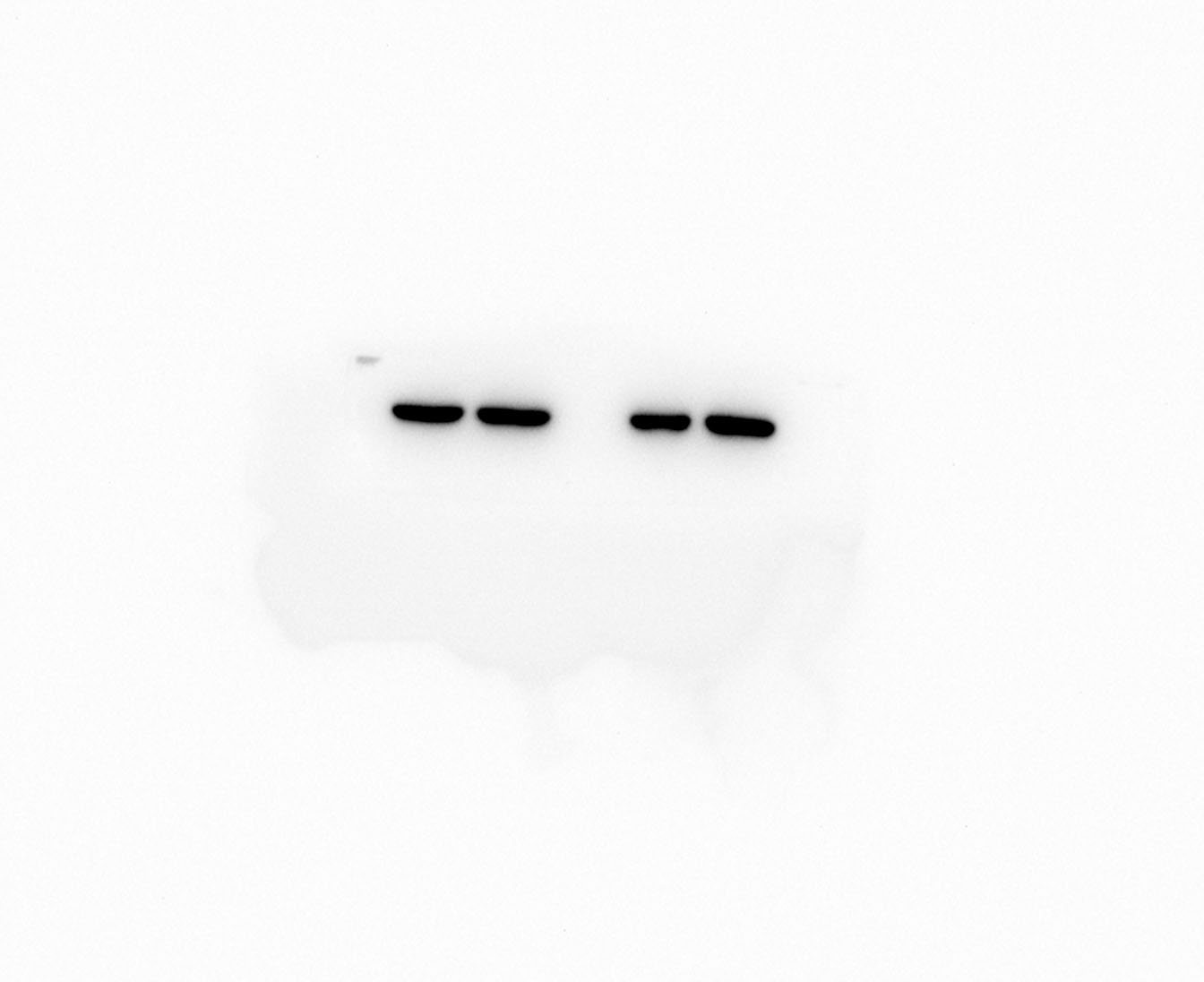

Supplement: Figure 4—source data 6. [file elife-98181-fig4-data6.zip › Figure 4-source data 6 (Actin).jpg]

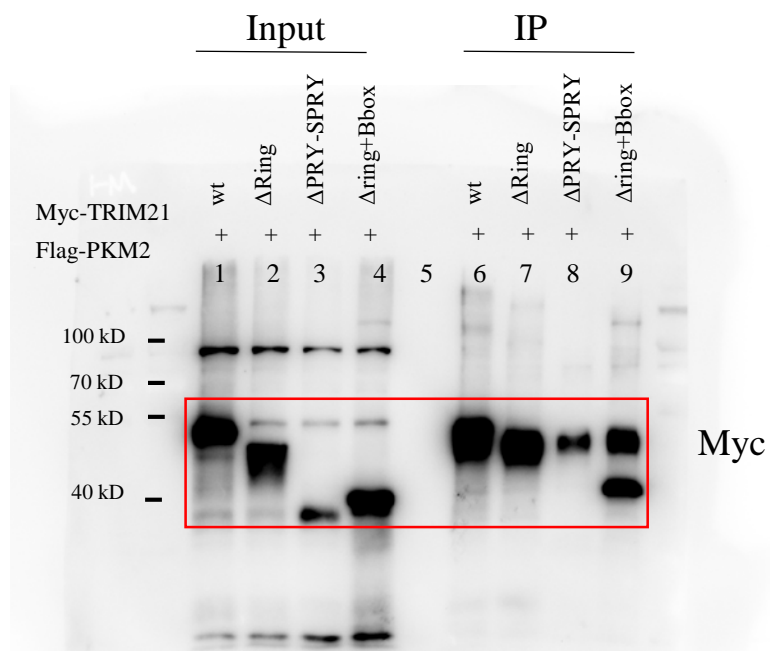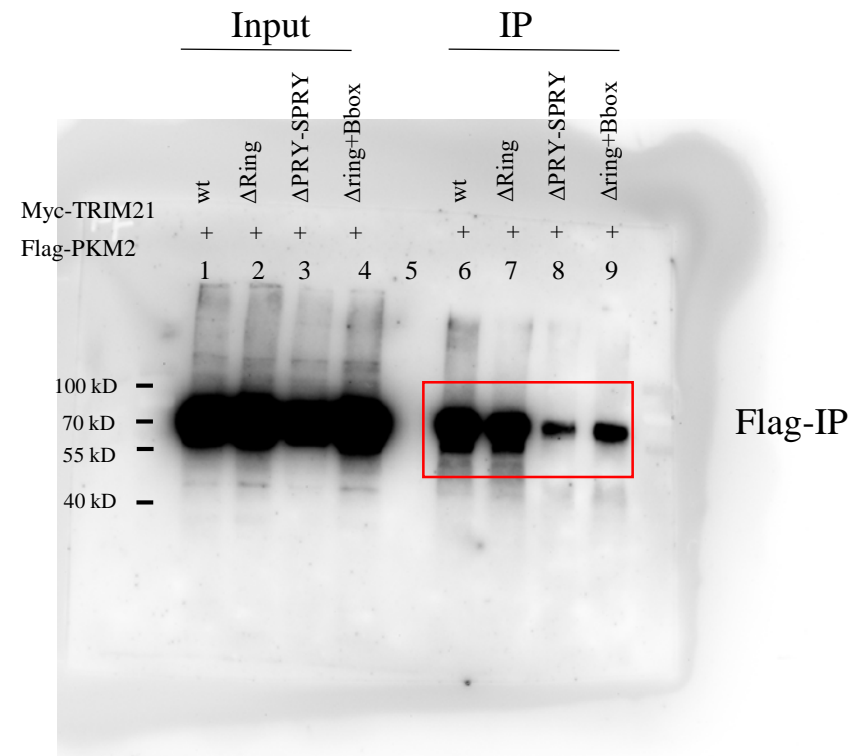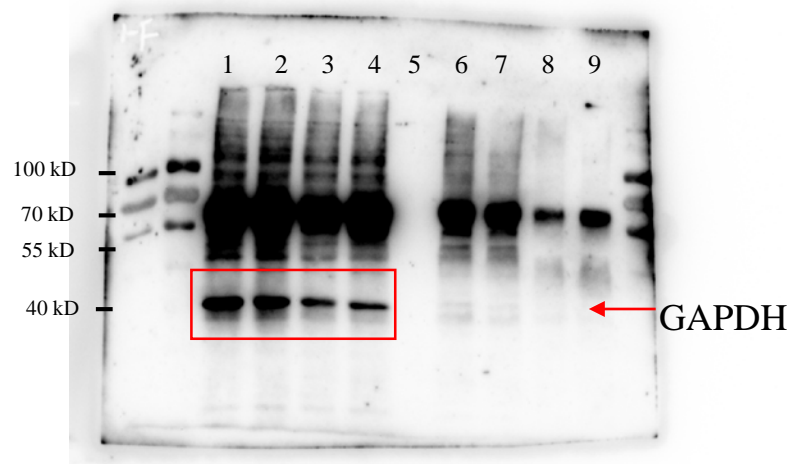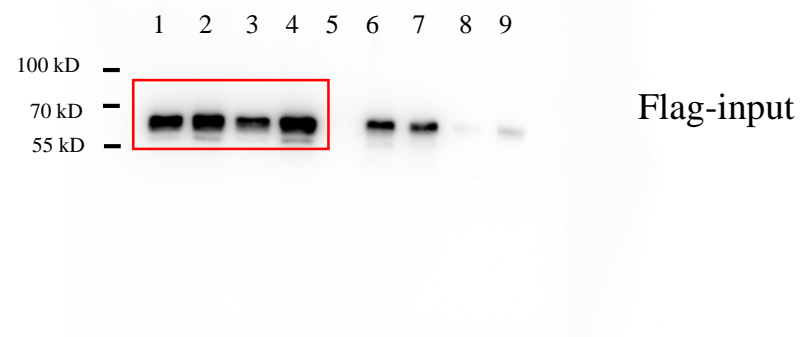

Supplement: Figure 4—source data 7. [file elife-98181-fig4-data7.pdf]

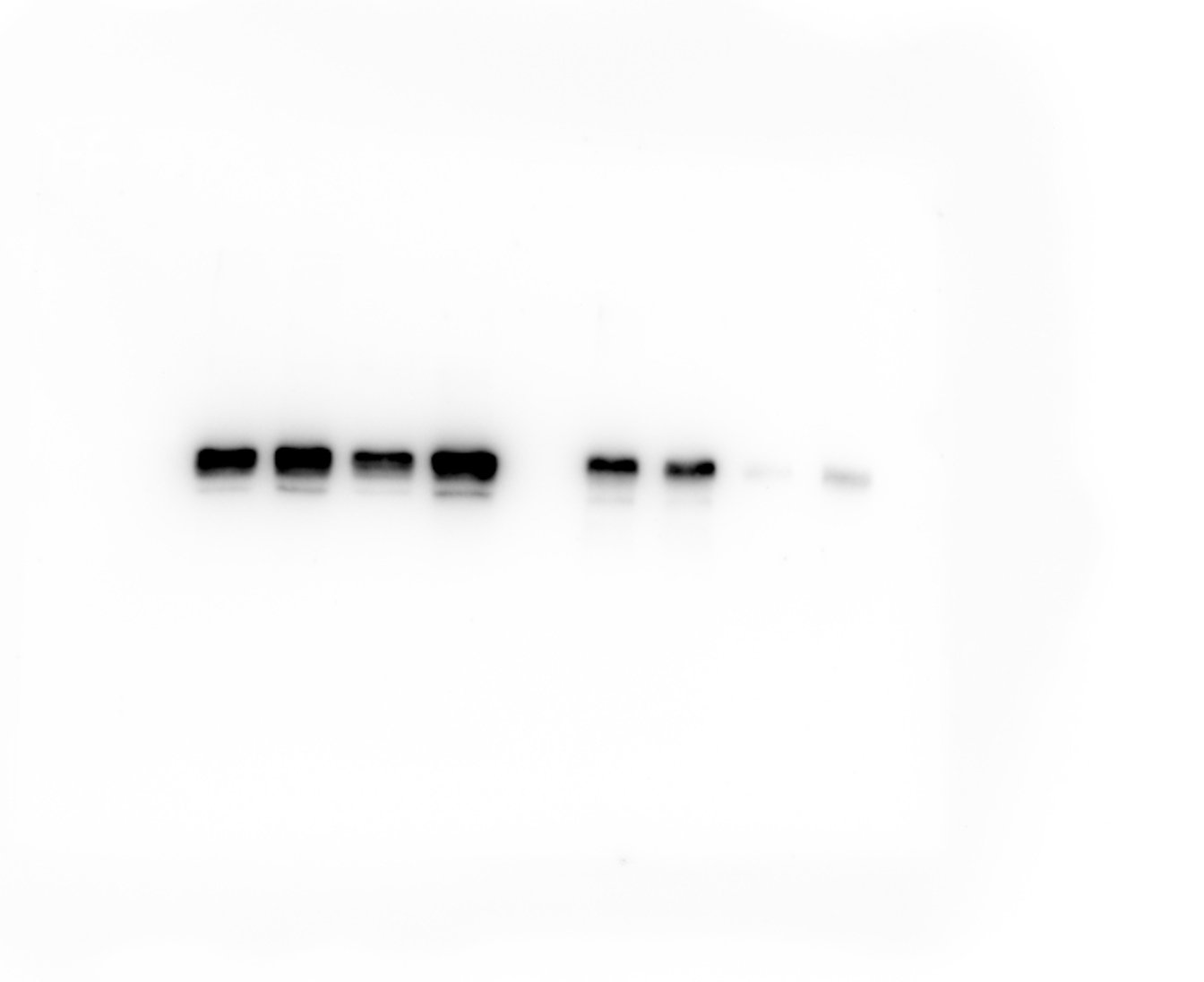

Supplement: Figure 4—source data 8. [file elife-98181-fig4-data8.zip › Figure 4-source data 8 (Flag input).tif]

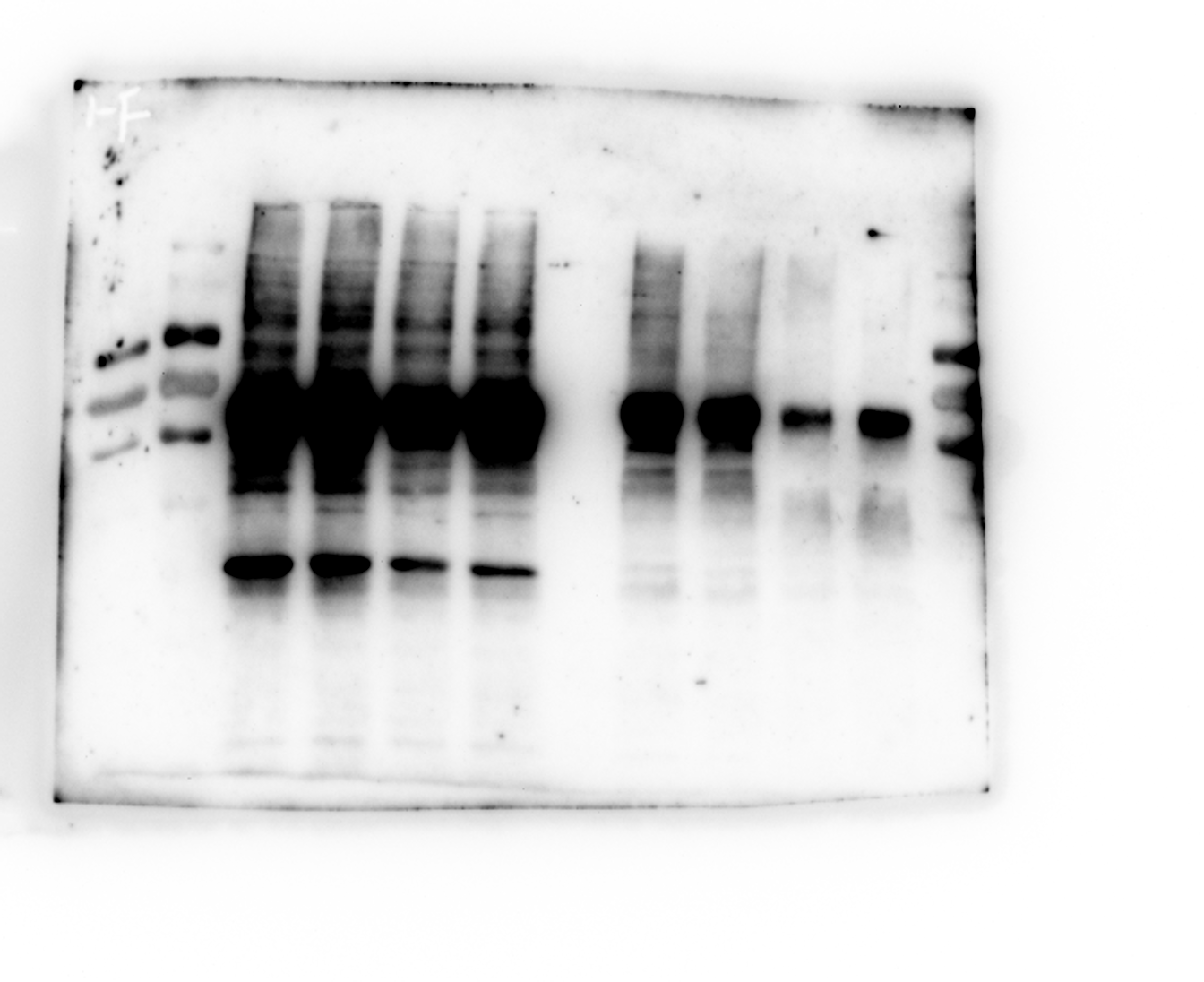

Supplement: Figure 4—source data 8. [file elife-98181-fig4-data8.zip › Figure 4-source data 8 (GAPDH).tif]

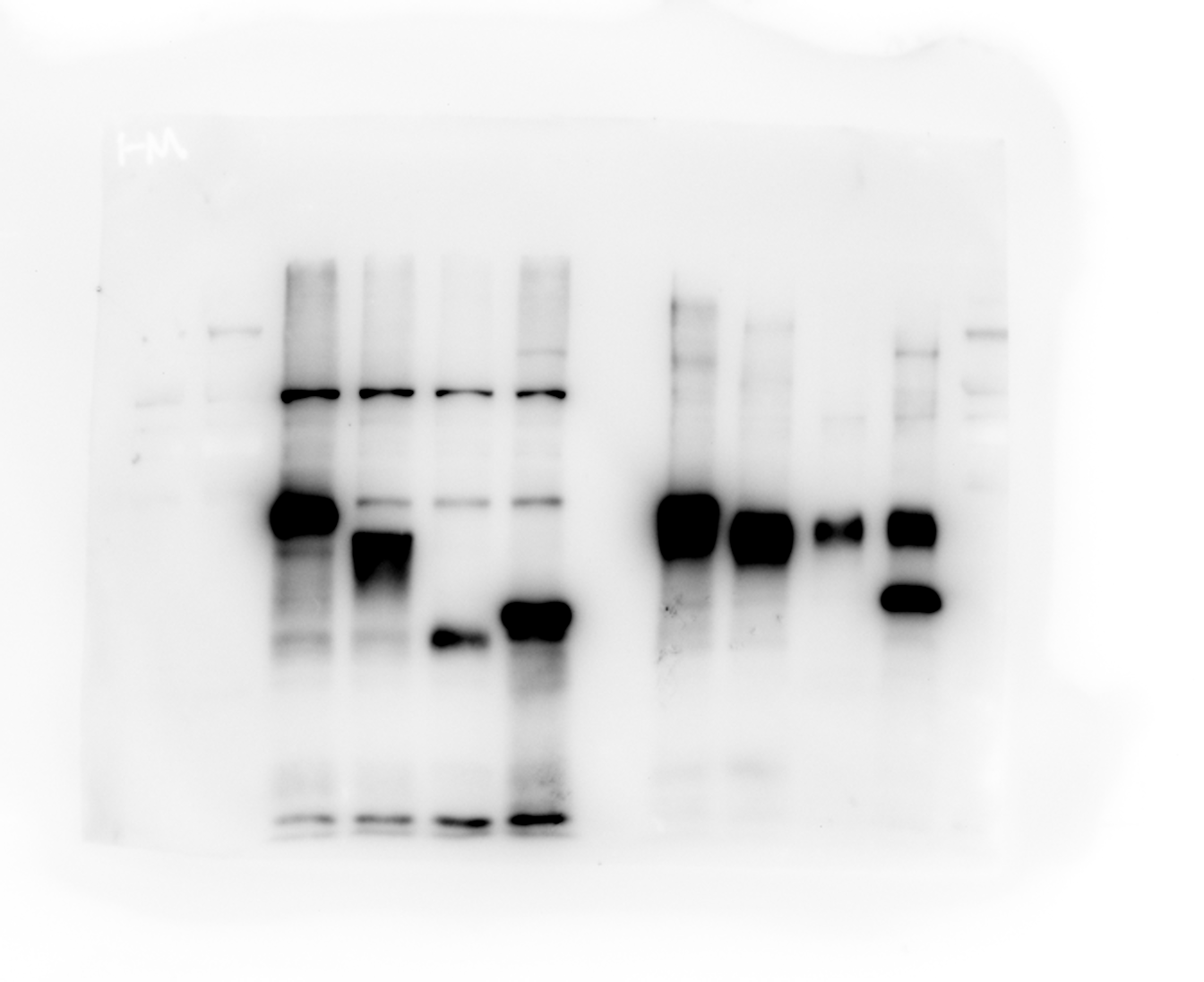

Supplement: Figure 4—source data 8. [file elife-98181-fig4-data8.zip › Figure 4-source data 8 (Myc).tif]

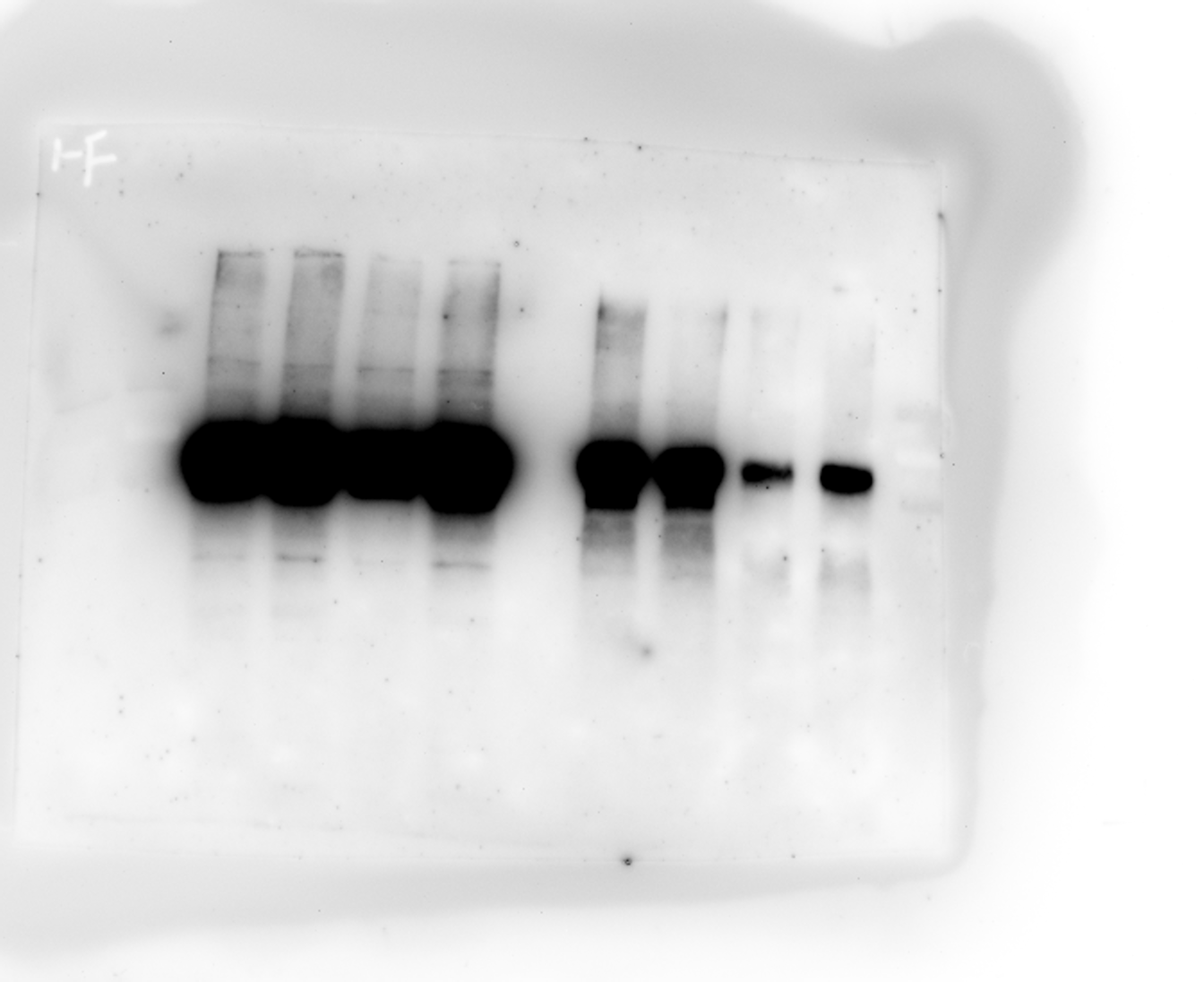

Supplement: Figure 4—source data 8. [file elife-98181-fig4-data8.zip › Figure 4-source data 8 (Flag IP).tif]

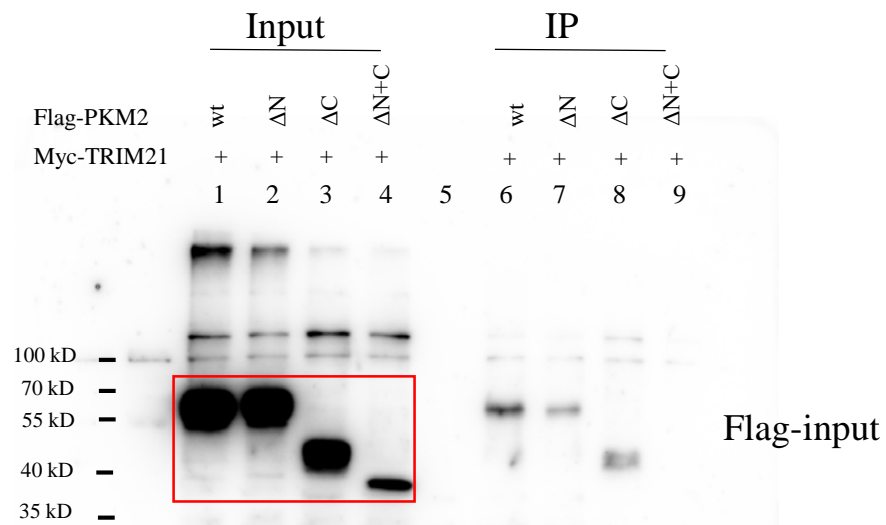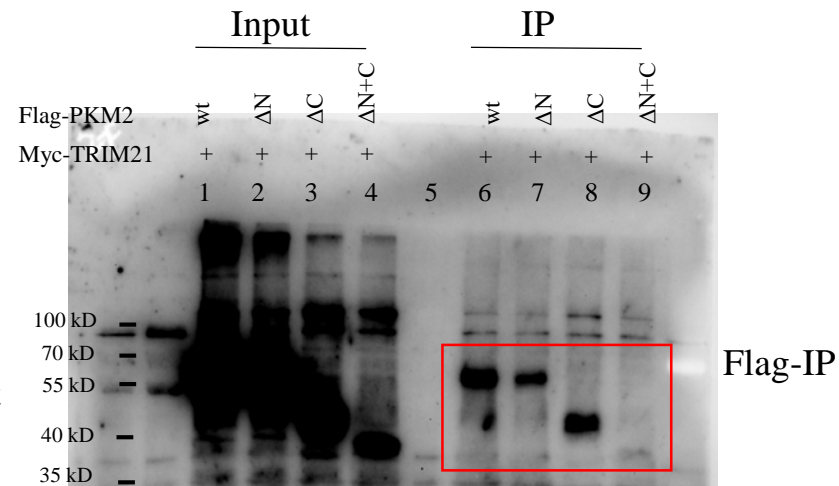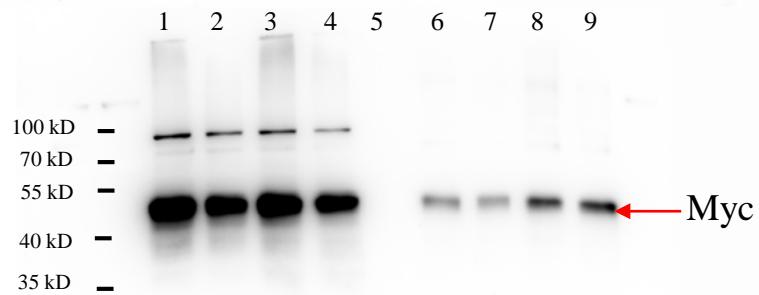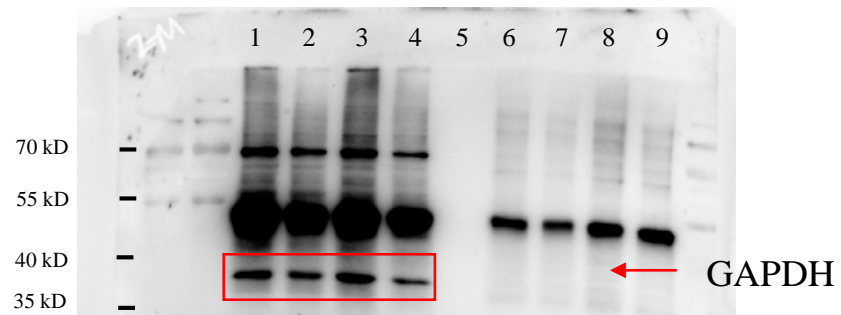

Supplement: Figure 4—source data 9. [file elife-98181-fig4-data9.pdf]

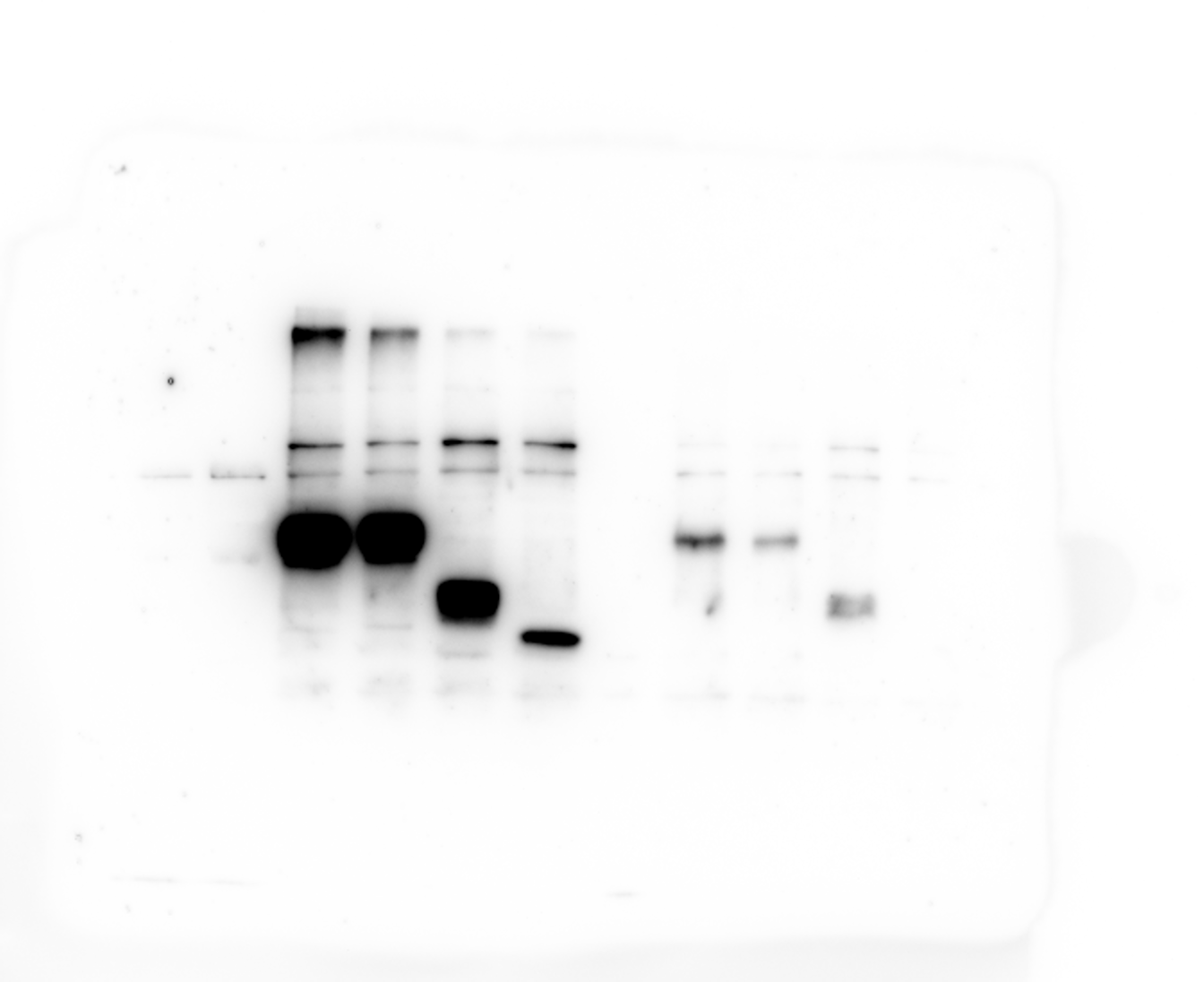

Supplement: Figure 4—source data 10. [file elife-98181-fig4-data10.zip › Figure 4-source data 10 (Flag input).tif]

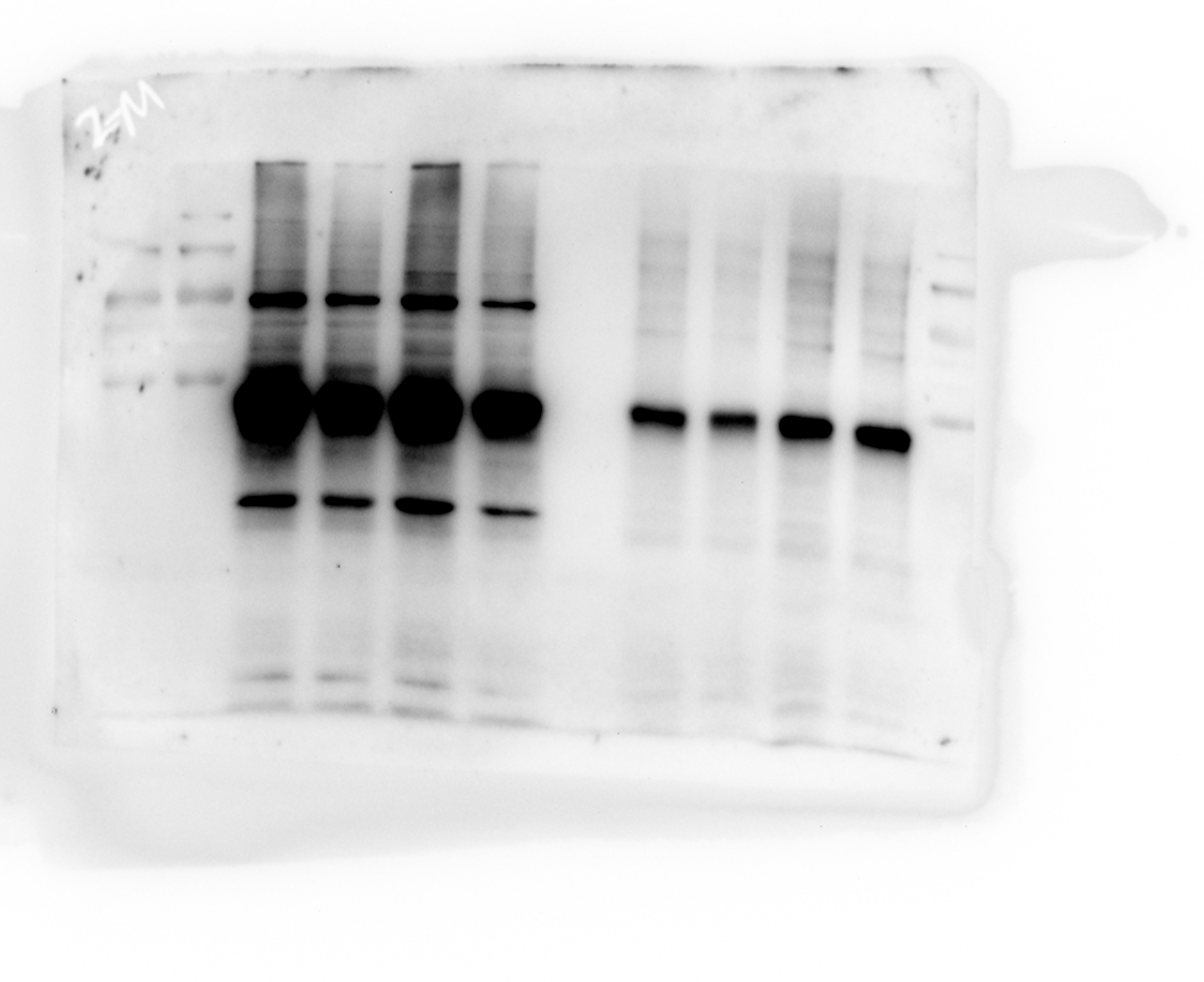

Supplement: Figure 4—source data 10. [file elife-98181-fig4-data10.zip › Figure 4-source data 10 (GAPDH).tif]

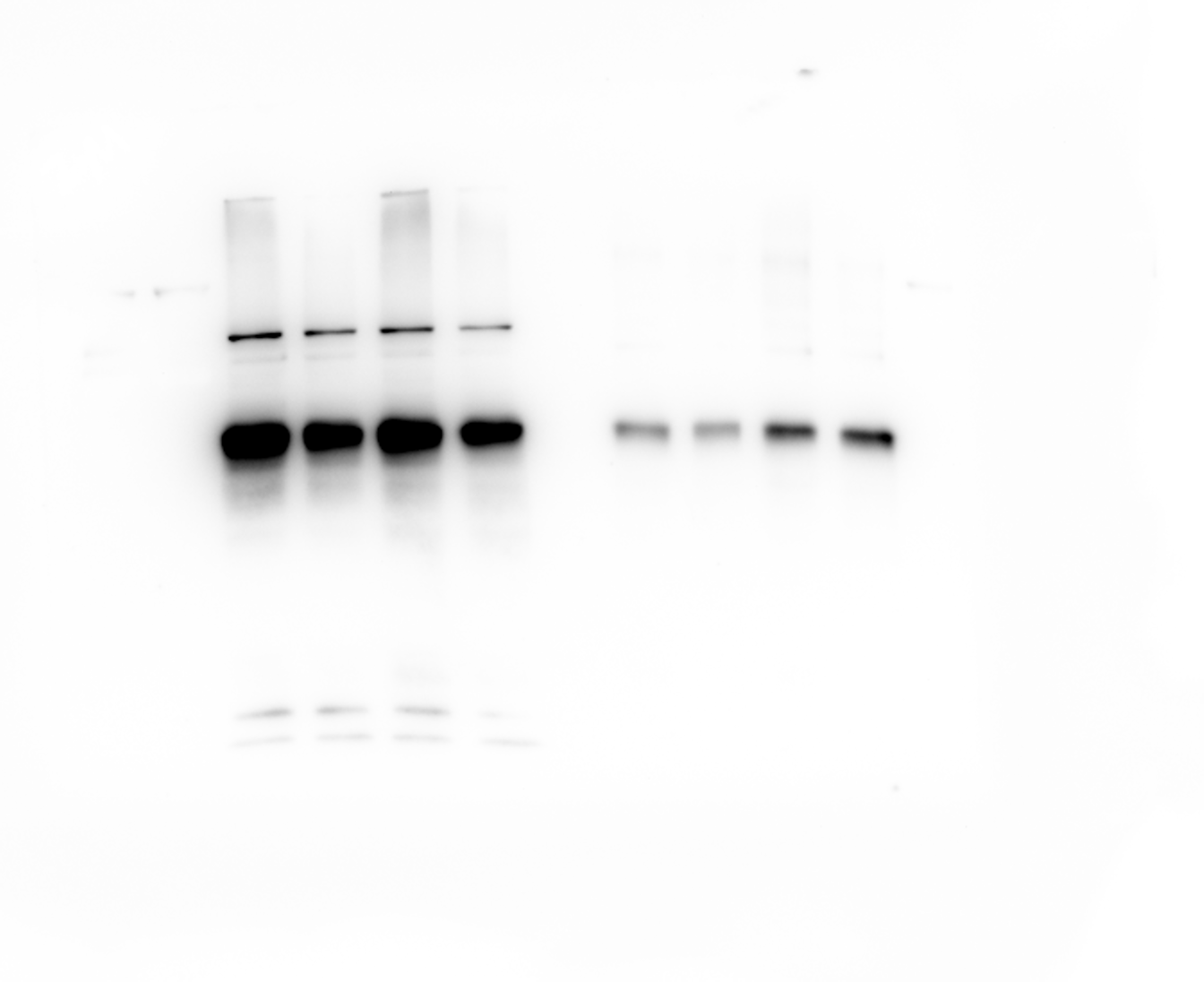

Supplement: Figure 4—source data 10. [file elife-98181-fig4-data10.zip › Figure 4-source data 10 (Myc).tif]

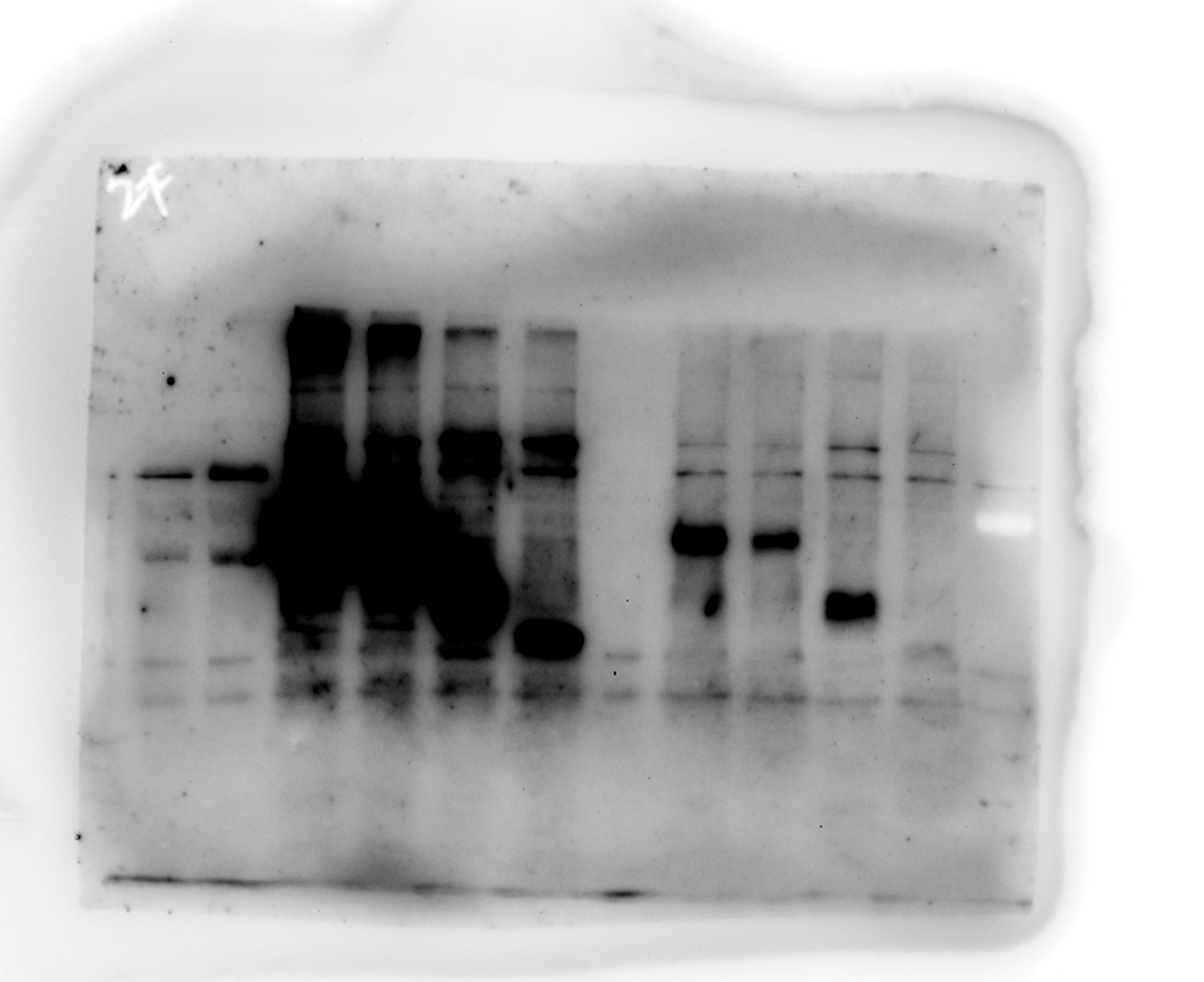

Supplement: Figure 4—source data 10. [file elife-98181-fig4-data10.zip › Figure 4-source data 10 (Flag IP).tif]

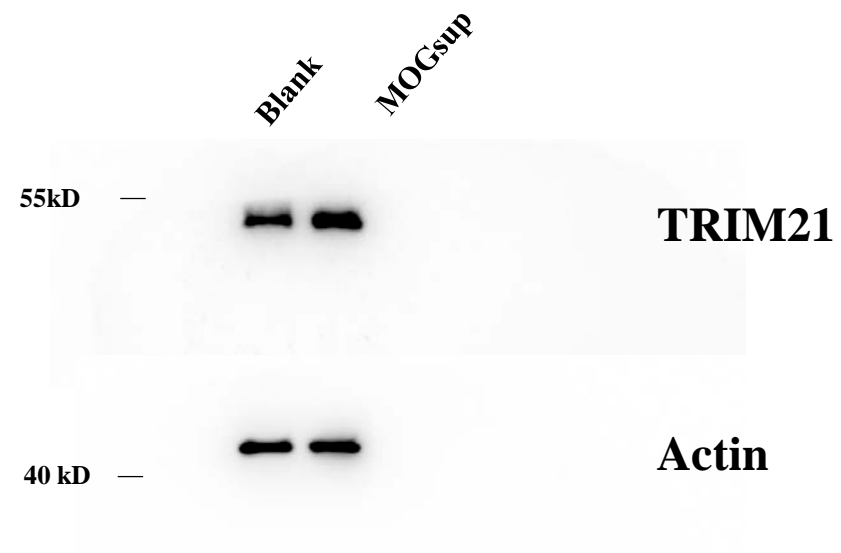

Supplement: Figure 5—source data 2. [file elife-98181-fig5-data2.pdf]

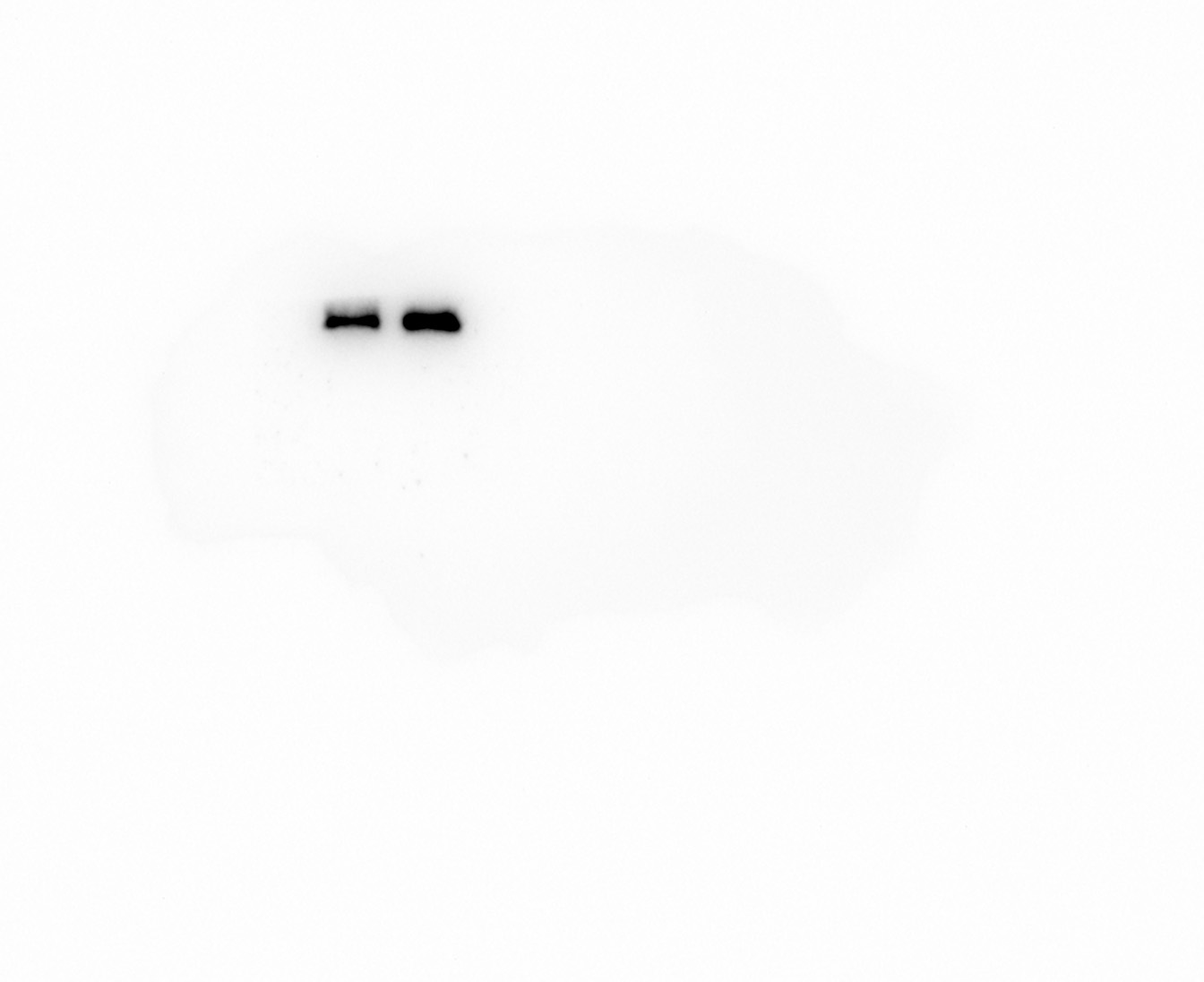

Supplement: Figure 5—source data 3. [file elife-98181-fig5-data3.zip › Figure 5-source data 3 (TRIM21).jpg]

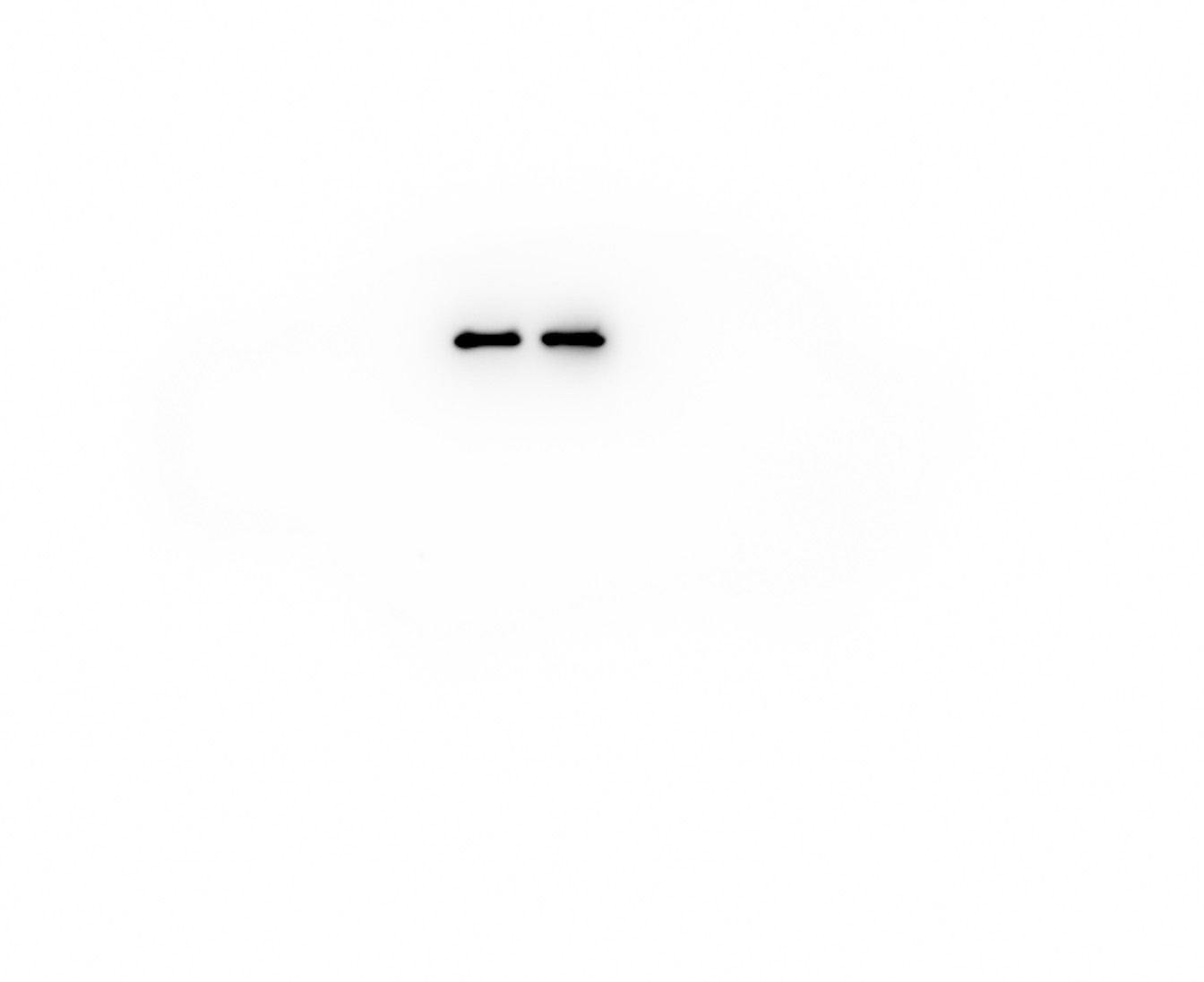

Supplement: Figure 5—source data 3. [file elife-98181-fig5-data3.zip › Figure 5-source data 3 (Actin).jpg]

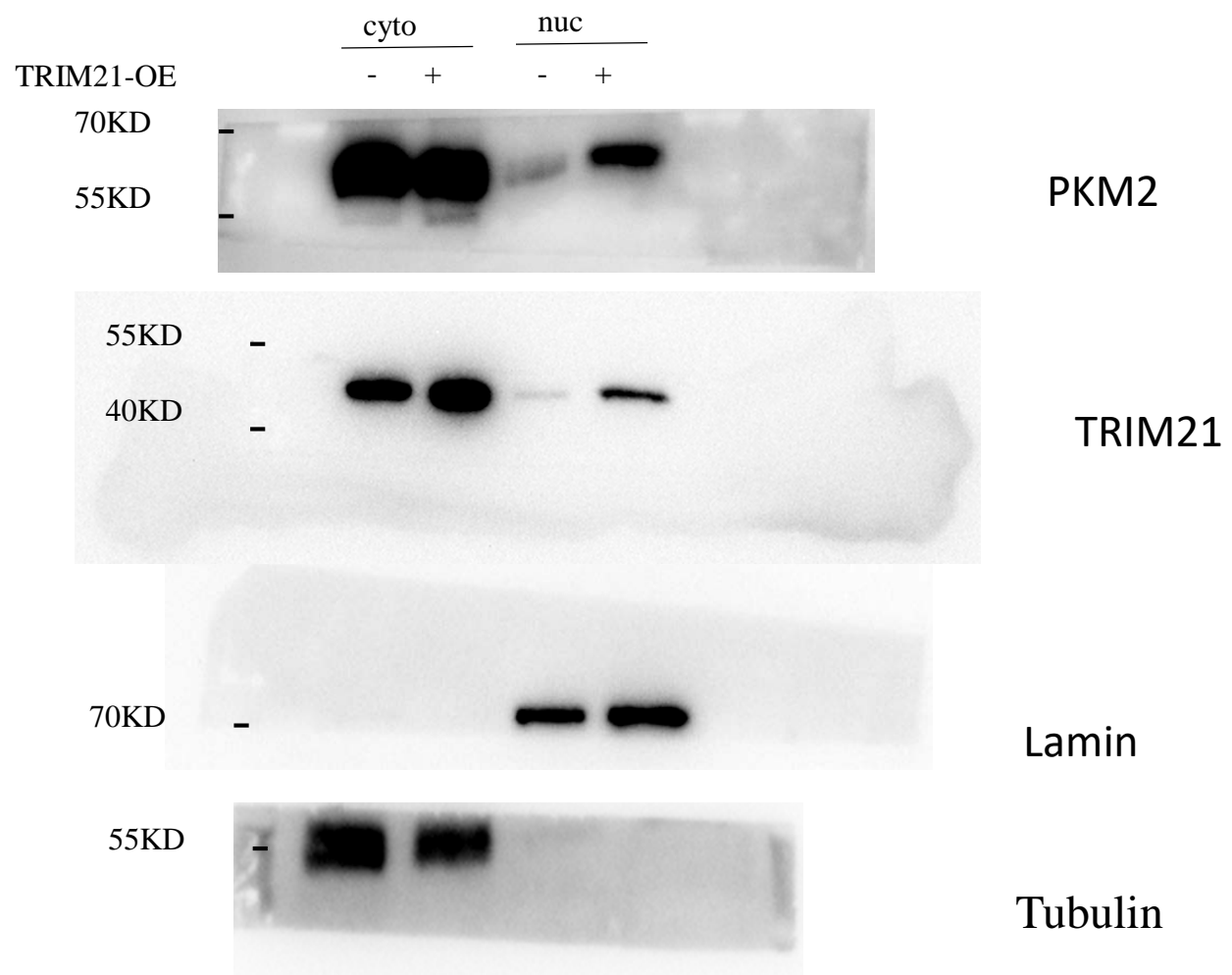

Supplement: Figure 6—source data 2. [file elife-98181-fig6-data2.pdf]

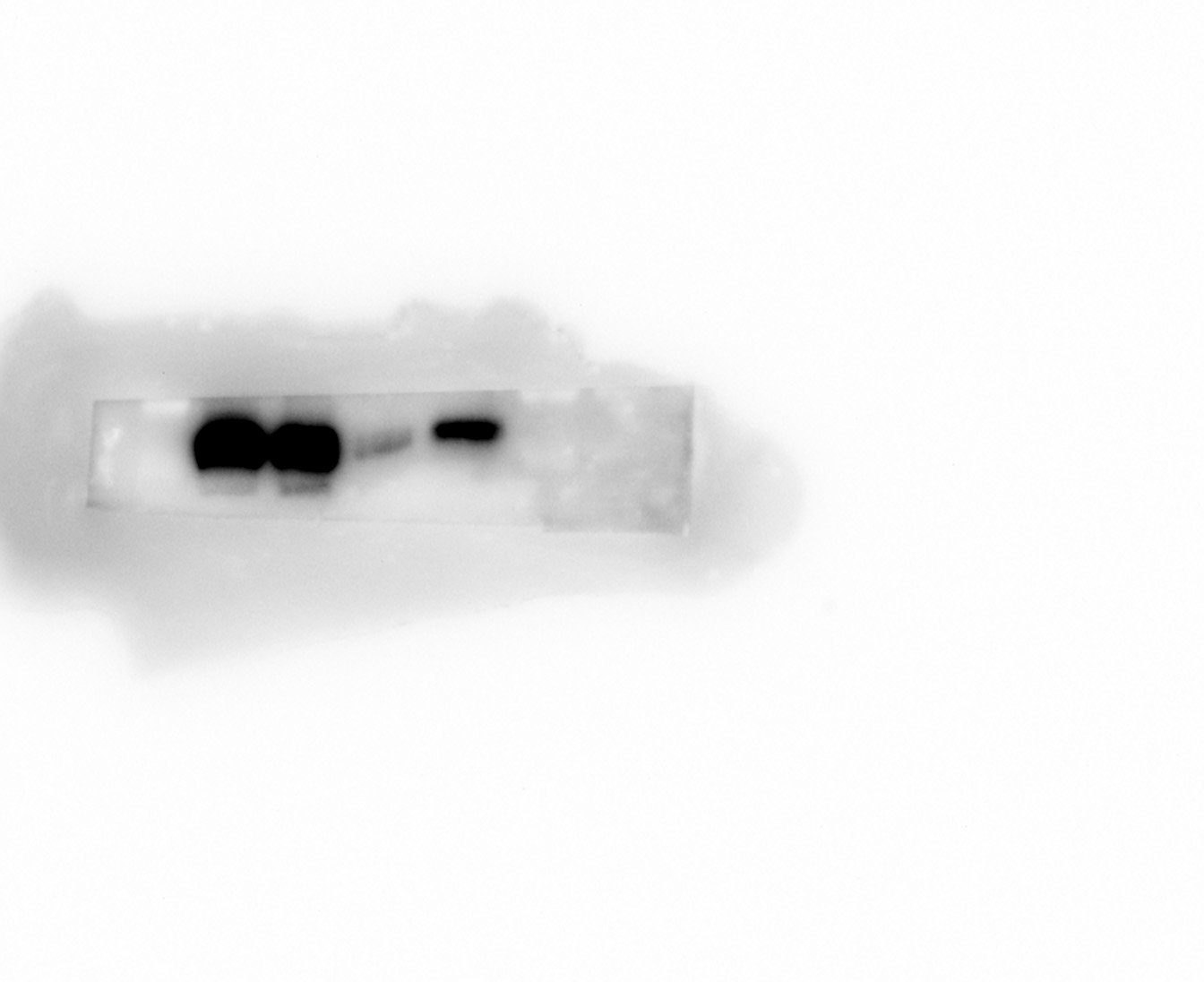

Supplement: Figure 6—source data 3. [file elife-98181-fig6-data3.zip › Figure 6-source data 3 (PKM2).jpg]

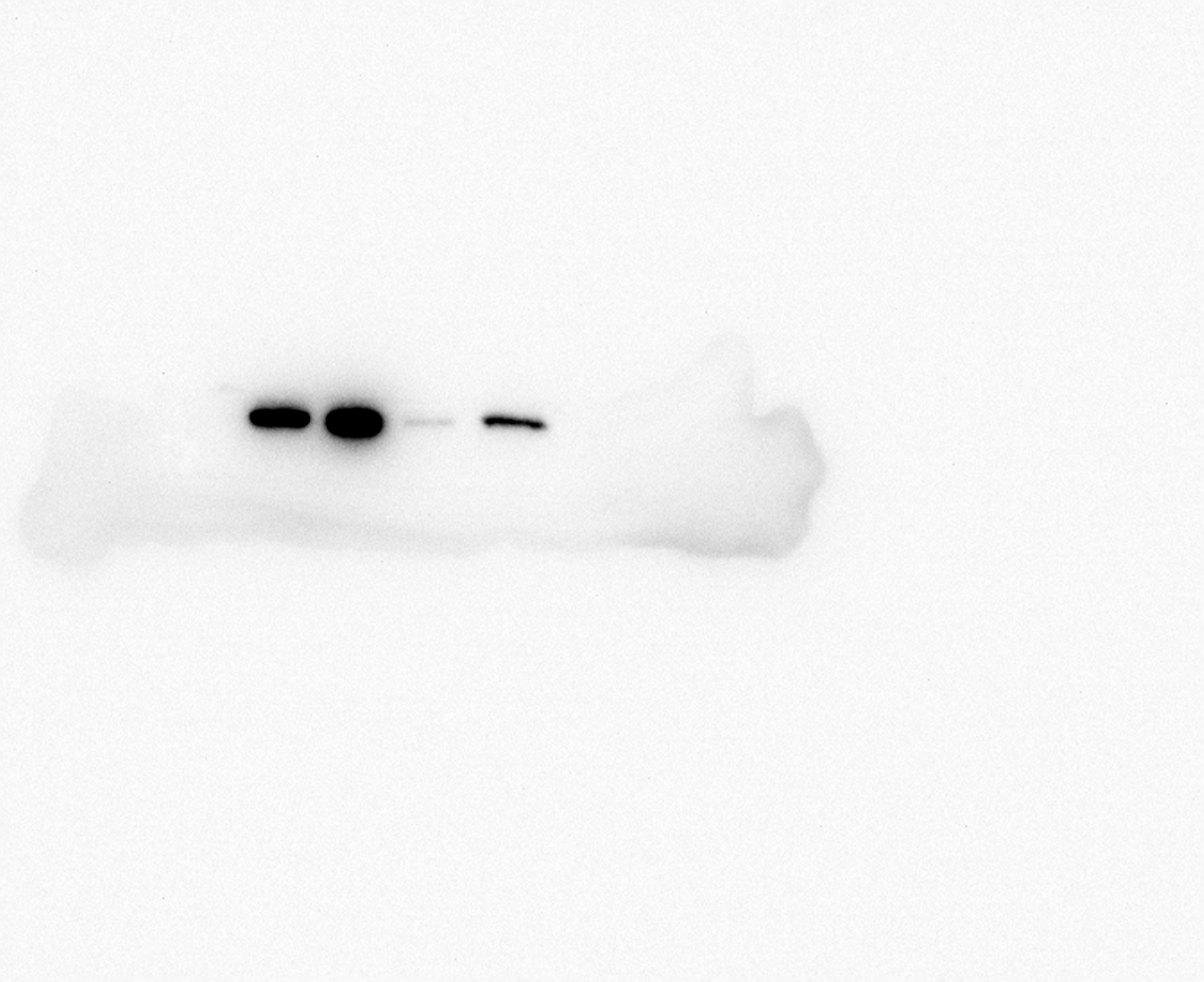

Supplement: Figure 6—source data 3. [file elife-98181-fig6-data3.zip › Figure 6-source data 3 (TRIM21).tif]

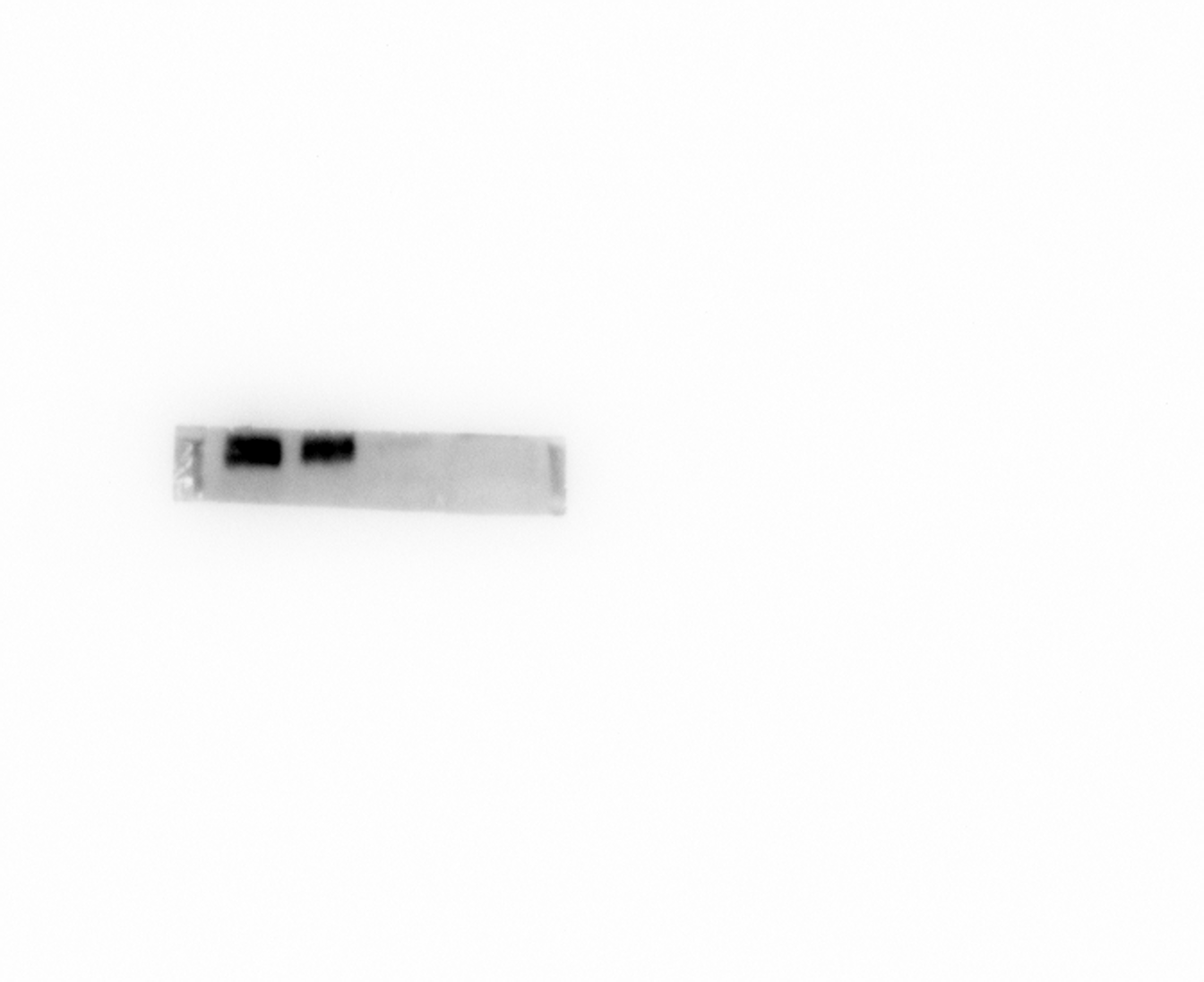

Supplement: Figure 6—source data 3. [file elife-98181-fig6-data3.zip › Figure 6-source data 3 (Tubulin).tif]

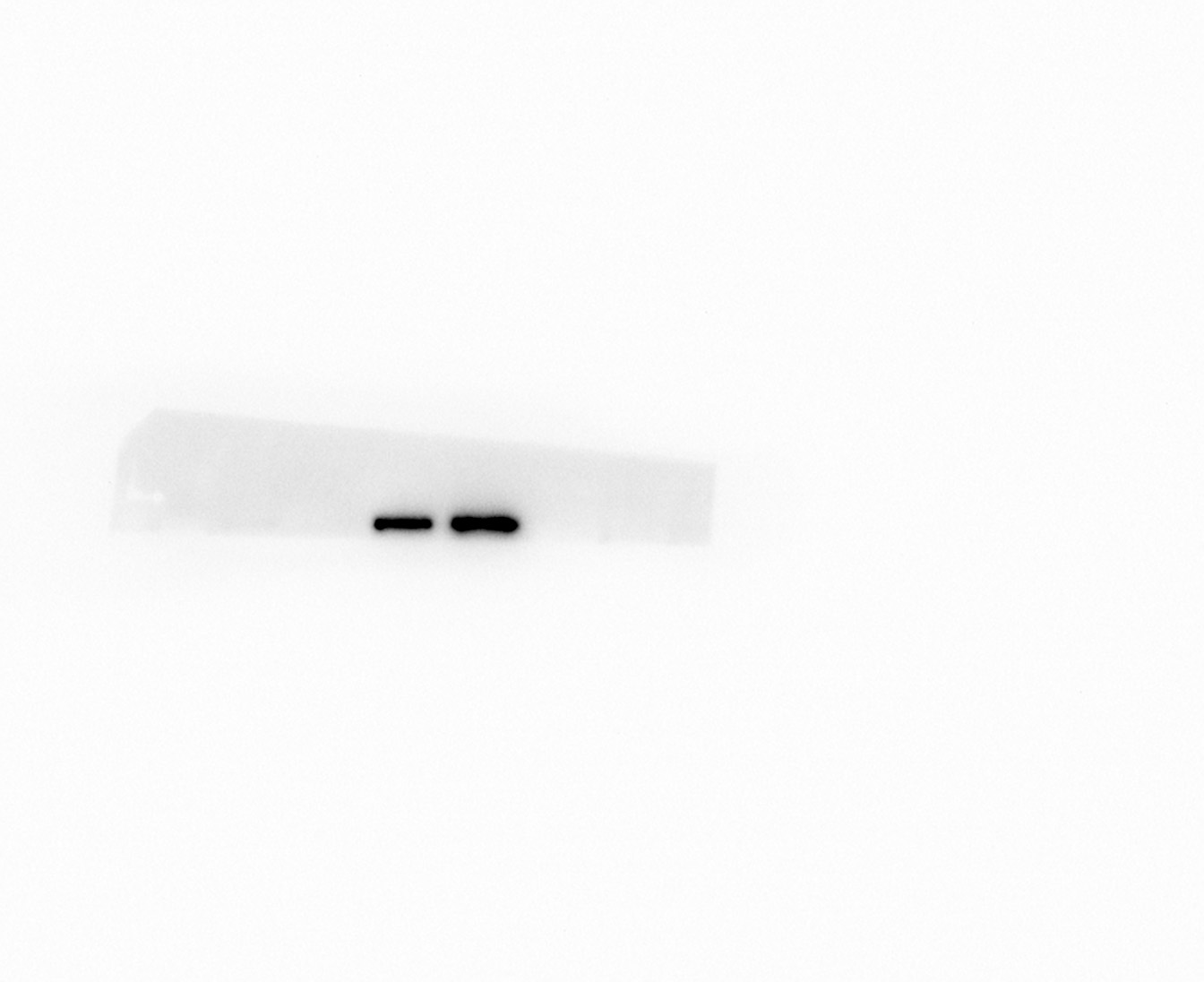

Supplement: Figure 6—source data 3. [file elife-98181-fig6-data3.zip › Figure 6-source data 3 (Lamin).jpg]

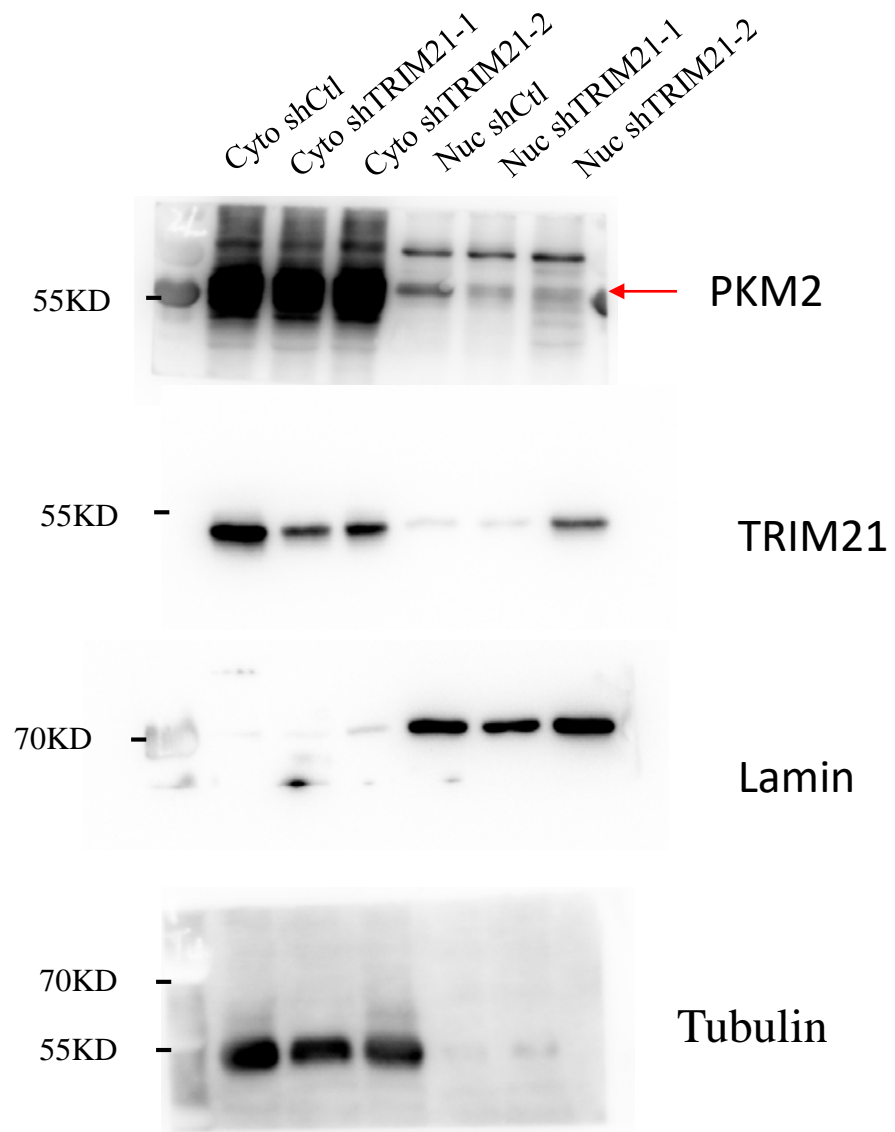

Supplement: Figure 6—source data 4. [file elife-98181-fig6-data4.pdf]

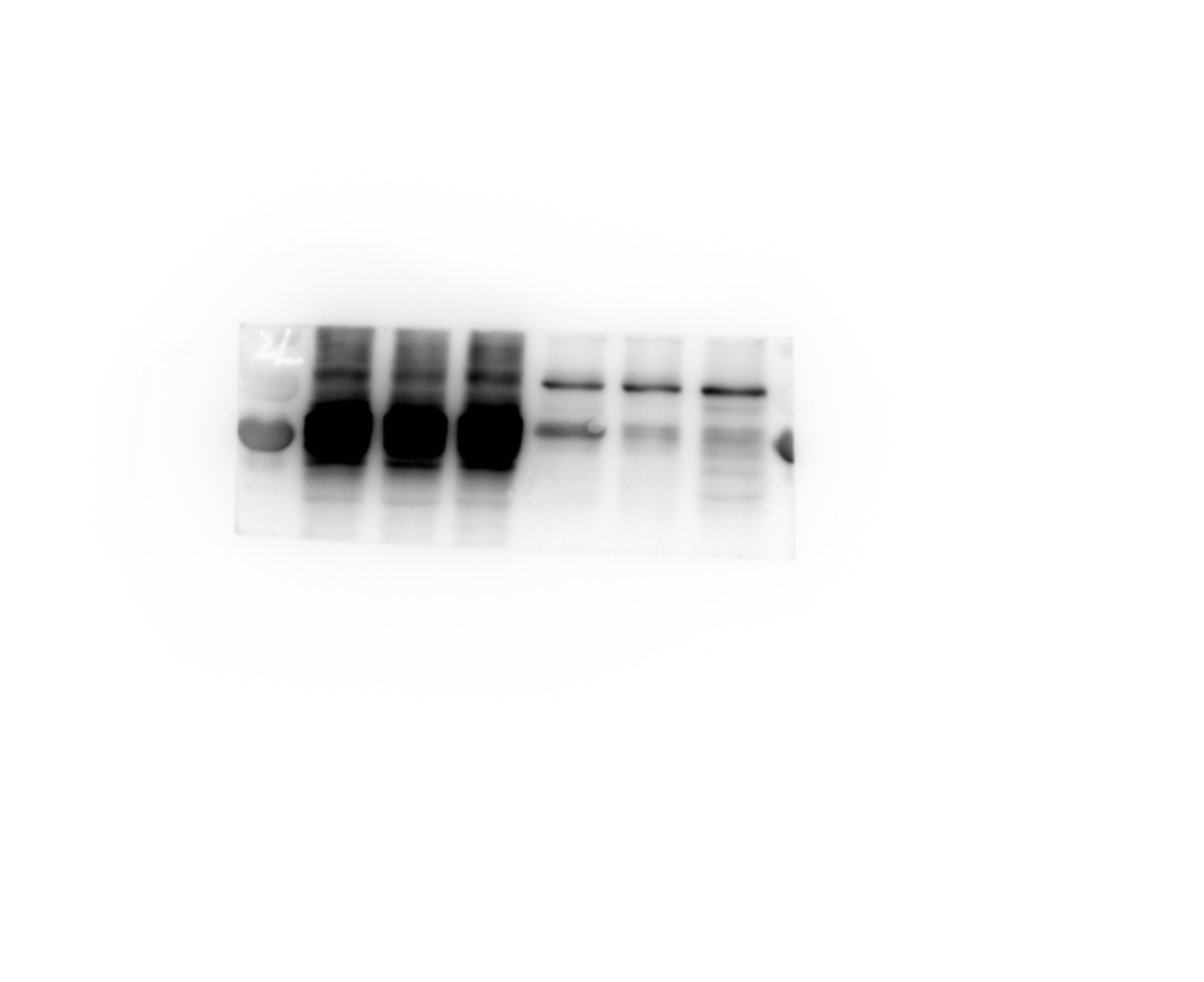

Supplement: Figure 6—source data 5. [file elife-98181-fig6-data5.zip › Figure 6-source data 5 (PKM2).tif]

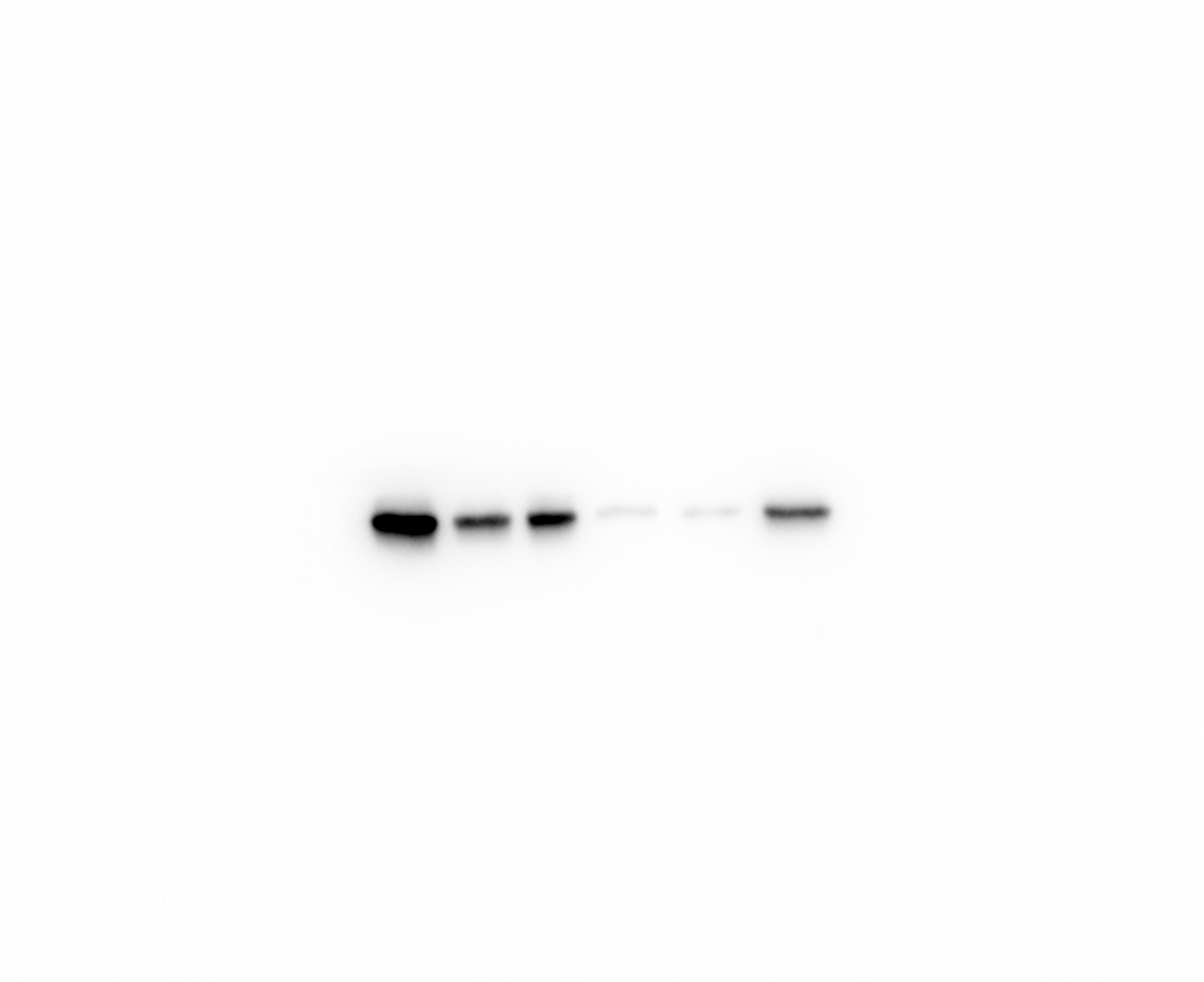

Supplement: Figure 6—source data 5. [file elife-98181-fig6-data5.zip › Figure 6-source data 5 (TRIM21).tif]

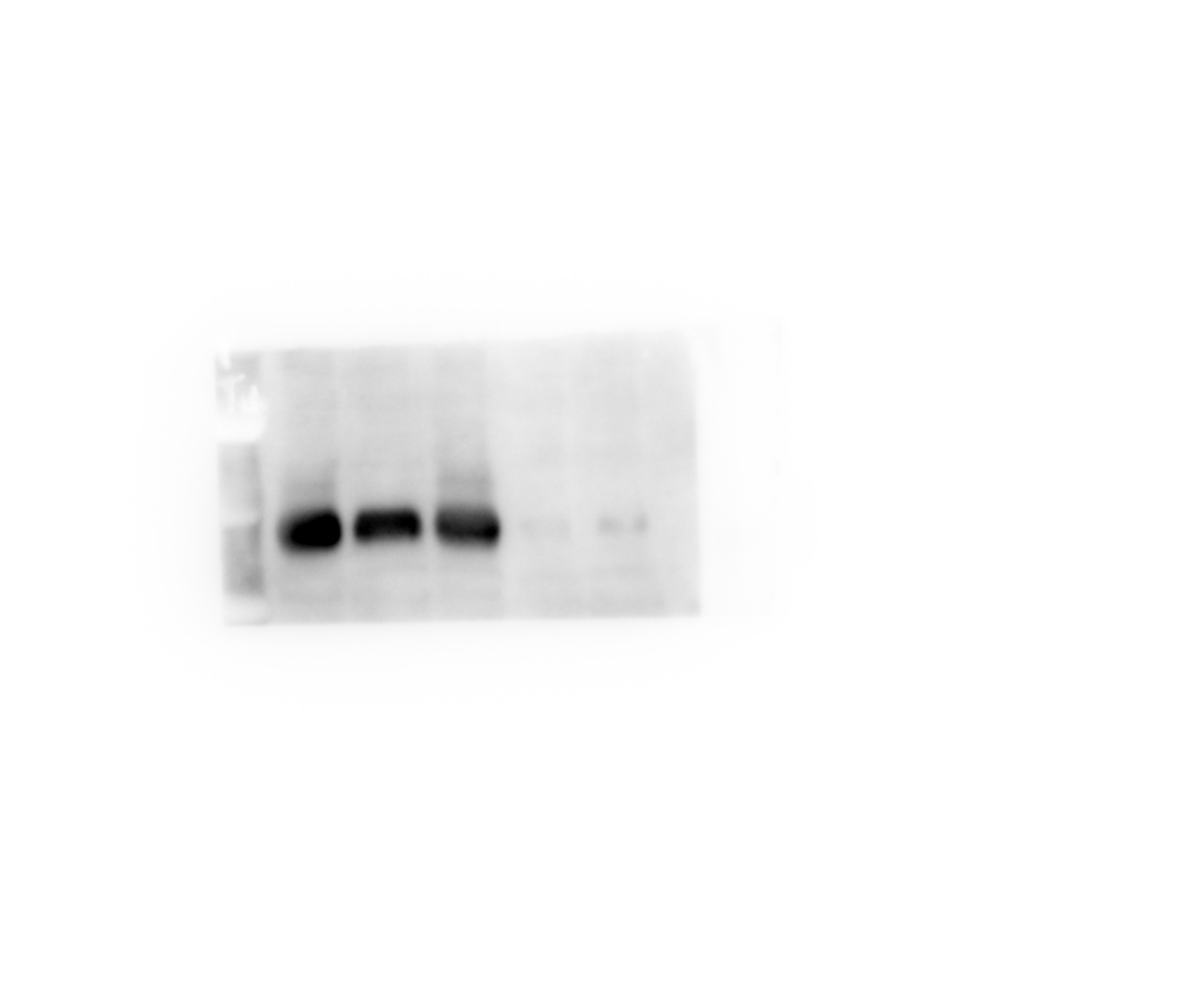

Supplement: Figure 6—source data 5. [file elife-98181-fig6-data5.zip › Figure 6-source data 5 (Tubulin).tif]

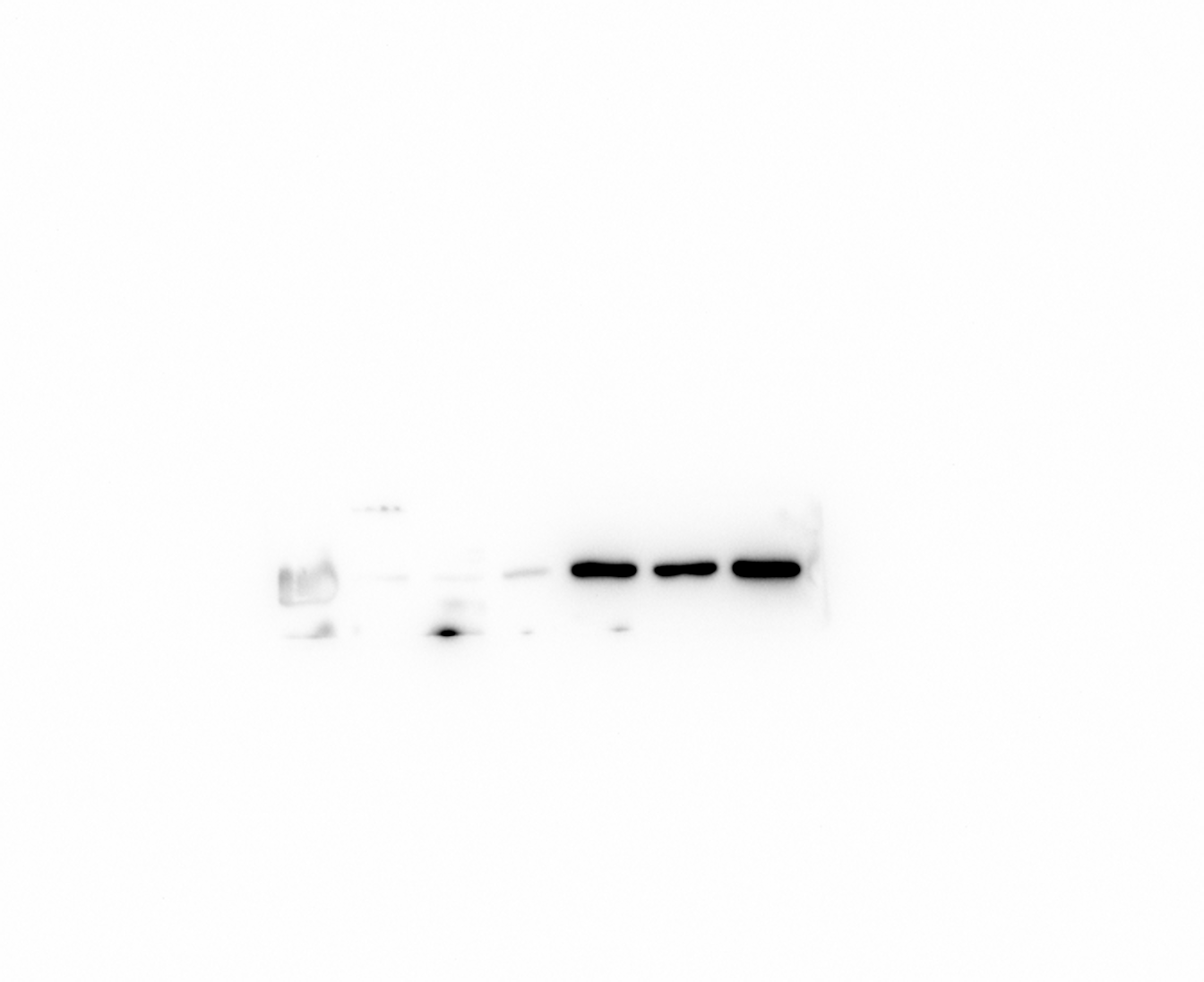

Supplement: Figure 6—source data 5. [file elife-98181-fig6-data5.zip › Figure 6-source data 5 (Lamin).tif]

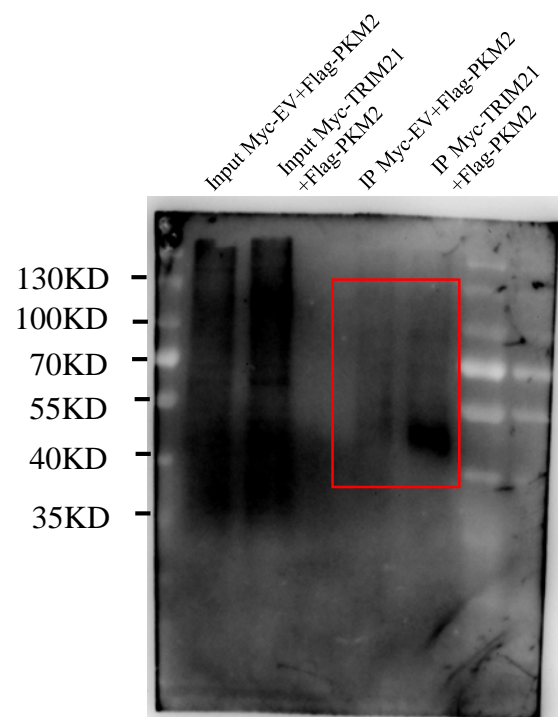

K63-Ub IP-Flag

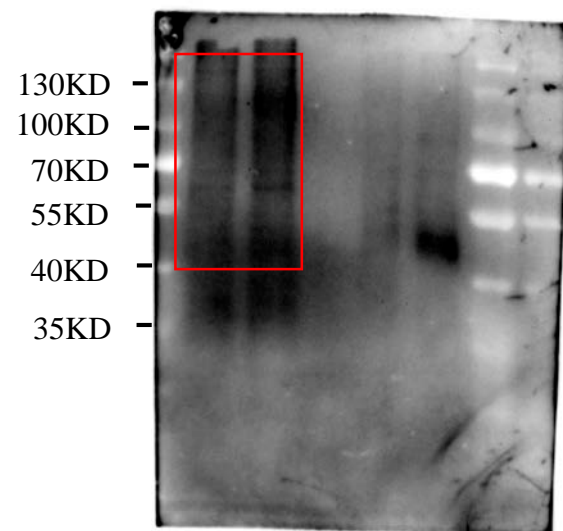

K63-Ub Input

Supplement: Figure 6—source data 6. [file elife-98181-fig6-data6.pdf]

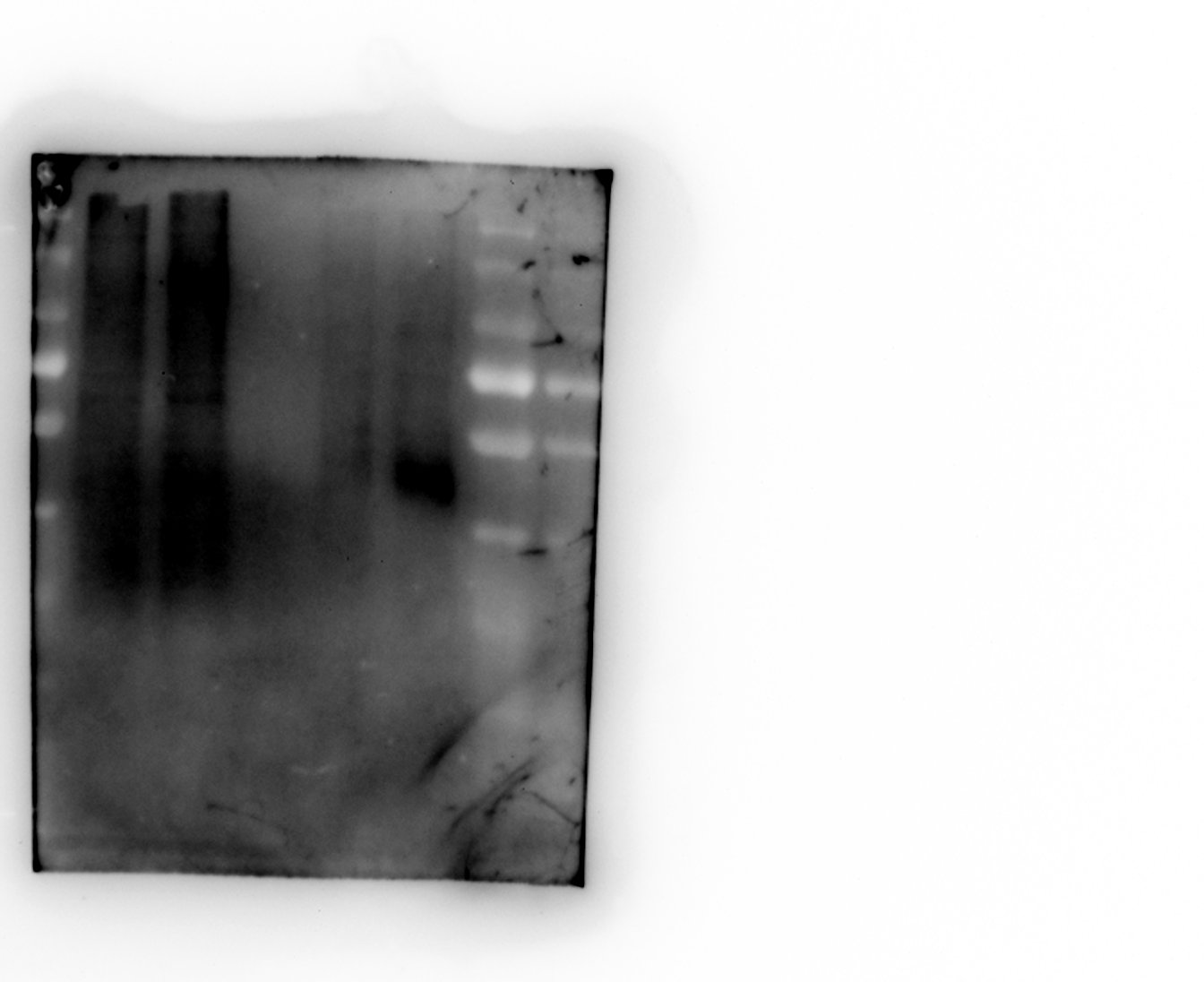

Supplement: Figure 6—source data 7. [file elife-98181-fig6-data7.zip › Figure 6-source data 7ú¿K63 IPú⌐.jpg]

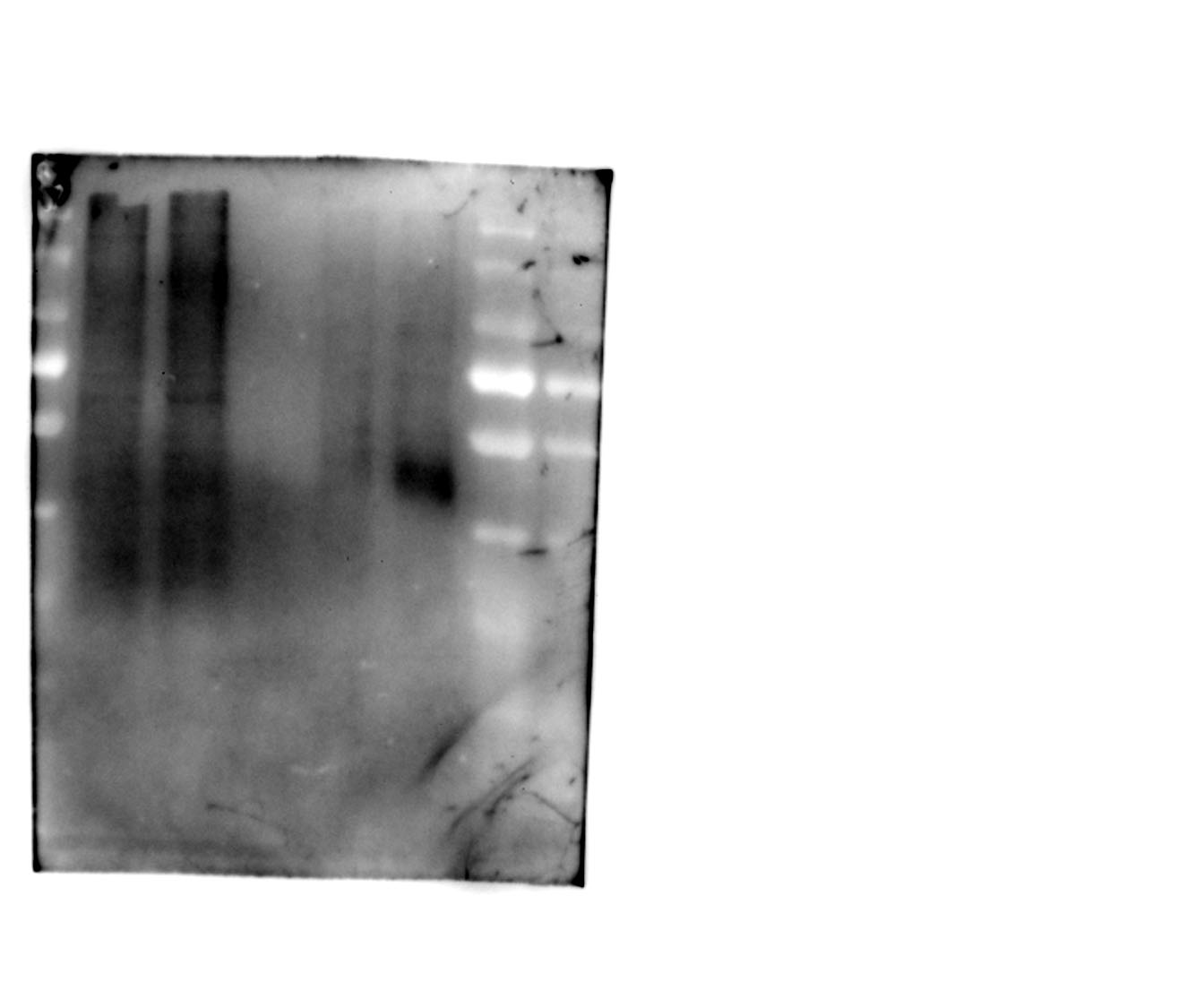

Supplement: Figure 6—source data 7. [file elife-98181-fig6-data7.zip › Figure 6-source data 7(K63 input).jpg]

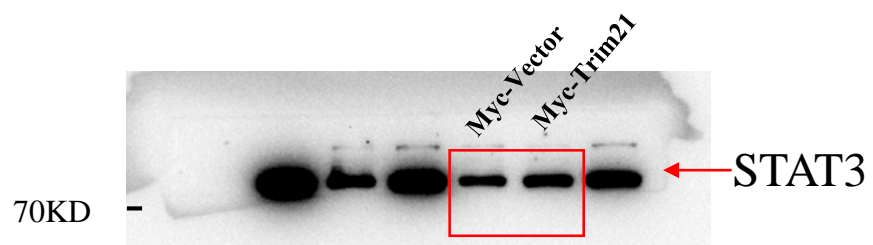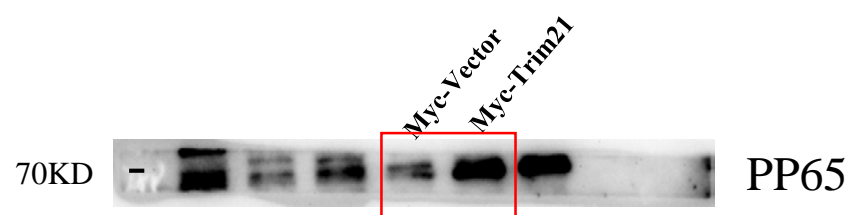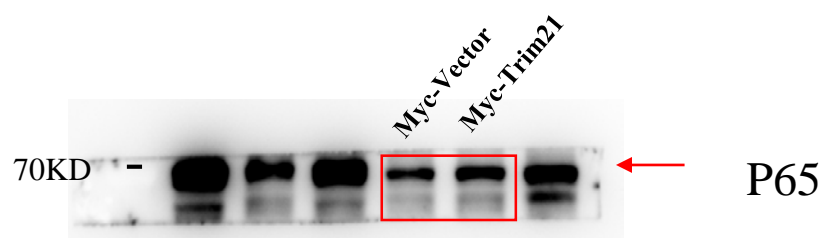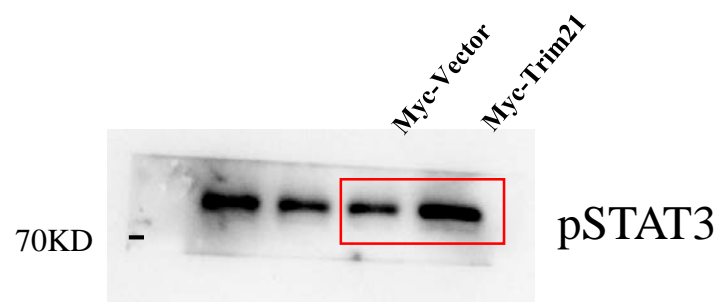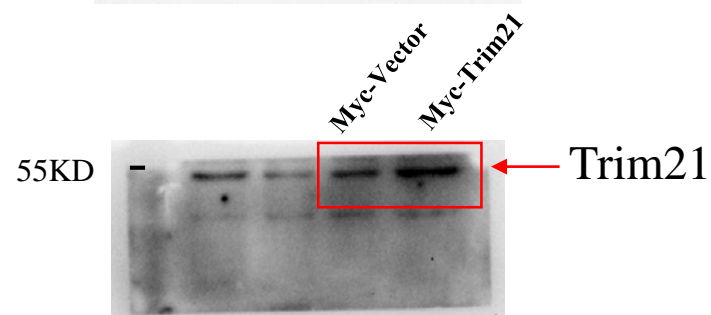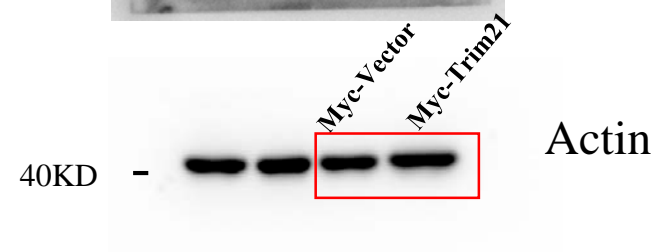

Supplement: Figure 6—source data 8. [file elife-98181-fig6-data8.pdf]

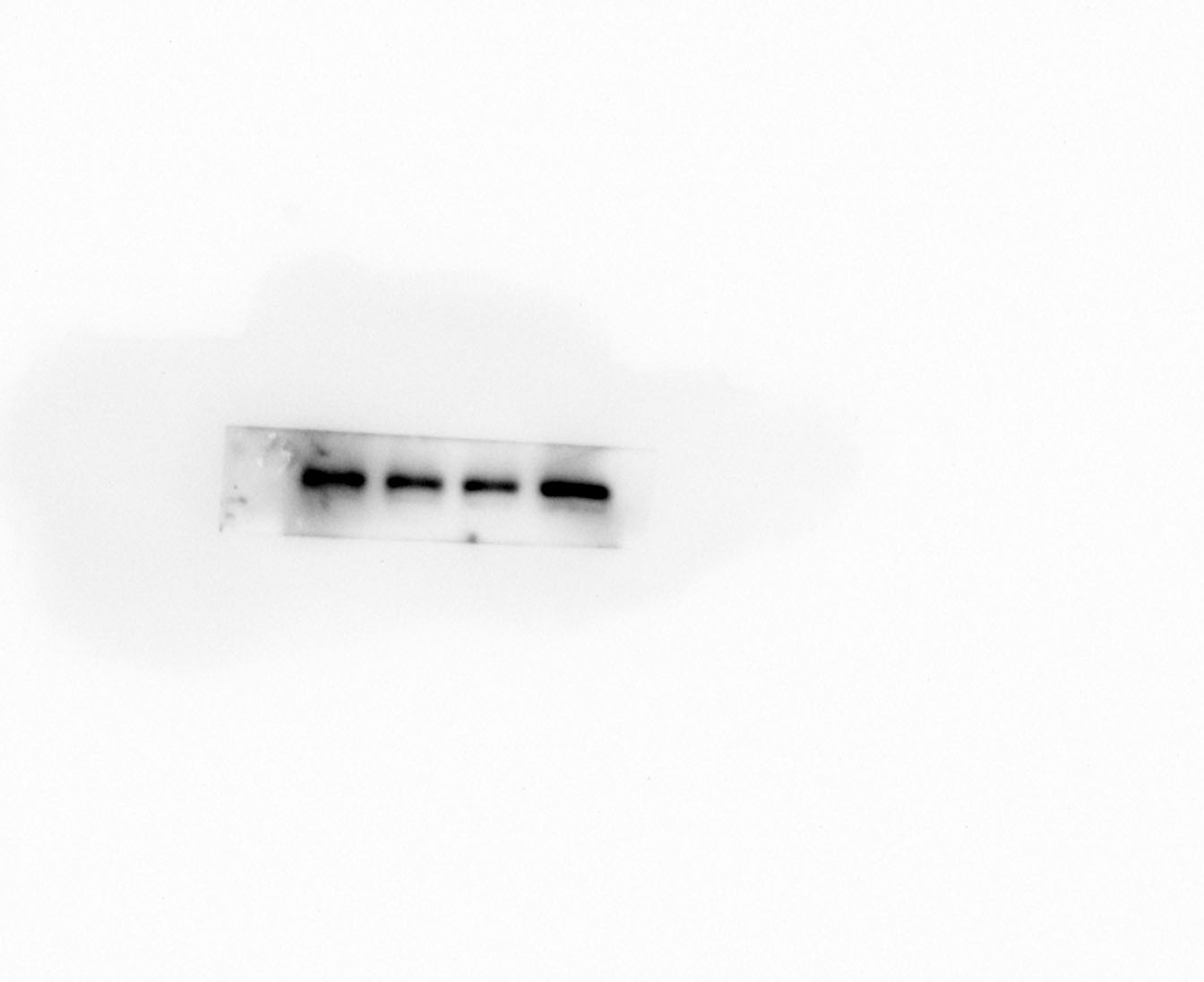

Supplement: Figure 6—source data 9. [file elife-98181-fig6-data9.zip › Figure 6-source data 9ú¿P-STAT3).jpg]

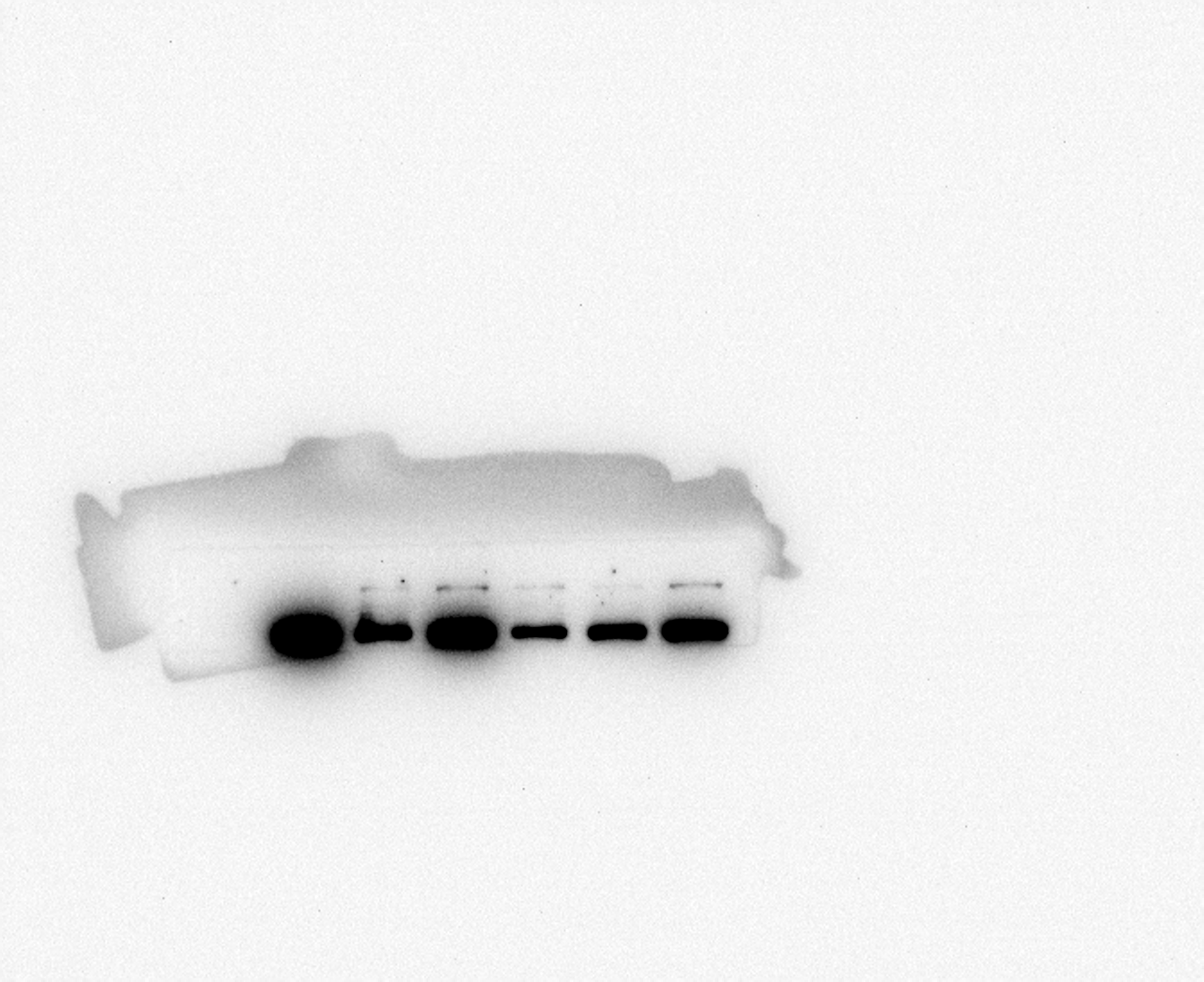

Supplement: Figure 6—source data 9. [file elife-98181-fig6-data9.zip › Figure 6-source data 9ú¿STAT3).tif]

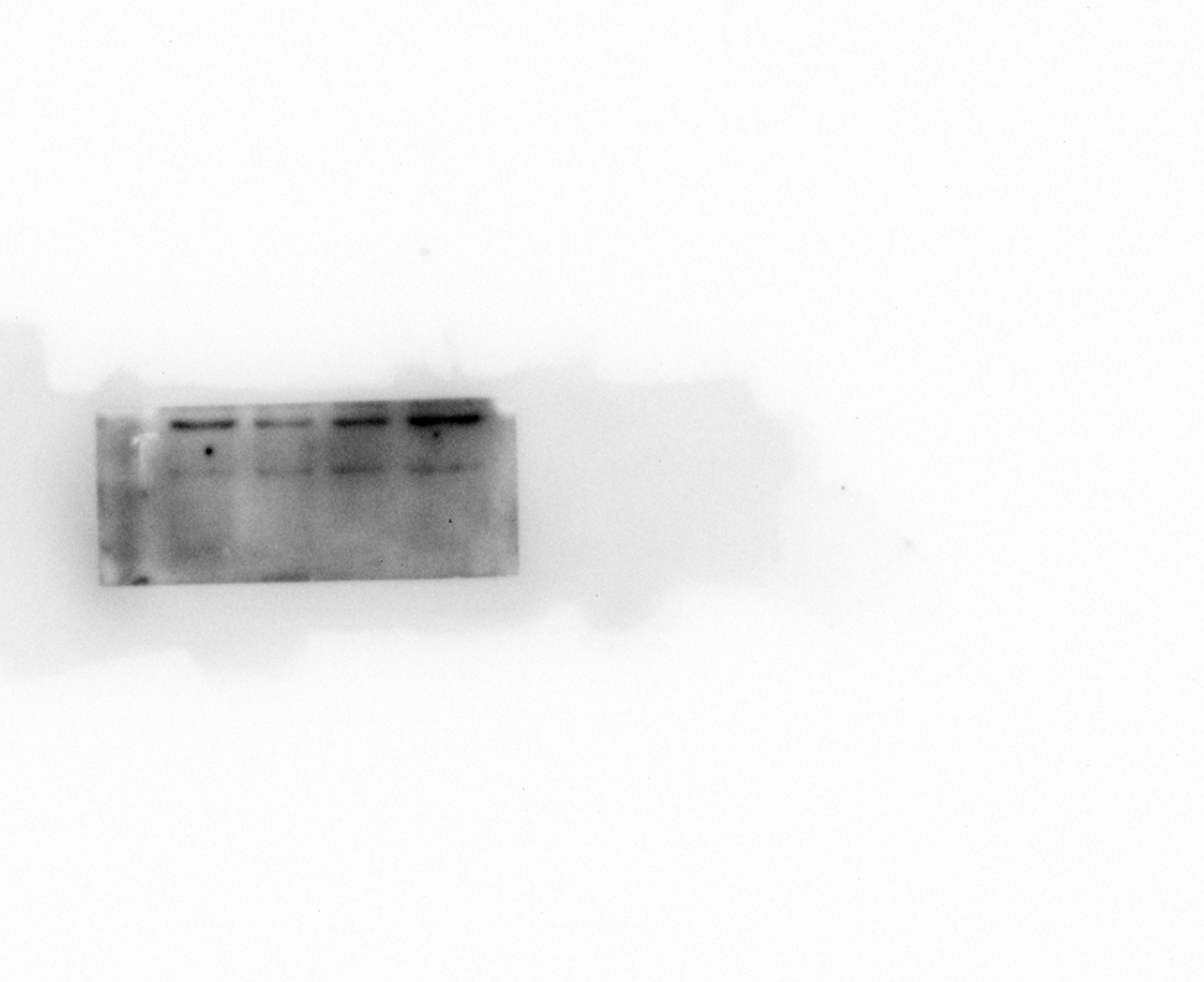

Supplement: Figure 6—source data 9. [file elife-98181-fig6-data9.zip › Figure 6-source data 9ú¿Trim21).tif]

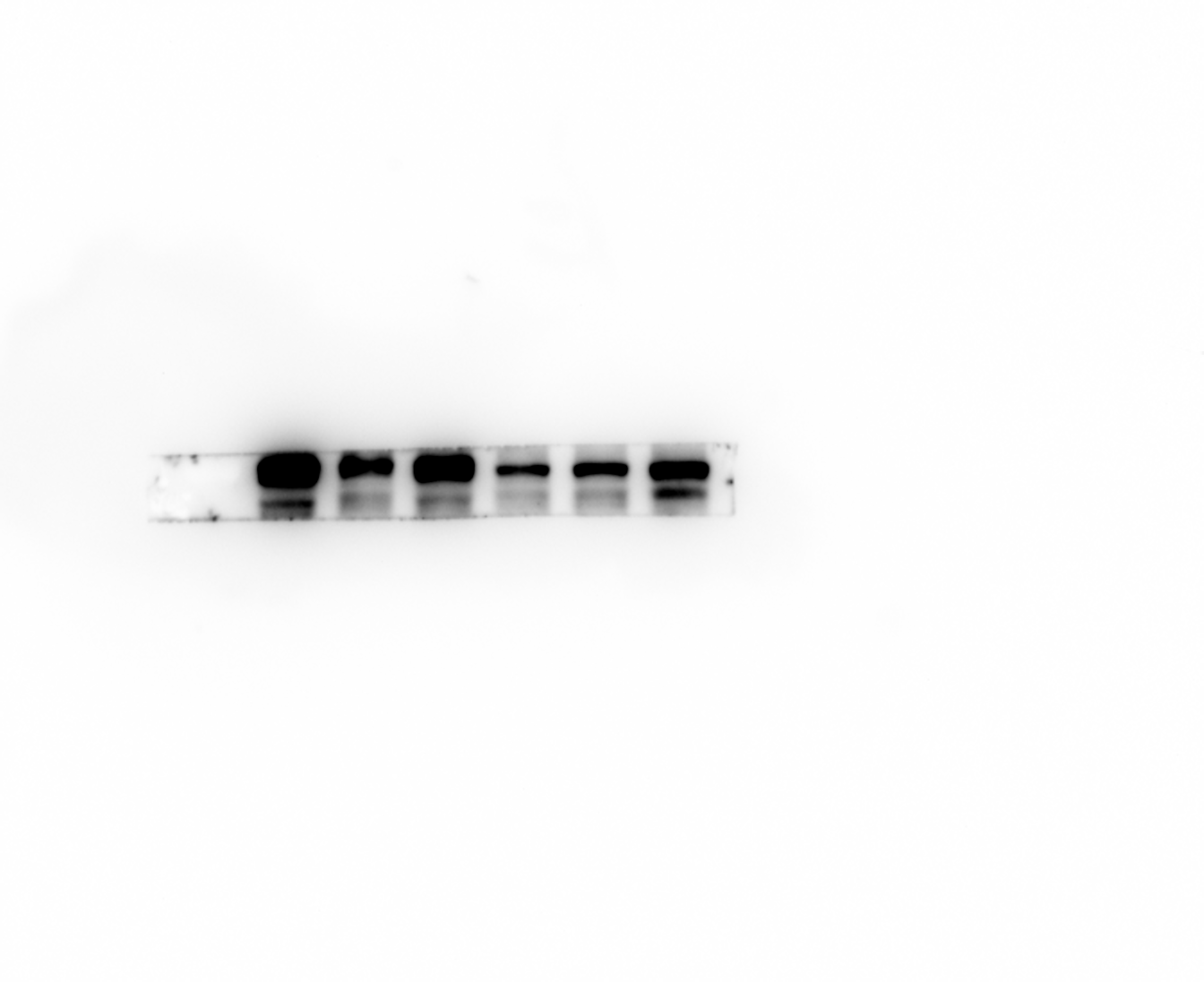

Supplement: Figure 6—source data 9. [file elife-98181-fig6-data9.zip › Figure 6-source data 9ú¿p65ú⌐.tif]

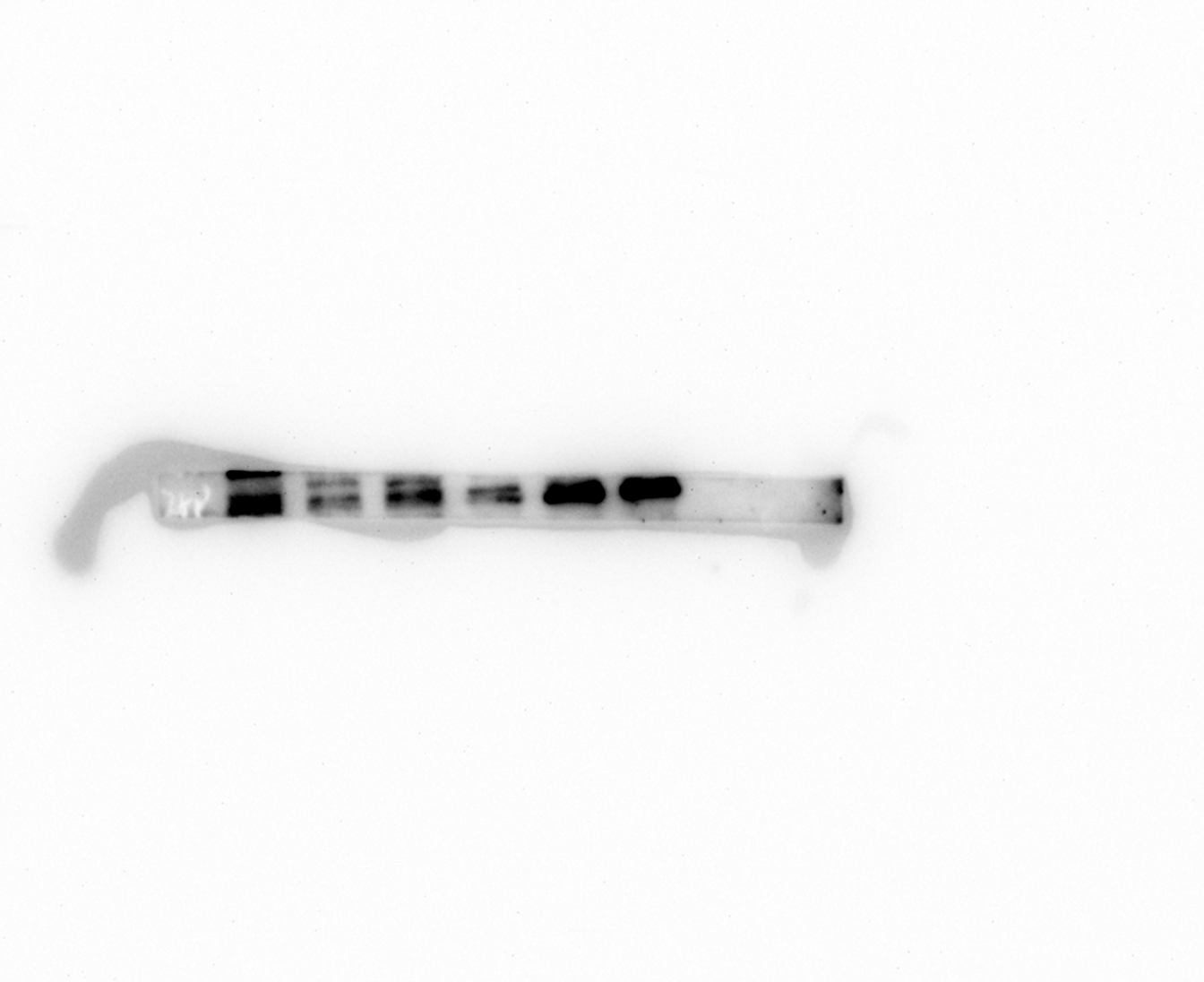

Supplement: Figure 6—source data 9. [file elife-98181-fig6-data9.zip › Figure 6-source data 9ú¿pp65).tif]

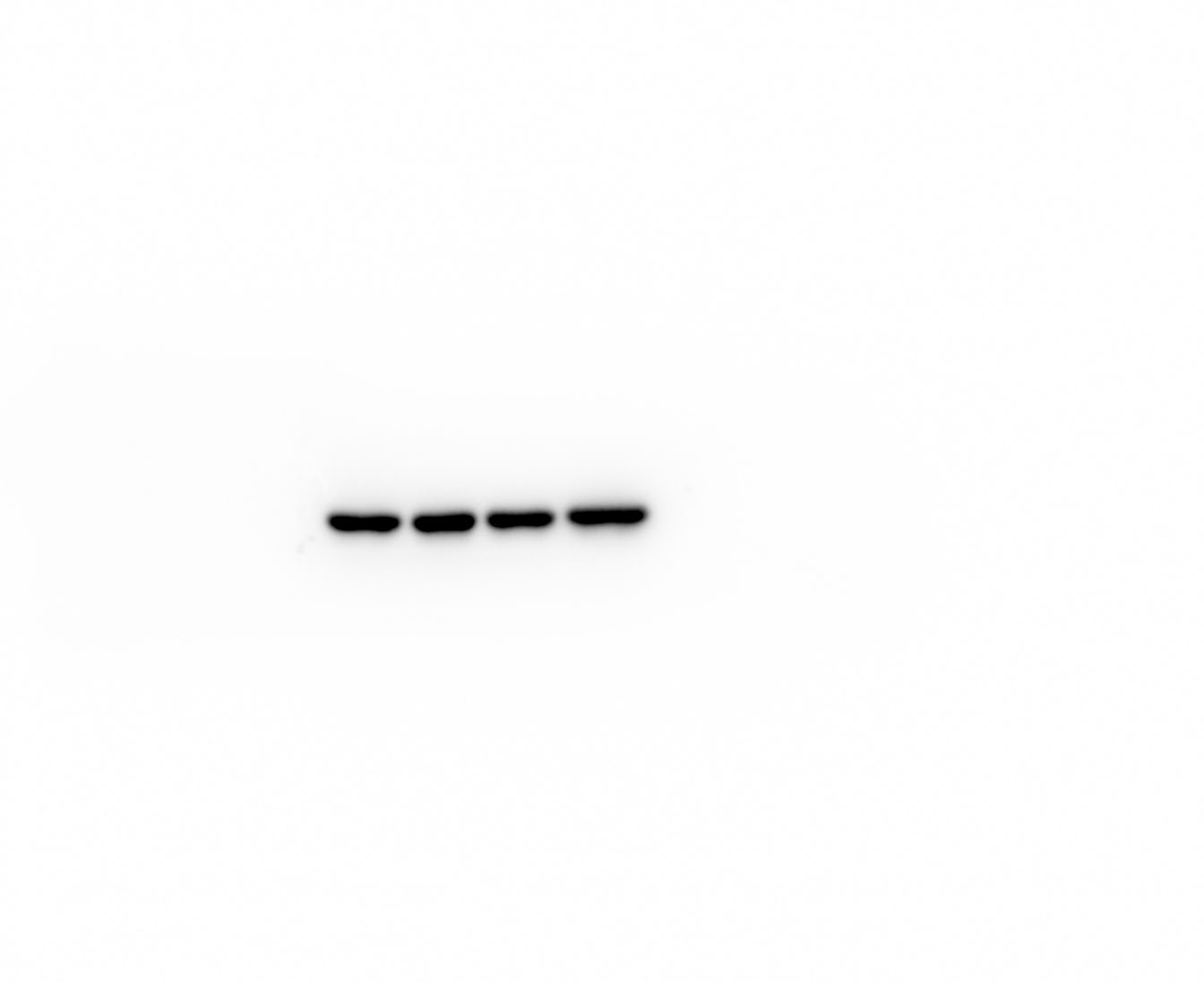

Supplement: Figure 6—source data 9. [file elife-98181-fig6-data9.zip › Figure 6-source data 9ú¿Actinú⌐.jpg]

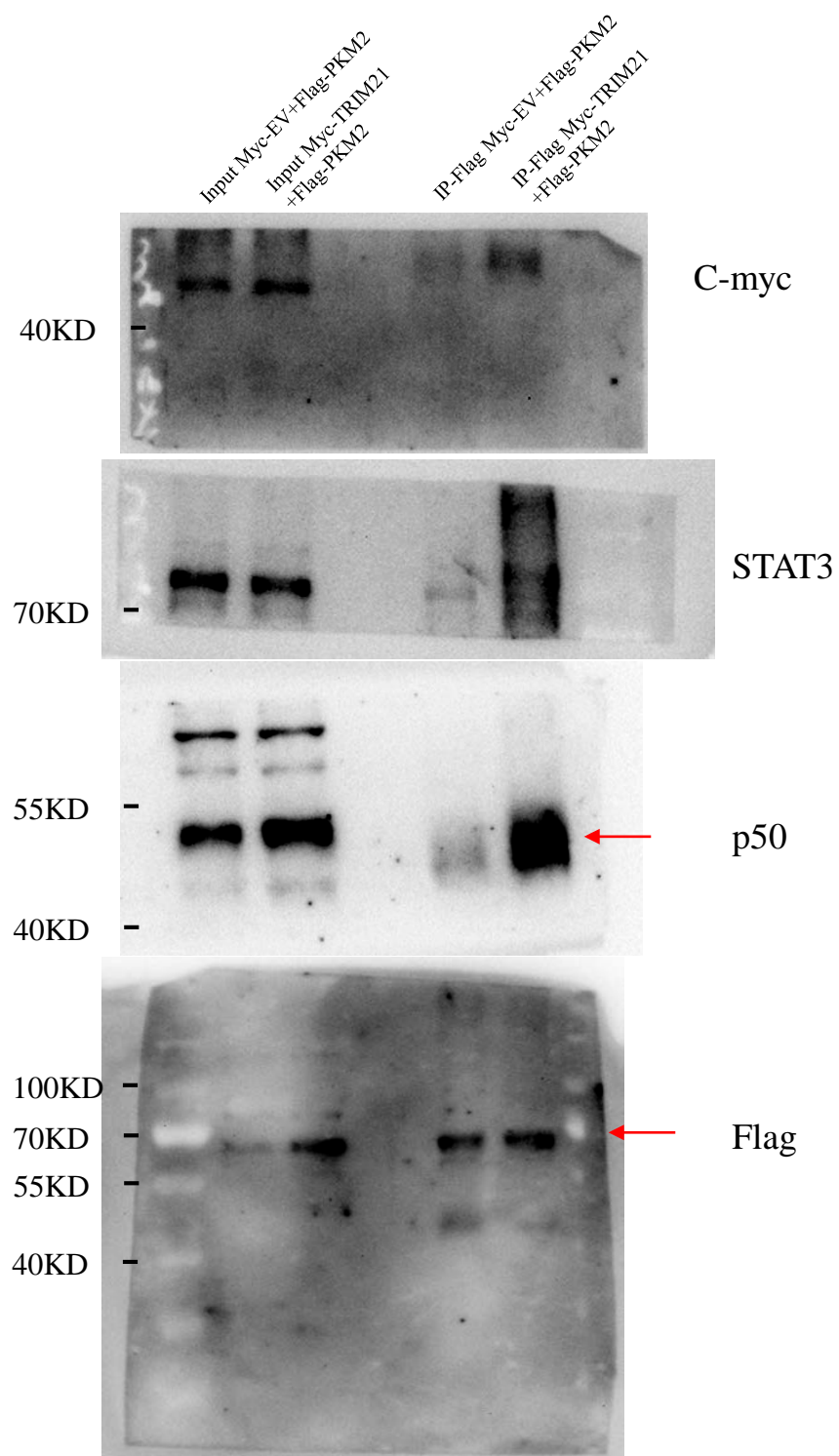

Supplement: Figure 6—source data 10. [file elife-98181-fig6-data10.pdf]

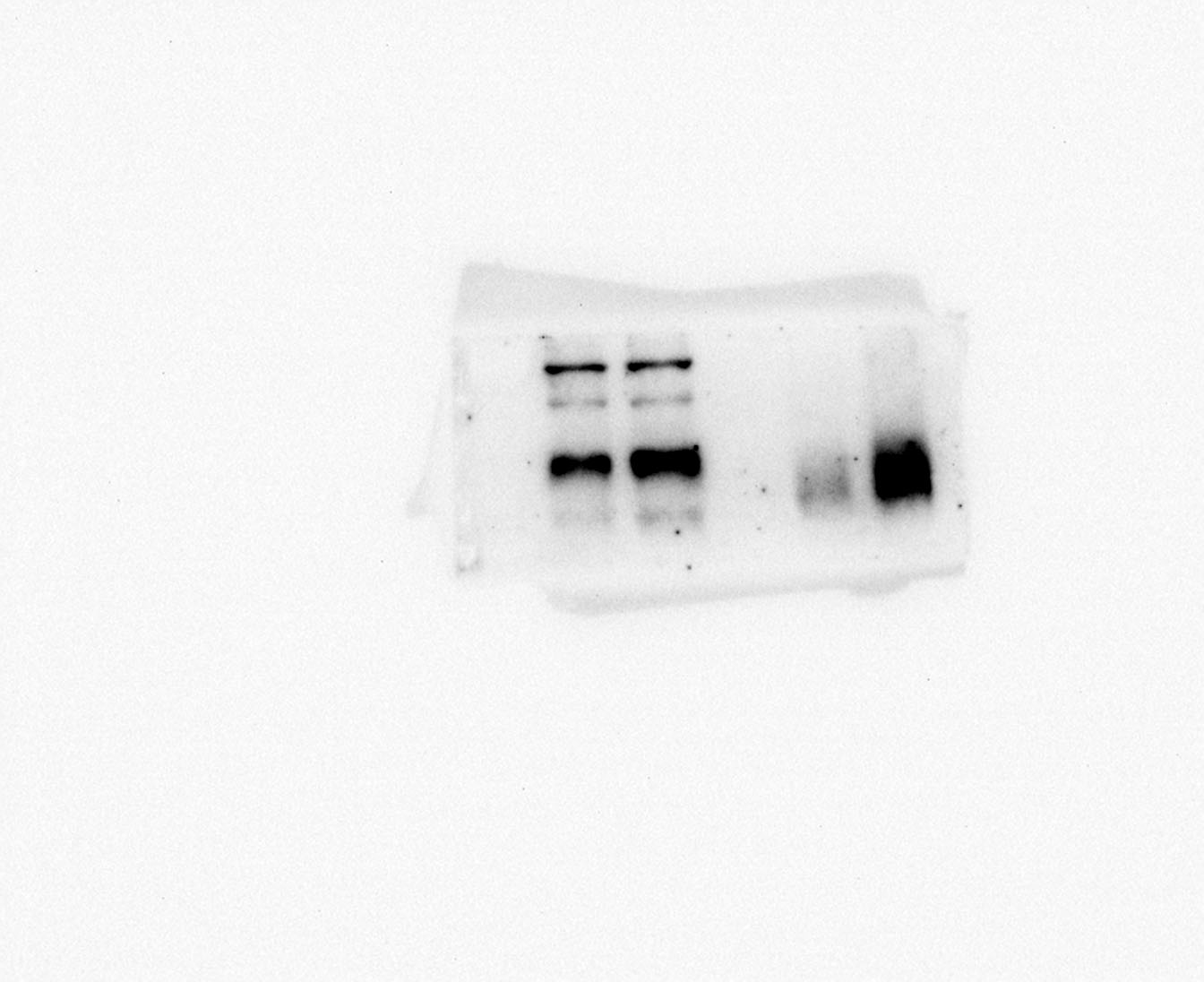

Supplement: Figure 6—source data 11. [file elife-98181-fig6-data11.zip › Figure 6-source data 11(P50).jpg]

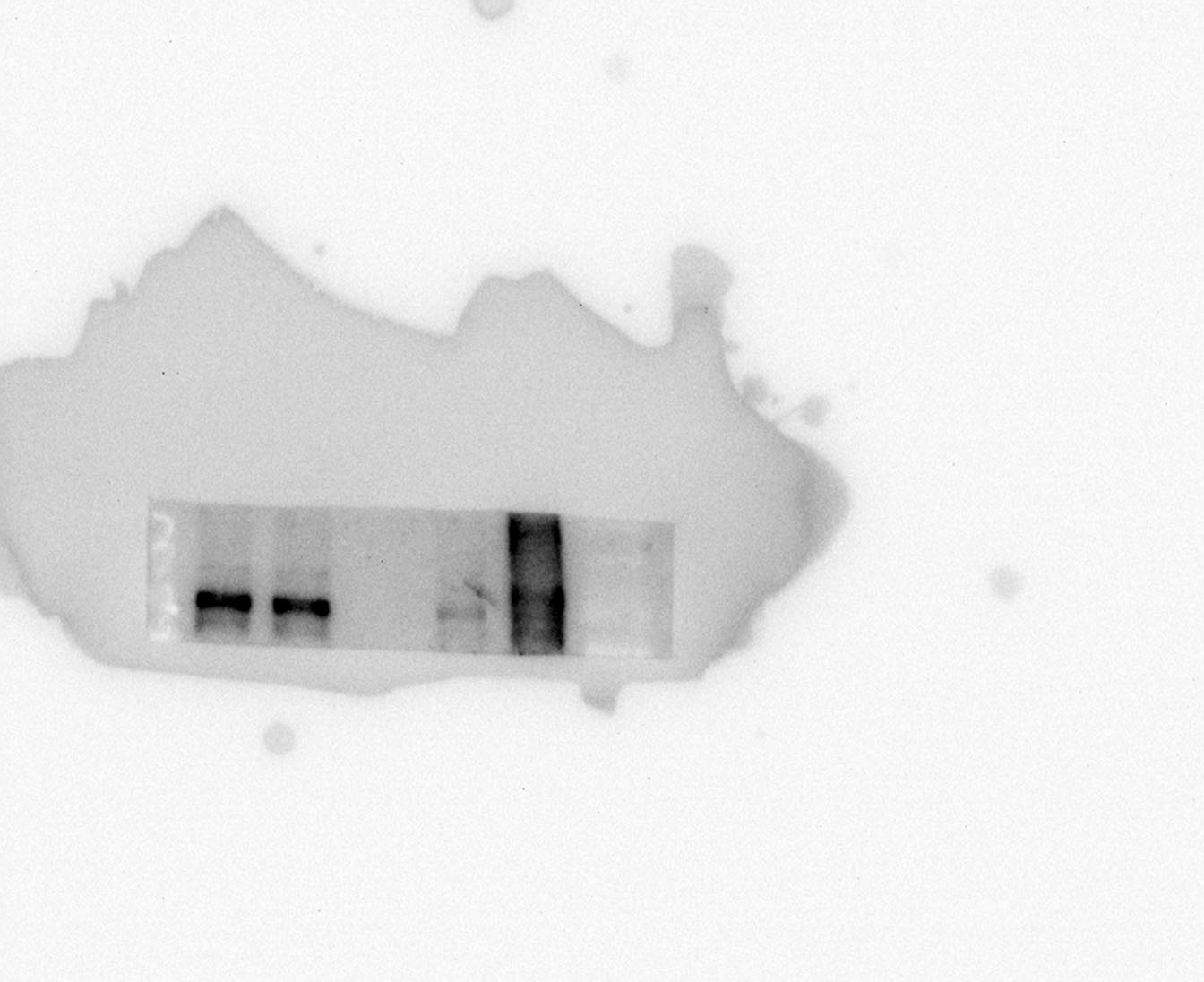

Supplement: Figure 6—source data 11. [file elife-98181-fig6-data11.zip › Figure 6-source data 11(STAT3).jpg]

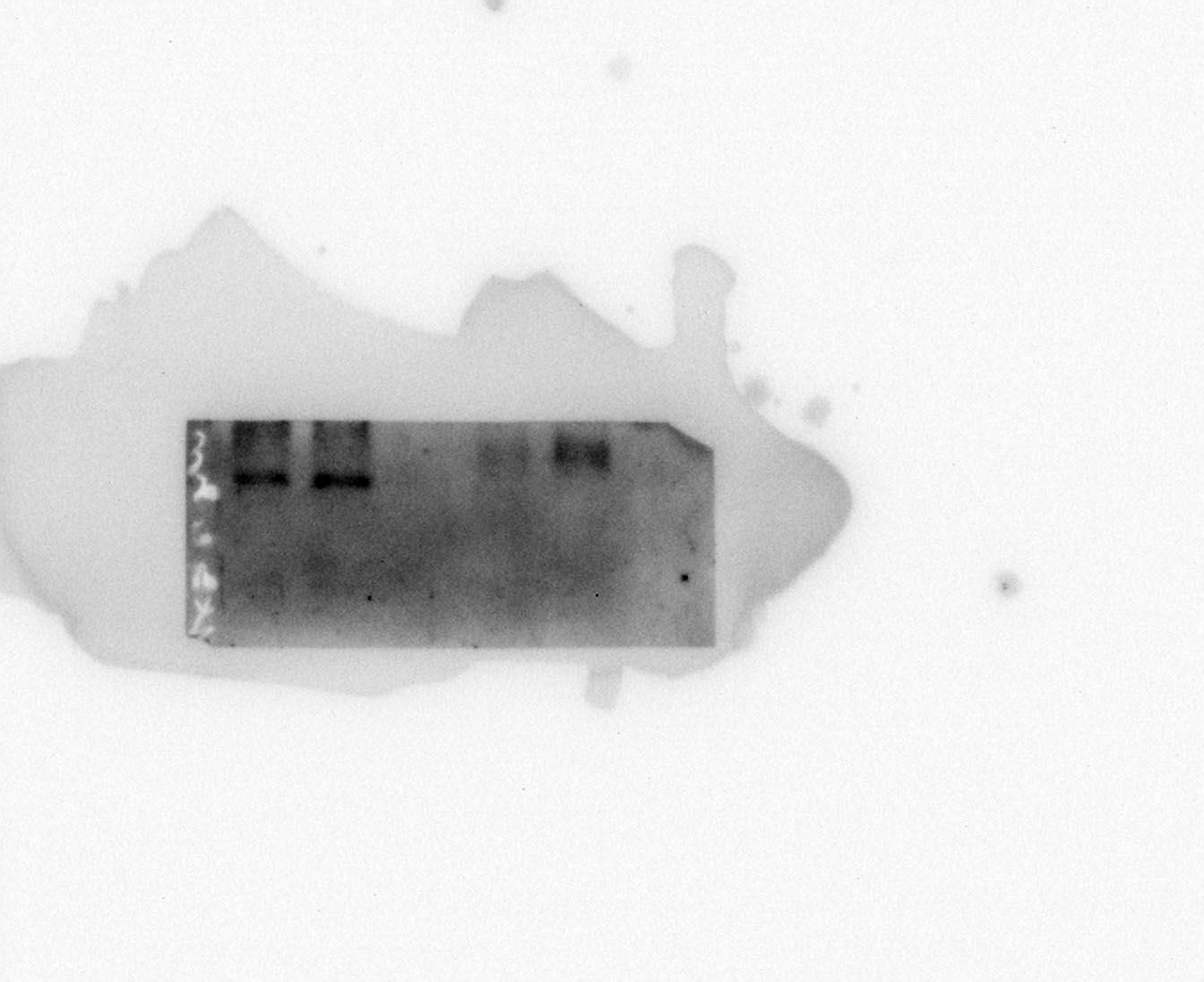

Supplement: Figure 6—source data 11. [file elife-98181-fig6-data11.zip › Figure 6-source data 11(c-myc).jpg]

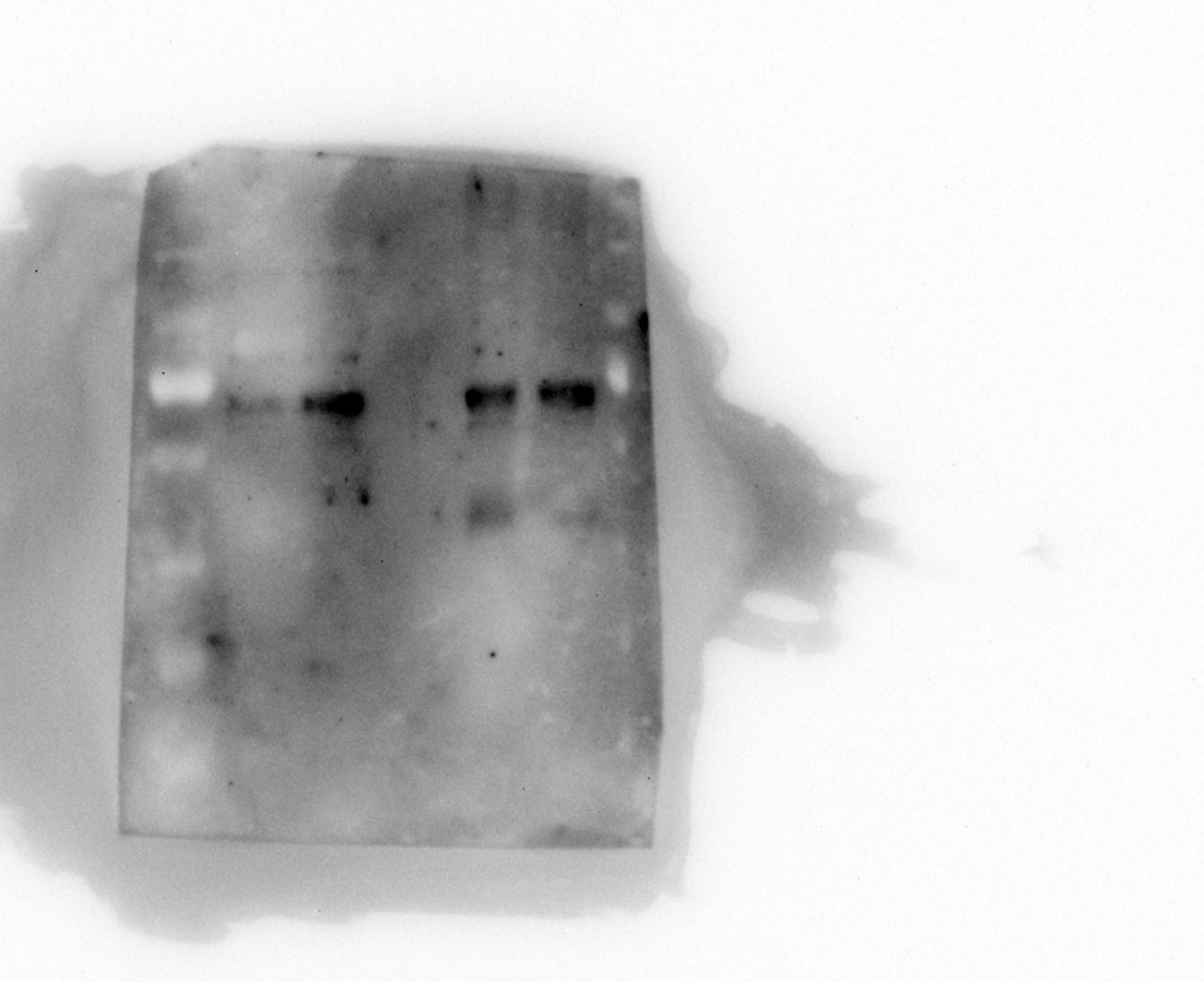

Supplement: Figure 6—source data 11. [file elife-98181-fig6-data11.zip › Figure 6-source data 11(Flag).jpg]

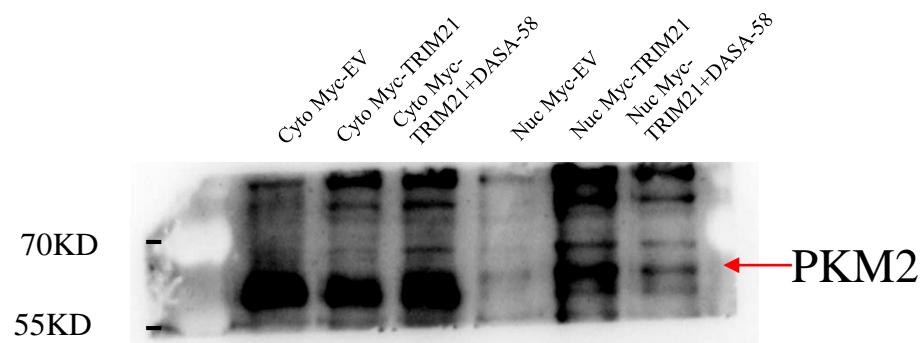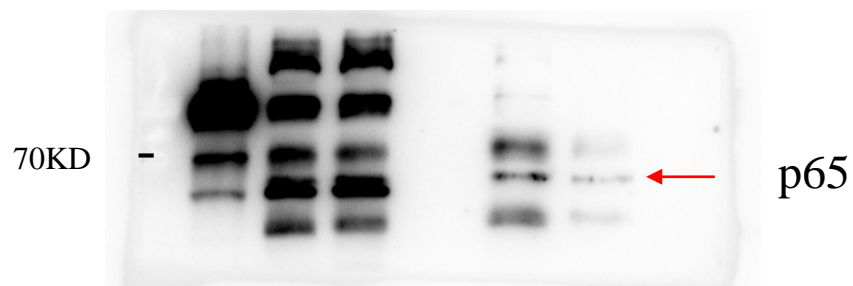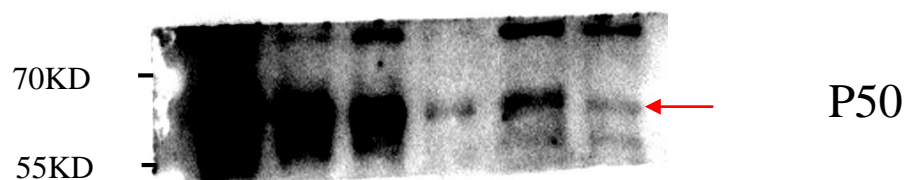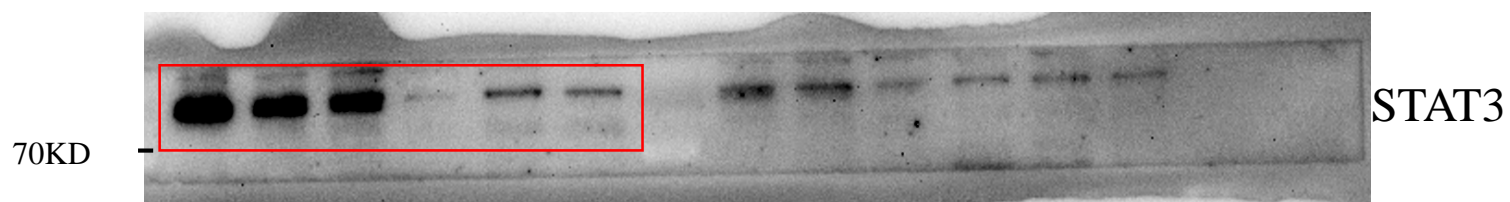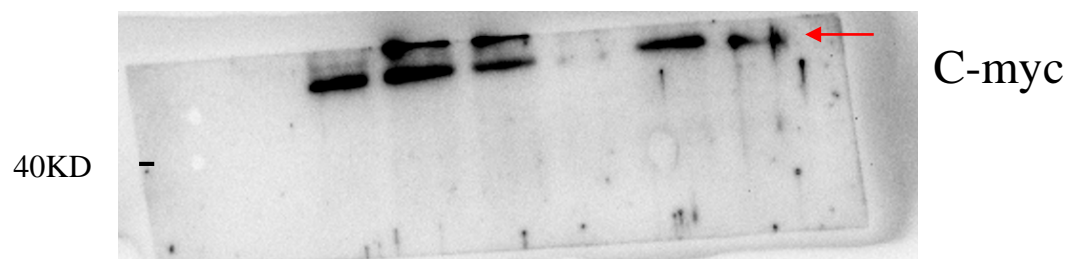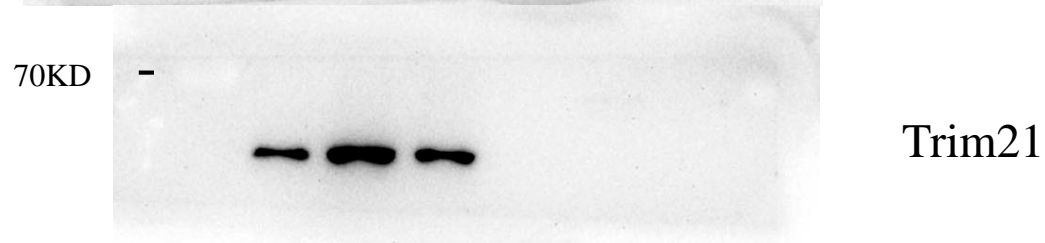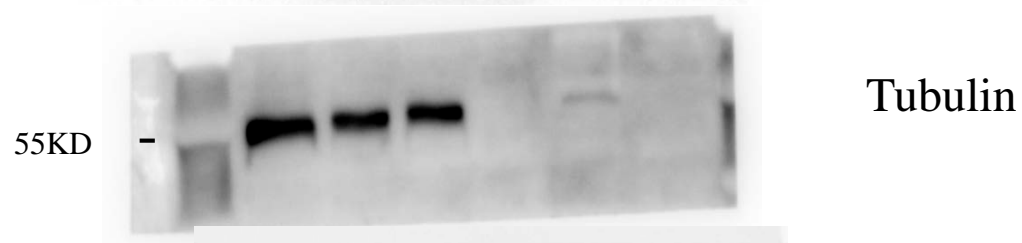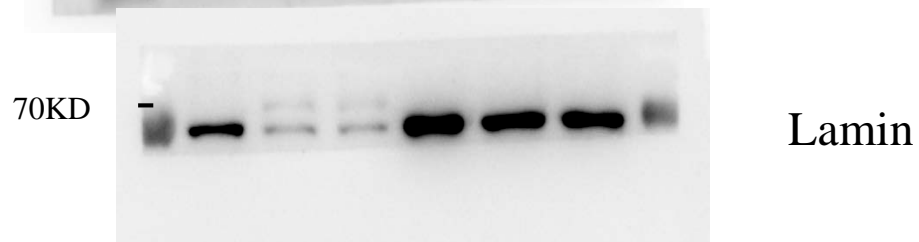

Supplement: Figure 6—source data 12. [file elife-98181-fig6-data12.pdf]

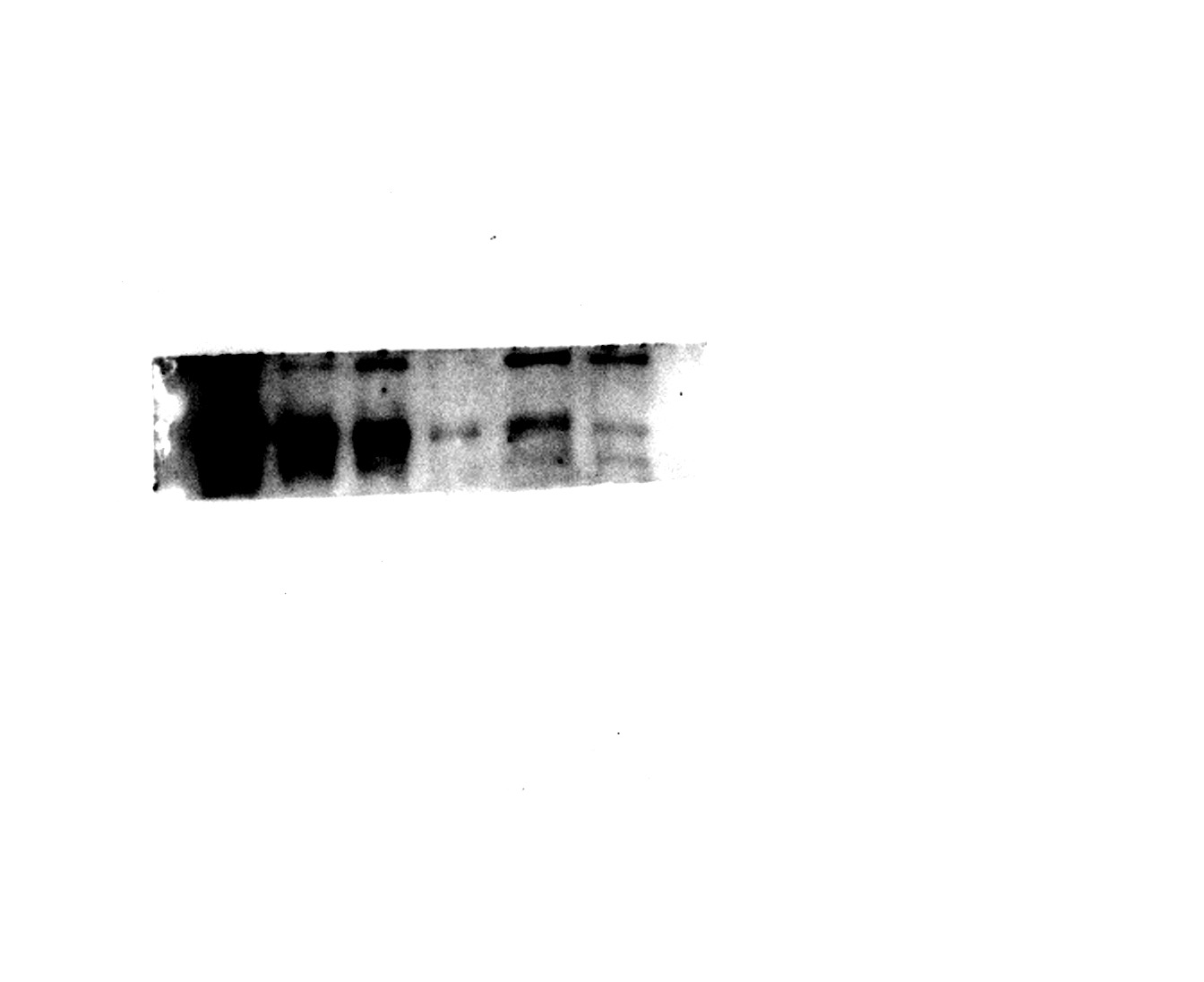

Supplement: Figure 6—source data 13. [file elife-98181-fig6-data13.zip › Figure 6-source data 13 (P50).jpg]

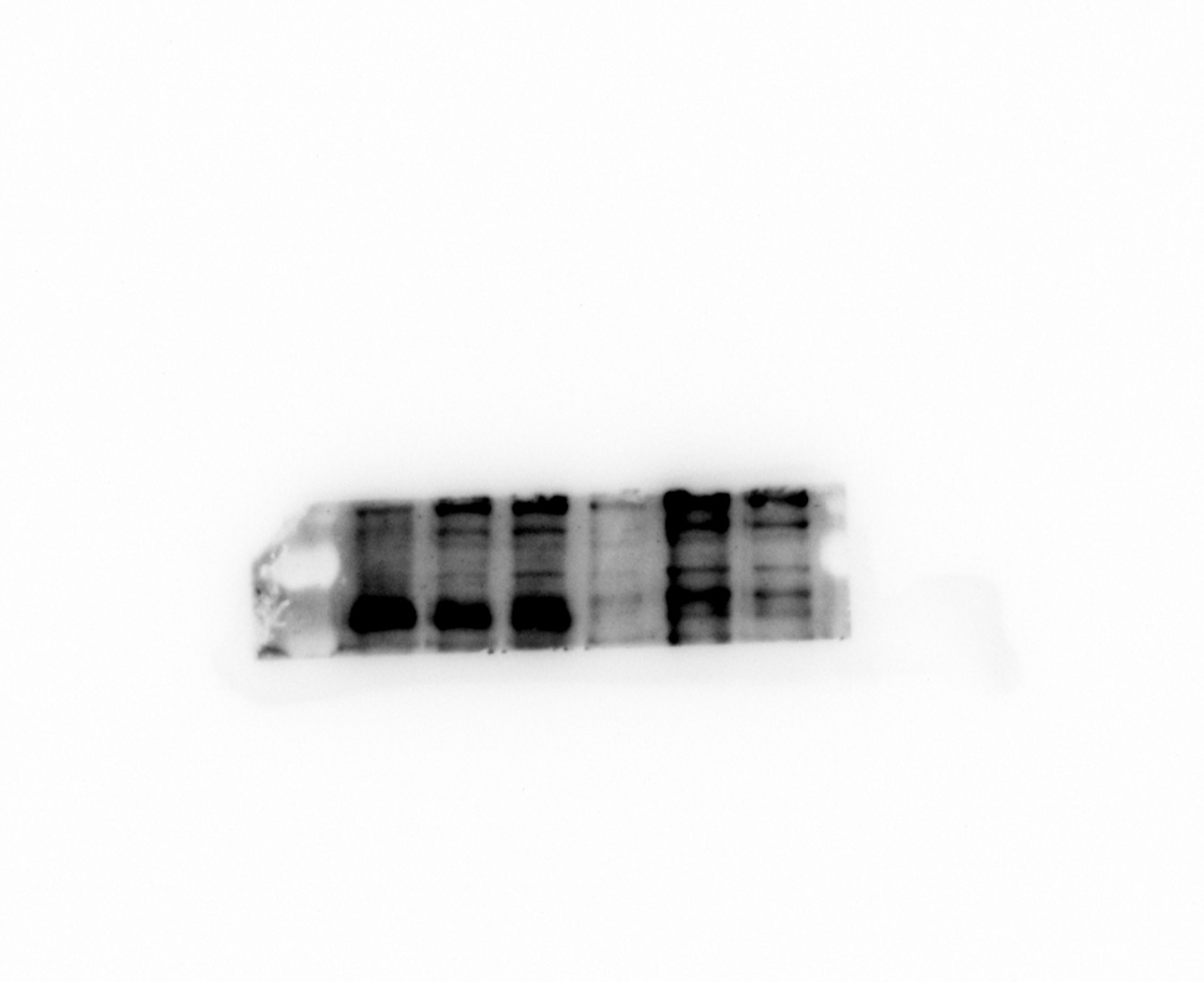

Supplement: Figure 6—source data 13. [file elife-98181-fig6-data13.zip › Figure 6-source data 13 (PKM2).jpg]

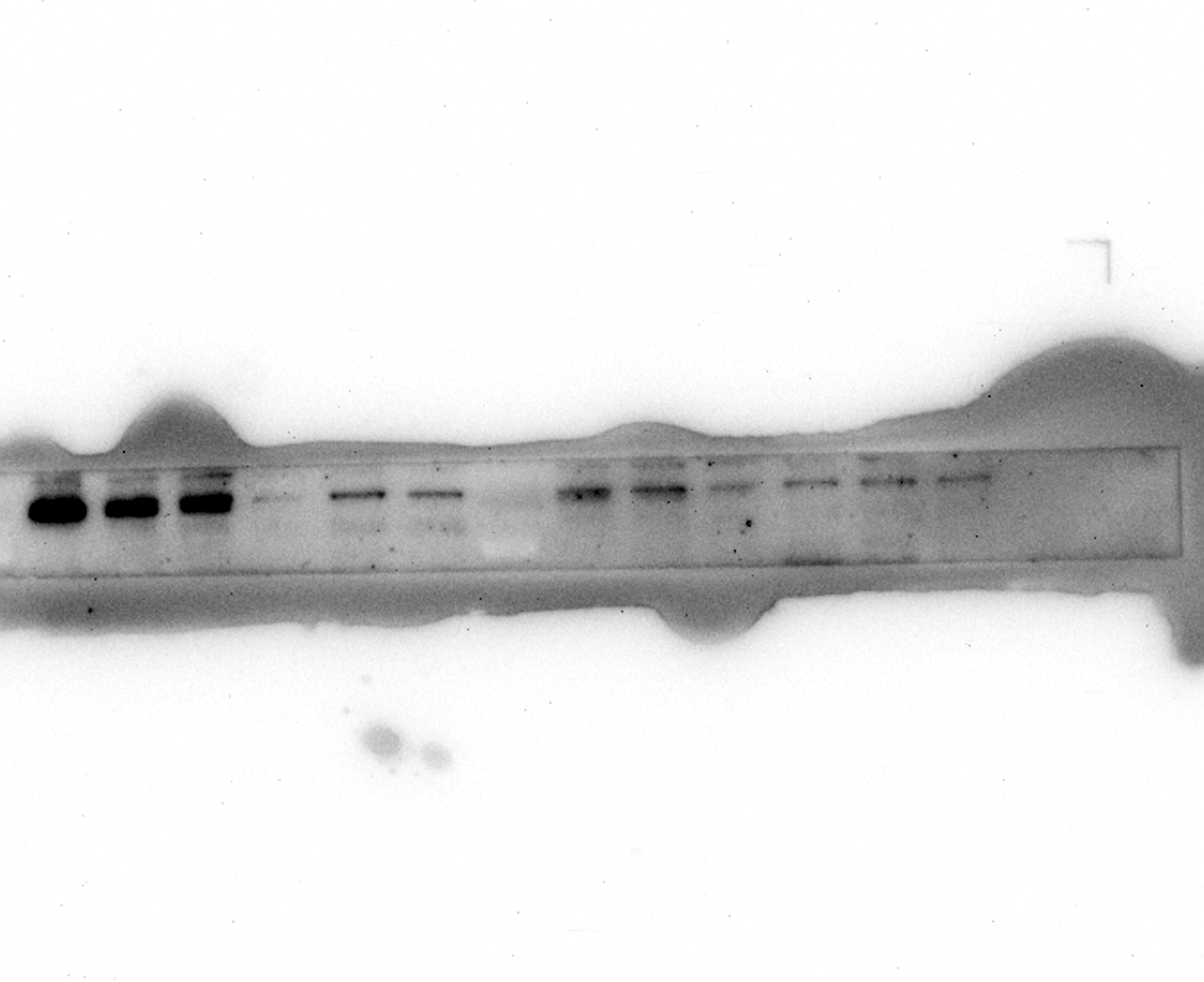

Supplement: Figure 6—source data 13. [file elife-98181-fig6-data13.zip › Figure 6-source data 13 (STAT3).tif]

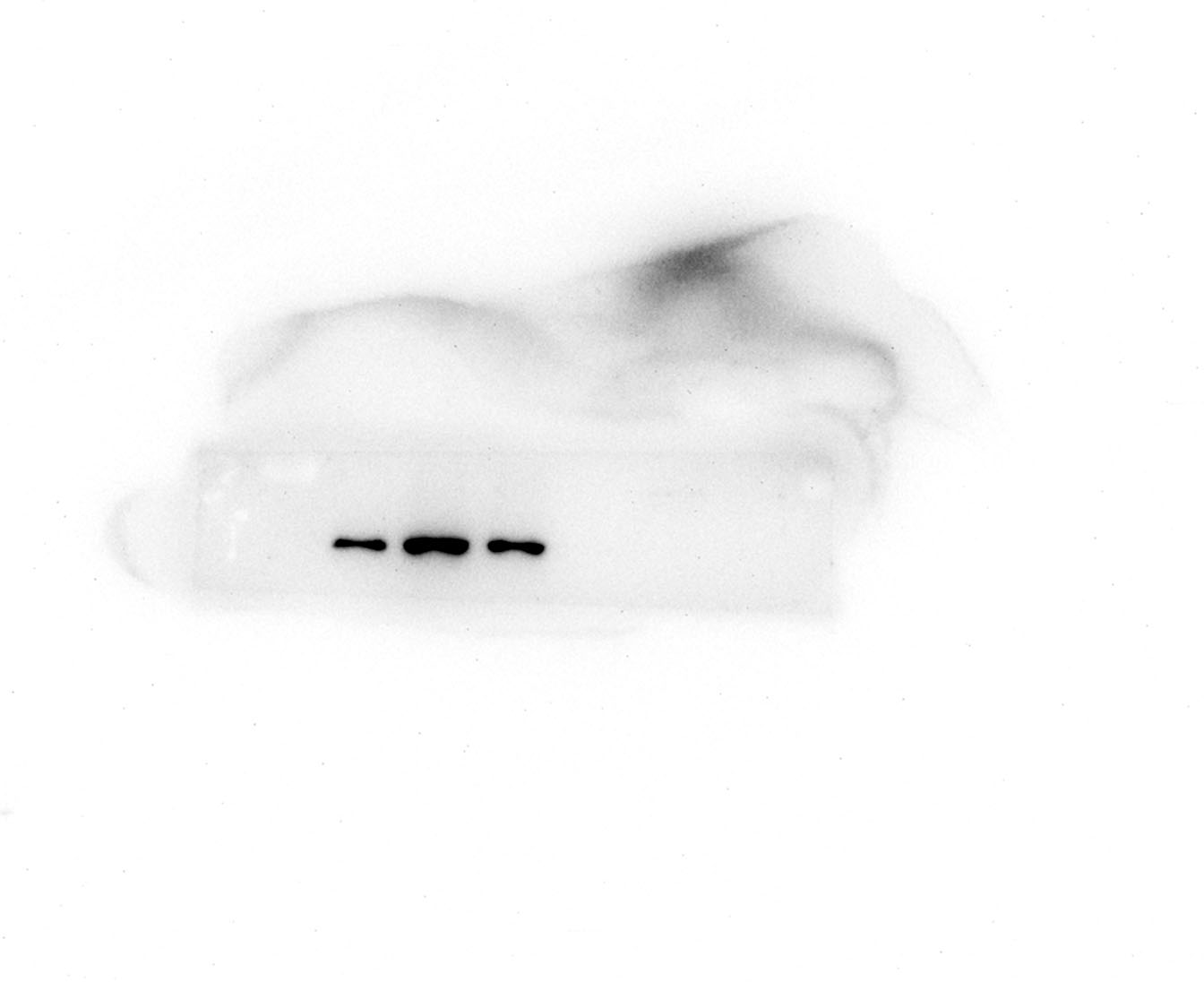

Supplement: Figure 6—source data 13. [file elife-98181-fig6-data13.zip › Figure 6-source data 13 (Trim21).jpg]

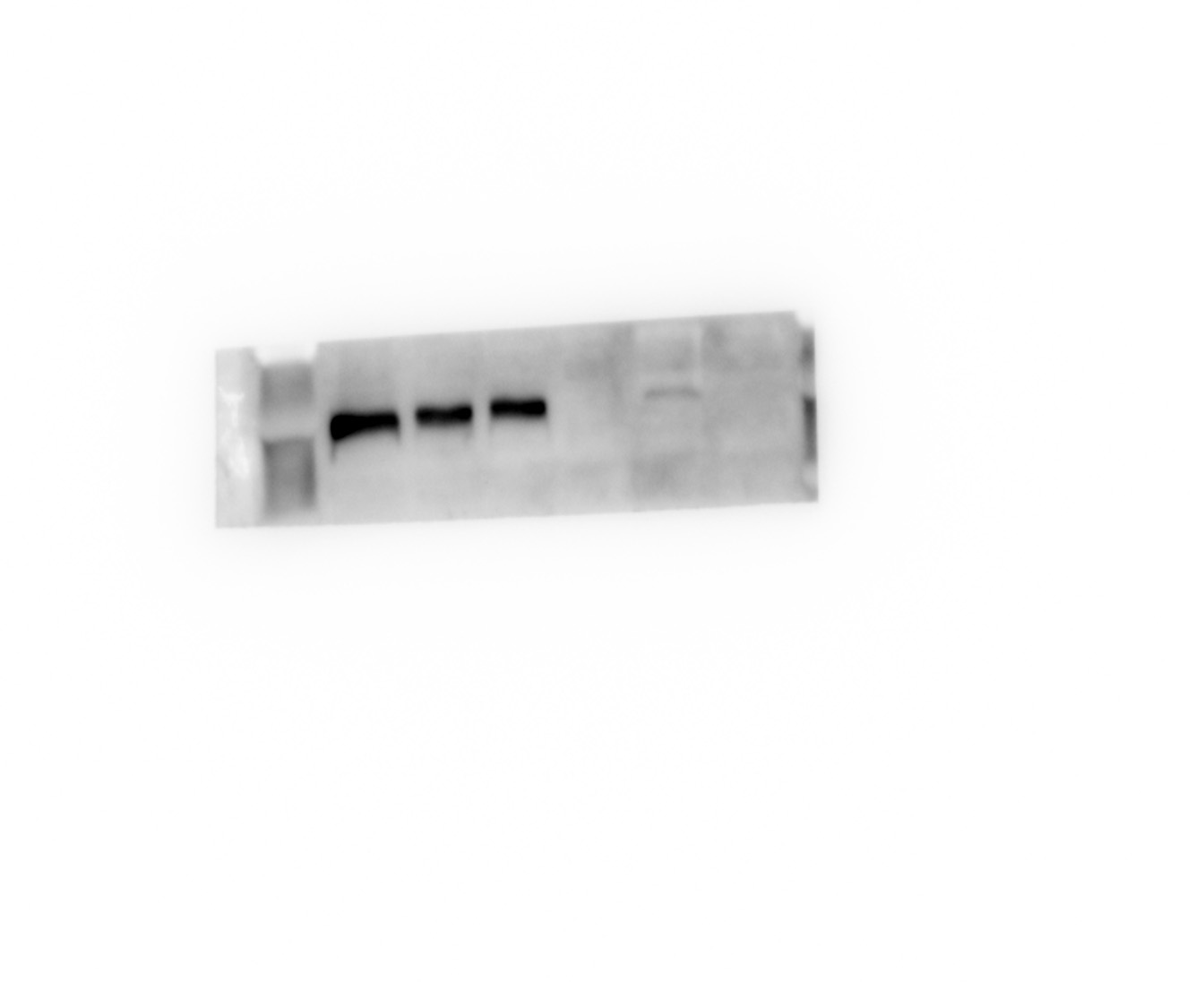

Supplement: Figure 6—source data 13. [file elife-98181-fig6-data13.zip › Figure 6-source data 13 (Tubulin).jpg]

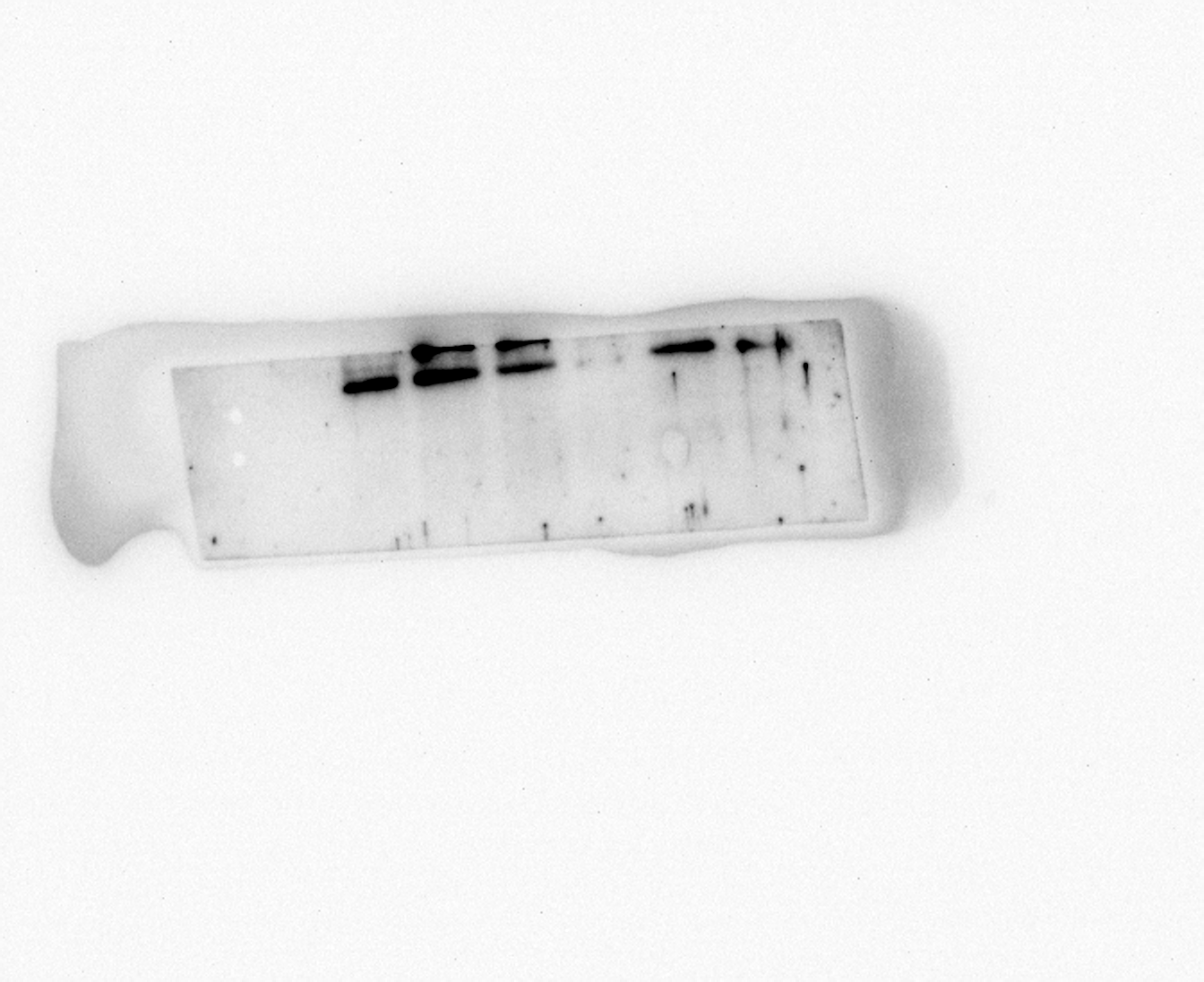

Supplement: Figure 6—source data 13. [file elife-98181-fig6-data13.zip › Figure 6-source data 13 (c-myc).tif]

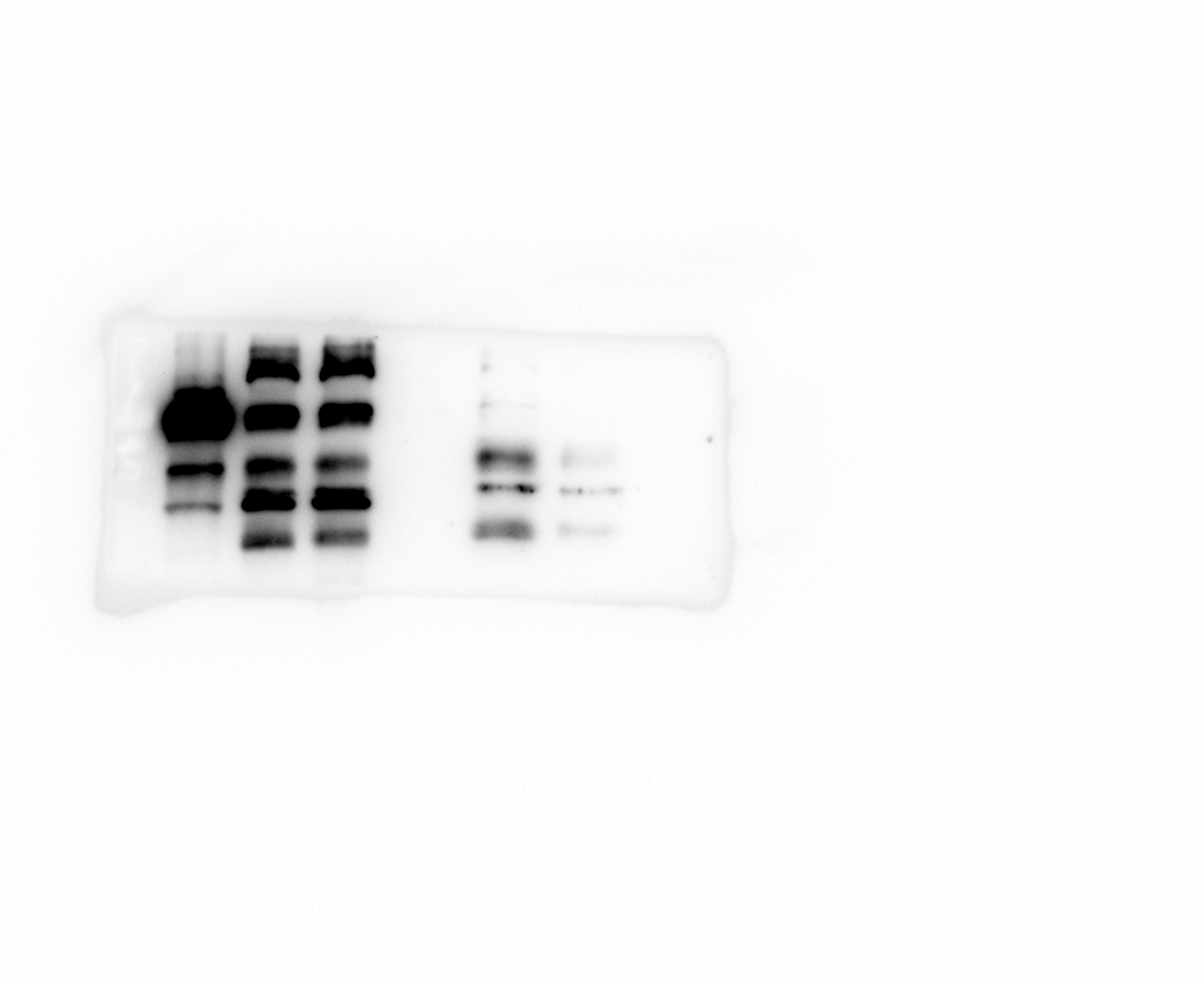

Supplement: Figure 6—source data 13. [file elife-98181-fig6-data13.zip › Figure 6-source data 13 (p65).tif]

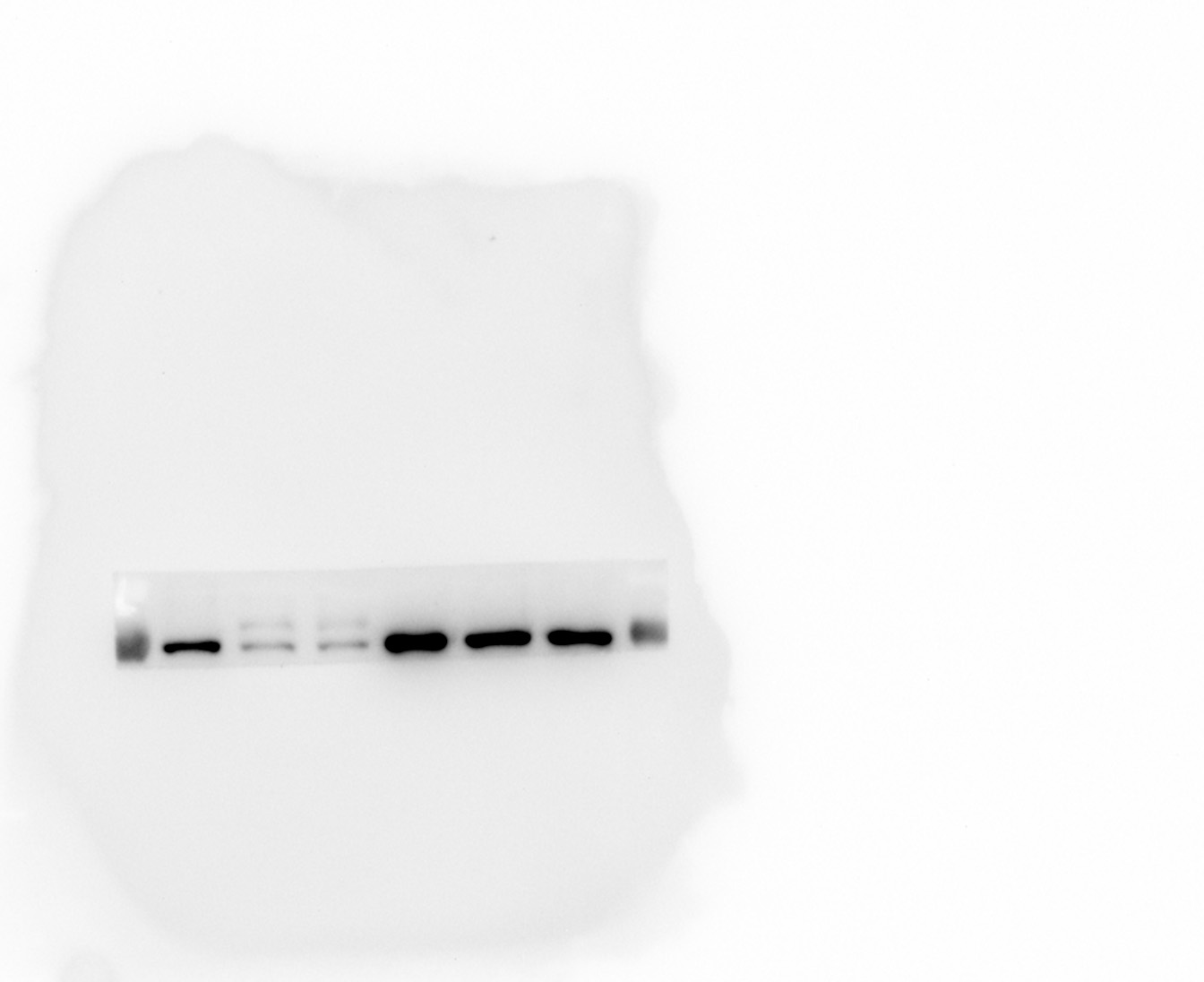

Supplement: Figure 6—source data 13. [file elife-98181-fig6-data13.zip › Figure 6-source data 13 (Lamin).jpg]

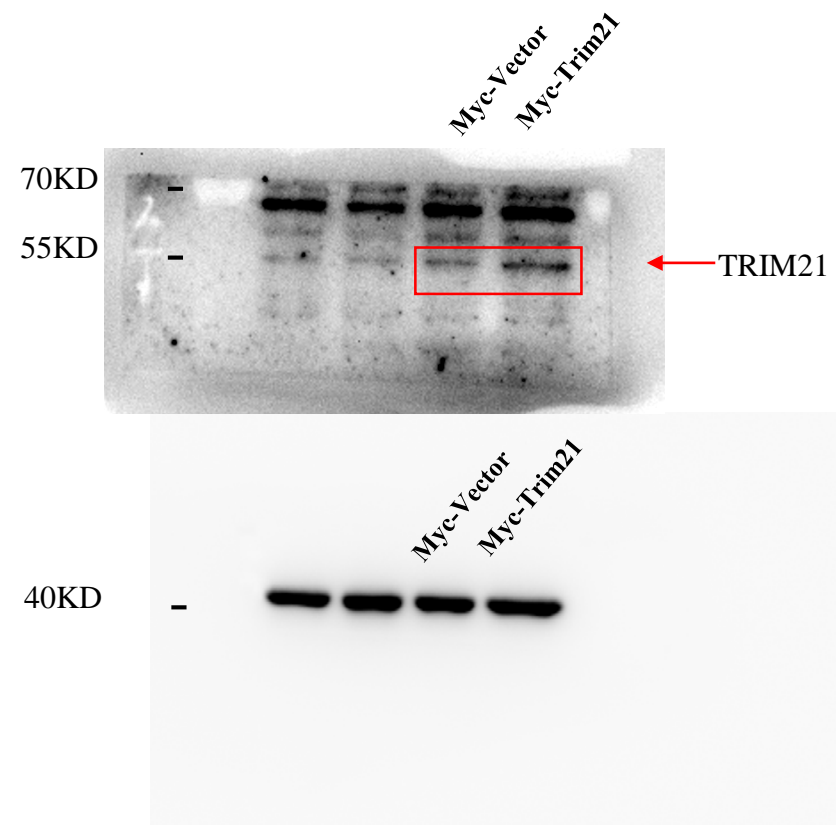

Supplement: Figure 6—figure supplement 1—source data 1. [file elife-98181-fig6-figsupp1-data1.pdf]

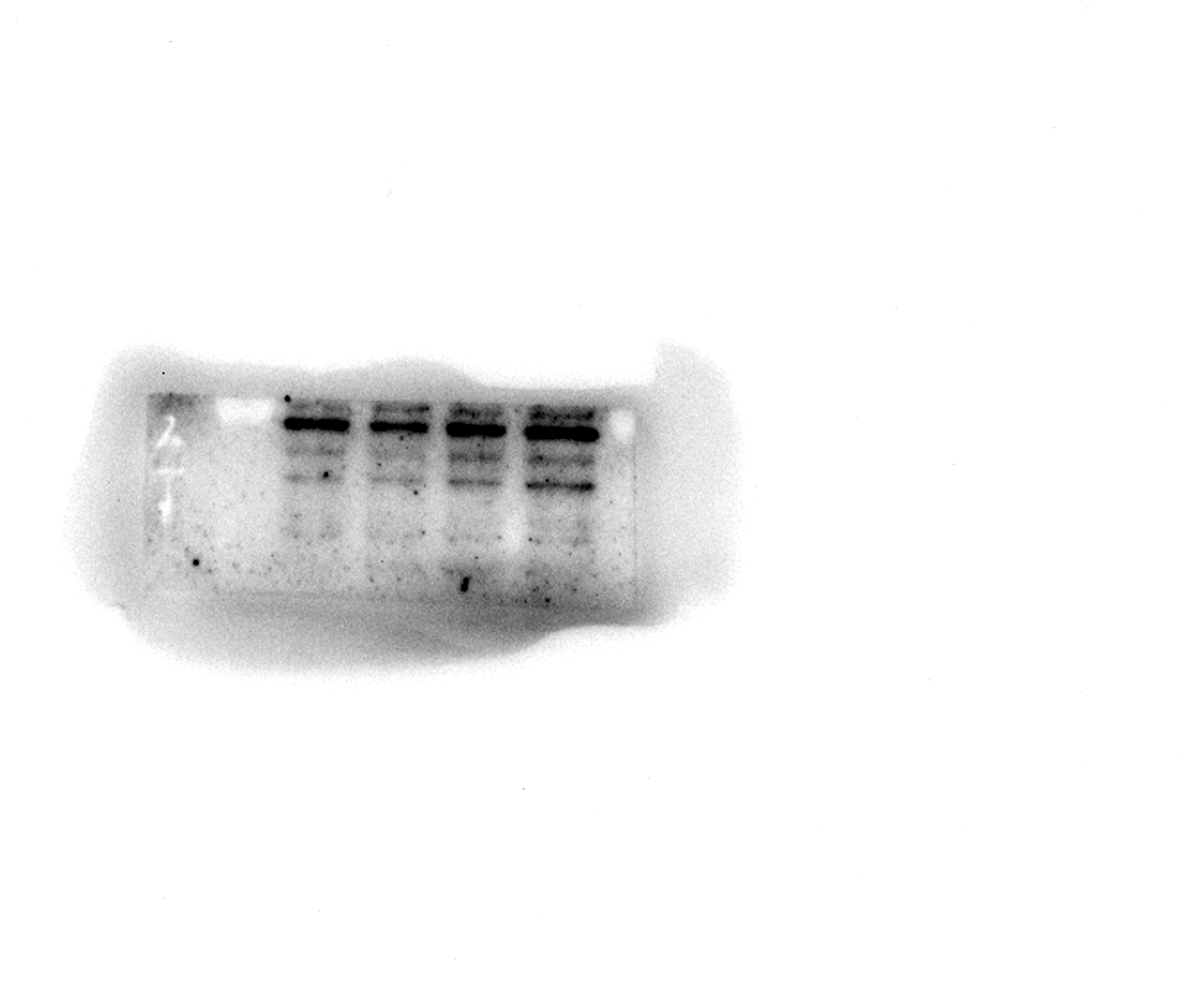

Supplement: Figure 6—figure supplement 1—source data 2. [file elife-98181-fig6-figsupp1-data2.zip › Figure 6-figure supplement 1-source data 2 (Trim21).tif]

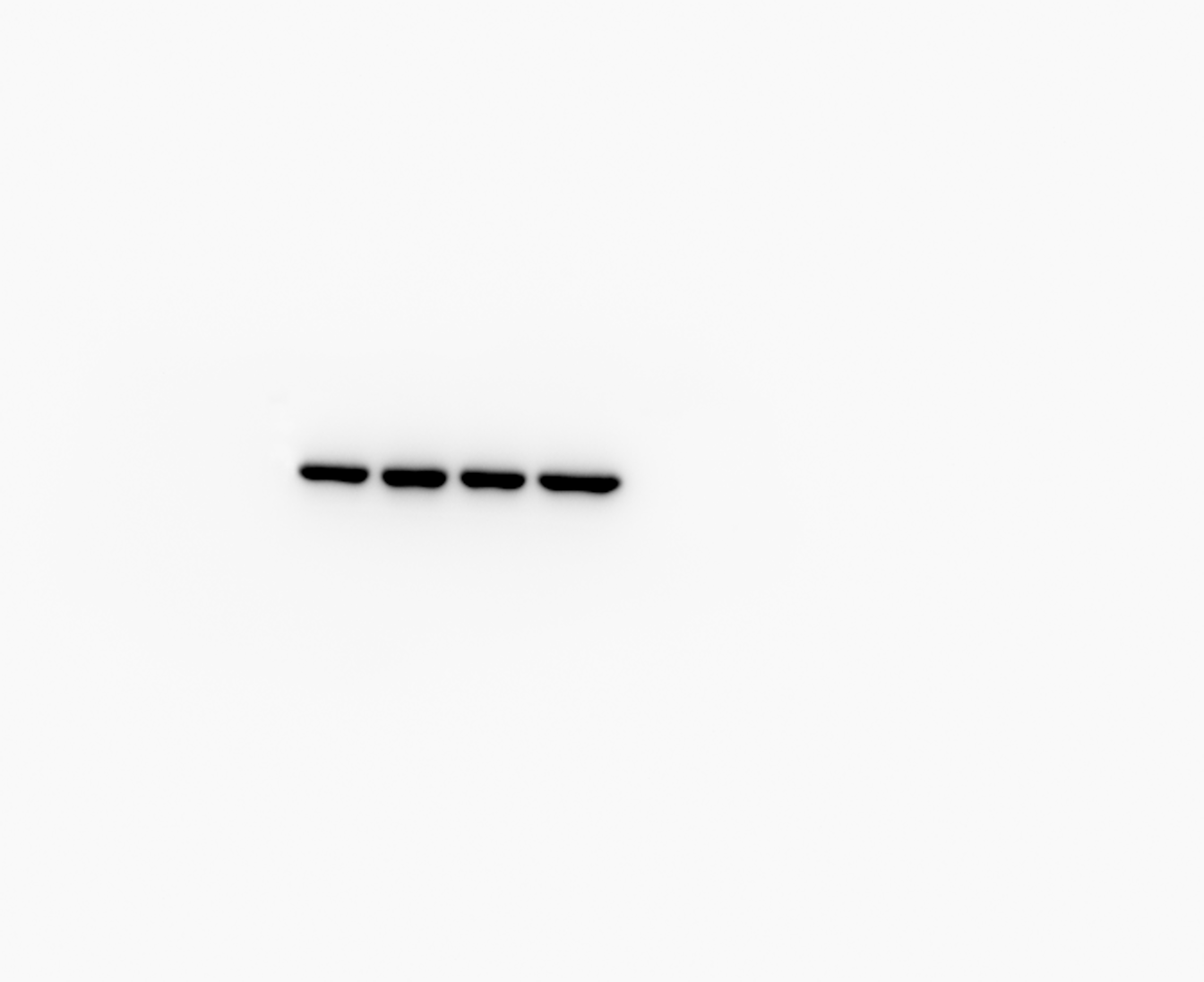

Supplement: Figure 6—figure supplement 1—source data 2. [file elife-98181-fig6-figsupp1-data2.zip › Figure 6-figure supplement 1-source data 2 (Actin).tif]

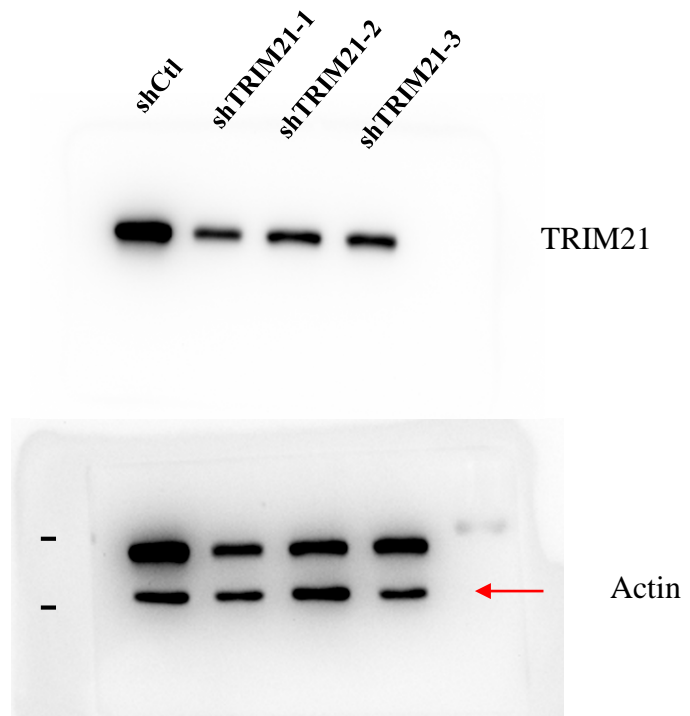

Supplement: Figure 6—figure supplement 1—source data 3. [file elife-98181-fig6-figsupp1-data3.pdf]

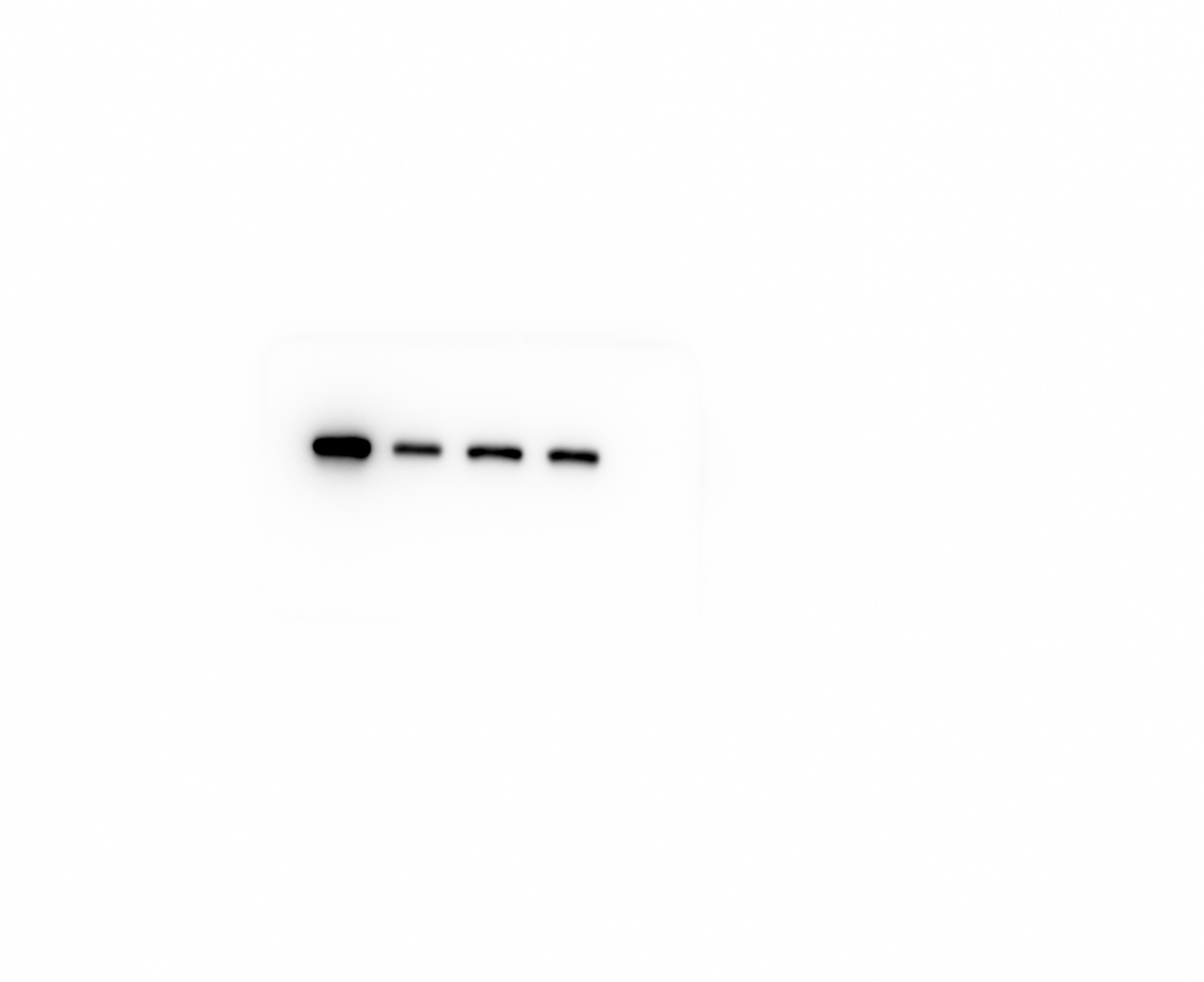

Supplement: Figure 6—figure supplement 1—source data 4. [file elife-98181-fig6-figsupp1-data4.zip › Figure 6-figure supplement 1-source data 4 (TRIM21).tif]

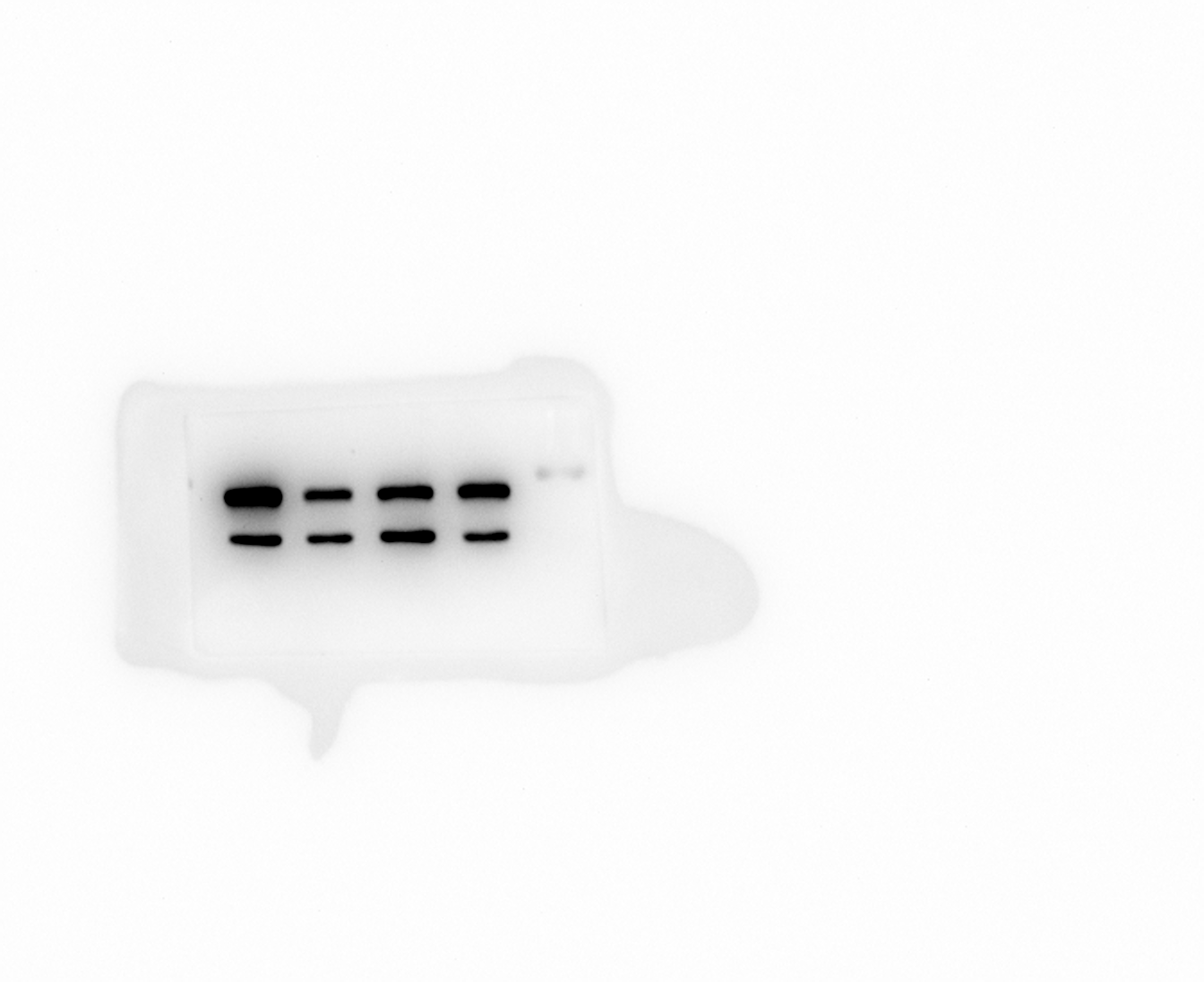

Supplement: Figure 6—figure supplement 1—source data 4. [file elife-98181-fig6-figsupp1-data4.zip › Figure 6-figure supplement 1-source data 4 (Actin).tif]
