# Supplementary material for: Upregulated expression of ubiquitin ligase TRIM21 promotes PKM2 nuclear translocation and astrocyte activation in experimental autoimmune encephalomyelitis
Source: eLife. 2024 Sep 12;13:RP98181. doi: 10.7554/eLife.98181 (PMC11392529; doi:10.7554/eLife.98181)
Supplement: Figure 4—source data 5. [file elife-98181-fig4-data5.pdf]

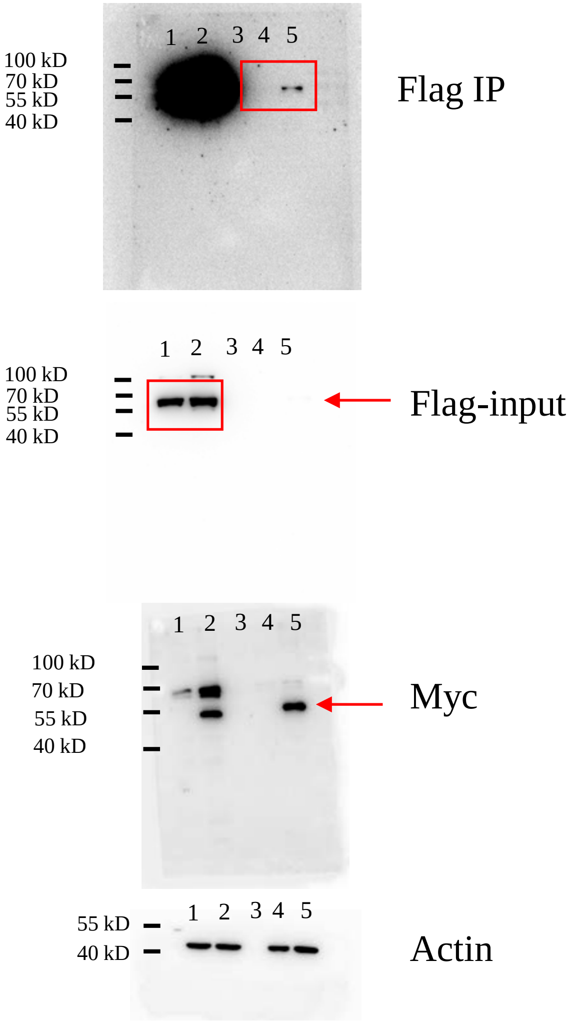

1: input Myc-vector+Flag-PKM2

2: input Myc-TRIM21+Flag-PKM2

4: IP-Myc Myc-vector+Flag-PKM2

5: IP-Myc Myc-TRIM21+Flag-PKM2
