# Supplementary material for: Upregulated expression of ubiquitin ligase TRIM21 promotes PKM2 nuclear translocation and astrocyte activation in experimental autoimmune encephalomyelitis
Source: eLife. 2024 Sep 12;13:RP98181. doi: 10.7554/eLife.98181 (PMC11392529; doi:10.7554/eLife.98181)
Supplement: Supplementary file 1. [file elife-98181-supp1.docx]

**Supplementary File 1.**

List of primers used in this study.

| Name | Primer sequences (5’-3’ orientation) |
| --- | --- |
| PKM2 | Forward: GCCGCCTGGACATTGACTC |
|  | Reverse: CCATGAGAGAAATTCAGCCGAG |
| TRIM21 | Forward: GGGAGGAGGTCACCTGTTCTA |
|  | Reverse: GGCACTCGGGACATGAACTG |
| IL-6 | Forward: GCTGGAGTCACAGAAGGAGTGGC |
|  | Reverse: GGCATAACGCACTAGGTTTGCCG |
| IL-1β | Forward: CACTACAGGCTCCGAGATGAACAAC |
|  | Reverse: TGTCGTTGCTTGGTTCTCCTTGTAC |
| TNF-α | Forward: CCTGTAGCCCACGTCGTAG |
|  | Reverse: GGGAGTAGACAAGGTACAACCC |
| Cyclin D1 | Forward: AAGTGCGTGCAGAAGGAGATTGT |
|  | Reverse: GGATAGAGTTGTCAGTGTAGATGC |
| GAPDH | Forward: AGGTCGGTGTGAACGGATTTG |
|  | Reverse: TGTAGACCATGTAGTTGAGGTCA |
